# An Unbiased Molecular Approach Using 3′-UTRs Resolves the Avian Family-Level Tree of Life

**DOI:** 10.1093/molbev/msaa191

**Published:** 2020-11-08

**Authors:** Heiner Kuhl, Carolina Frankl-Vilches, Antje Bakker, Gerald Mayr, Gerhard Nikolaus, Stefan T Boerno, Sven Klages, Bernd Timmermann, Manfred Gahr

**Affiliations:** 1 Department of Behavioural Neurobiology, Max Planck Institute for Ornithology, Seewiesen, Germany; 2 Max Planck Institute for Molecular Genetics, Sequencing Core Facility, Berlin, Germany; 3 Department of Ecophysiology and Aquaculture, Leibniz-Institute of Freshwater Ecology and Inland Fisheries, Berlin, Germany; 4 Ornithological Section, Senckenberg Research Institute, Frankfurt am Main, Germany

**Keywords:** birds, phylogenetics, bioinformatics, transcriptomes, vocal learning, 3′-UTR

## Abstract

Presumably, due to a rapid early diversification, major parts of the higher-level phylogeny of birds are still resolved controversially in different analyses or are considered unresolvable. To address this problem, we produced an avian tree of life, which includes molecular sequences of one or several species of ∼90% of the currently recognized family-level taxa (429 species, 379 genera) including all 106 family-level taxa of the nonpasserines and 115 of the passerines (Passeriformes). The unconstrained analyses of noncoding 3-prime untranslated region (3′-UTR) sequences and those of coding sequences yielded different trees. In contrast to the coding sequences, the 3′-UTR sequences resulted in a well-resolved and stable tree topology. The 3′-UTR contained, unexpectedly, transcription factor binding motifs that were specific for different higher-level taxa. In this tree, grebes and flamingos are the sister clade of all other Neoaves, which are subdivided into five major clades. All nonpasserine taxa were placed with robust statistical support including the long-time enigmatic hoatzin (Opisthocomiformes), which was found being the sister taxon of the Caprimulgiformes. The comparatively late radiation of family-level clades of the songbirds (oscine Passeriformes) contrasts with the attenuated diversification of nonpasseriform taxa since the early Miocene. This correlates with the evolution of vocal production learning, an important speciation factor, which is ancestral for songbirds and evolved convergent only in hummingbirds and parrots. As 3′-UTR-based phylotranscriptomics resolved the avian family-level tree of life, we suggest that this procedure will also resolve the all-species avian tree of life

## Introduction

The phylogeny of birds has been intensively studied during the last 20 years using anatomical and molecular data. Several recent molecular approaches, based either on genomes of a limited number of bird families (Jarvis et al. 2014; Suh et al. 2015) or on a large number of bird families, but only a selection of molecular sequences (Ericson et al. 2006; Hackett et al. 2008; Prum et al. 2015), delivered important new insights in the avian tree of life, such as the close relationships of passerines, parrots, and falcons. However, these studies also yielded strongly conflicting results or had low statistical support for a number of neoavian clades (Prum et al. 2015). This was interpreted either as a result of a hard-to-resolve ancient diversification of modern birds (Jarvis et al. 2014) or as a result of incomplete lineage sorting (Suh et al. 2015). In particular, these studies did not contain all nonpasserine families. Furthermore, although oscine passerines, the songbirds, constitute the majority of the extant avian diversity, previous studies aiming on resolving the entire avian tree of life have only included limited numbers of oscine taxa in their analyses (Jarvis et al. 2014; Prum et al. 2015). The scope of broader previous approaches to songbird families using molecular information was based either on few selected genes and little sequence information (Barker et al. 2004), or these analyses included no or few nonpasserine species (Barker et al. 2004; Oliveros et al. 2019).

Here, we present a family-level avian tree of life that is based on transcriptome sequences or their genomic orthologs and that involved 221 family-level taxa of which 209 are among the 227 families recognized by both the International Ornithological Union (IOU) (Gill and Donsker 2017) and the Handbook of the Birds of the World (HBW) (Del Hoyo and Collar 2014, 2016), which is a widely used standard reference covering all extant avian species (supplementary table S1, Supplementary Material online). In particular, our analysis covers all 106 currently recognized nonpasserine families.

Previous molecular approaches to the avian tree of life resulted in important differences dependent on the usage of coding nuclear genome sequences, noncoding nuclear genome sequences, a mixture of both, or the use of mitochondrial genomes (Hackett et al. 2008; Jetz et al. 2012; Jarvis et al. 2014; Prum et al. 2015; Reddy et al. 2017). Noncoding sequences seem favorable to resolve the avian tree of life, as increased taxon sampling had a positive impact on phylogenetic procedures in the case of noncoding sequences, but not in the case of coding sequence sampling (Reddy et al. 2017; Houde et al. 2019).

By contrast, we based our phylogeny on transcriptomes, which is a novel approach. Transcriptomes are composed of coding and noncoding sequences; the latter include 3′-untranslated regions (3′-UTRs), 5′-untranslated regions (5′-UTRs), noncoding RNAs, and, unspecifically, few intronic and intergenic sequences. From the sequenced RNA, we produced de novo assembled transcriptomes and mapped them to the backbone of the recently published canary genome (Frankl-Vilches et al. 2015). We used the canary genome because it is well annotated for coding sequences and UTR sequences. For some species (representing their respective families), we used available genomes in order to extract coding and UTR sequences that were homologous to the gene models of the canary and many other taxa. Thus, we were able to produce phylogenetic trees from varying amounts of coding or noncoding sequences in order to evaluate at which taxonomic level (order, family, genus) these trees differ and which might be the least error-prone and statistically most stable representation of the avian phylogeny.

3′-UTRs are located directly downstream of protein-coding DNA sequences and contain *cis*-regulatory elements that control mRNA stability, mRNA expression levels, mRNA localization, protein–protein interactions, and diversification of protein function (Mayr 2016; Mayr C 2017). New sequencing technologies and genome-wide analysis via ChIP-on-chip and Chip-seq showed that up to 5% of DNA-protein binding sites are located within 3′-UTRs (Stergachis et al. 2013; Peña-Hernández et al. 2015; Chung et al. 2018; Ferdous et al. 2018; Burgess et al. 2019). The increased and variable length of alternative 3′-UTRs, as they are observed in vertebrates, and the amount and types of binding sites for transcription factors and RNA binding proteins are expected to promote species-specific tissue-specific gene expression (Sandberg et al. 2008; Lianoglou et al. 2013; Cohen et al. 2014; Mayr C 2017; Lee and Mayr 2019; Xu et al. 2019). In relation to the 3′-UTR-based tree, we present 3′-UTR sequences that appear to be higher-level taxon- (“order-,” “family-,” “genus-”) specific.

In summary, we constructed several family-level phylogenies through an unbiased approach in which we included molecular sequences derived from transcriptomes or their genomic orthologs in a concatenation bioinformatics procedure including all nonpasserine families. One of these phylogenies, the 3′-UTR-tree, for the first time shows a stable and highly significant relationship between all avian orders and their respective nonpasserine and passerine family-level taxa.

## Results

### The Noncoding 3′-UTR Sequences Yield a Stable Molecular Tree of Avian Family-Level Taxa

The transcriptomes of 308 species were assembled de novo, clustered, and integrated with publicly available transcriptomes (*n* = 80) and orthologous sequences derived from available genomic data (*n* = 59 bird species; *n* = 2 alligator species) and newly generated genomic data (*n* = 7) (supplementary table S1 and fig. S1, Supplementary Material online). The new genome assemblies provided in this study were sequenced to ∼60-fold, which, although resulting in fragmented genome assemblies (N50 contig size of 10–40 kb, see supplementary table S2*C*, Supplementary Material online), were sufficient for whole-genome alignment and phylogenetic tree inference.

We performed several tests to estimate the quality of each de novo transcriptome assembly (supplementary table S2, Supplementary Material online). In summary, the median N50 transcript length of the transcriptomes was 2,698 ± 811 bp (mean ± SD) and the median of complete BUSCO genes (Aves data set) was 53.5±19.4% (mean±SD). The median number of nucleotides aligned to the reference genome’s 3′-UTRs and coding regions were 7.9 ± 3.6 and 9.0 ± 3.7 Mb, respectively (supplementary table S2, Supplementary Material online). We found differences between tissue types used for RNA extraction. Transcriptomes from brain exhibited highest numbers of nucleotides aligned to 3′-UTR and CDS of the reference genome (13.6 and 14.0 Mb, respectively) (supplementary fig. S2*A* and table S2, Supplementary Material online). Miscellaneous tissue types that were termed “body” were neither brain nor blood samples and mainly represent samples from museum specimens. Statistically, the transcriptome size of brain tissue was similar to skin tissue, whereas blood, liver, skin, and muscle tissue had similar transcriptome sizes (see supplementary fig. S2*A* and table S2*D*, Supplementary Material online for statistical data). As brain samples were scattered across 11 orders and all but four families (four oscines) were represented by multiple tissues, it is highly unlikely that tissue type affected the phylogenies. In particular, due to the use of a gappiness criteria (see below) and the necessary alignment in a phylogenetically basal species (the ostrich, *Struthio camelus*), transcripts that are expressed in only some species or higher-level taxa or are expressed in only certain tissue types were filtered out of the data set used for the calculation of the phylogenetic trees. The aligned portions of tissue-specific transcriptomes remaining after applying the gappiness criteria of 90–110 missing samples were very similar in size (supplementary table S2*E* and fig. S2*B*, Supplementary Material online for statistical data).

The 452 avian assemblies, which represent 429 species (389 genera) of 221 avian family-level taxa and all 35 currently recognized orders were aligned to the canary reference genome (supplementary fig. S1, Supplementary Material online) and divided into coding and noncoding sequences (intergenic, intronic, 5′-UTR, 3′-UTR). Due to the nature of transcriptomes, 3′-UTRs represented >90% of all sequences in our noncoding multiple alignments and provided numbers of sequences comparable to the codon-aligned coding sequences alignments. Thus, we compared molecular trees based on noncoding sequences (3′-UTR), on coding sequences (CODON), on the first and second nucleotide of a codon removing the highly variable third position of the codons (CODON12), and based on the corresponding translated amino acid sequence (AAS).

**Fig. 1.**
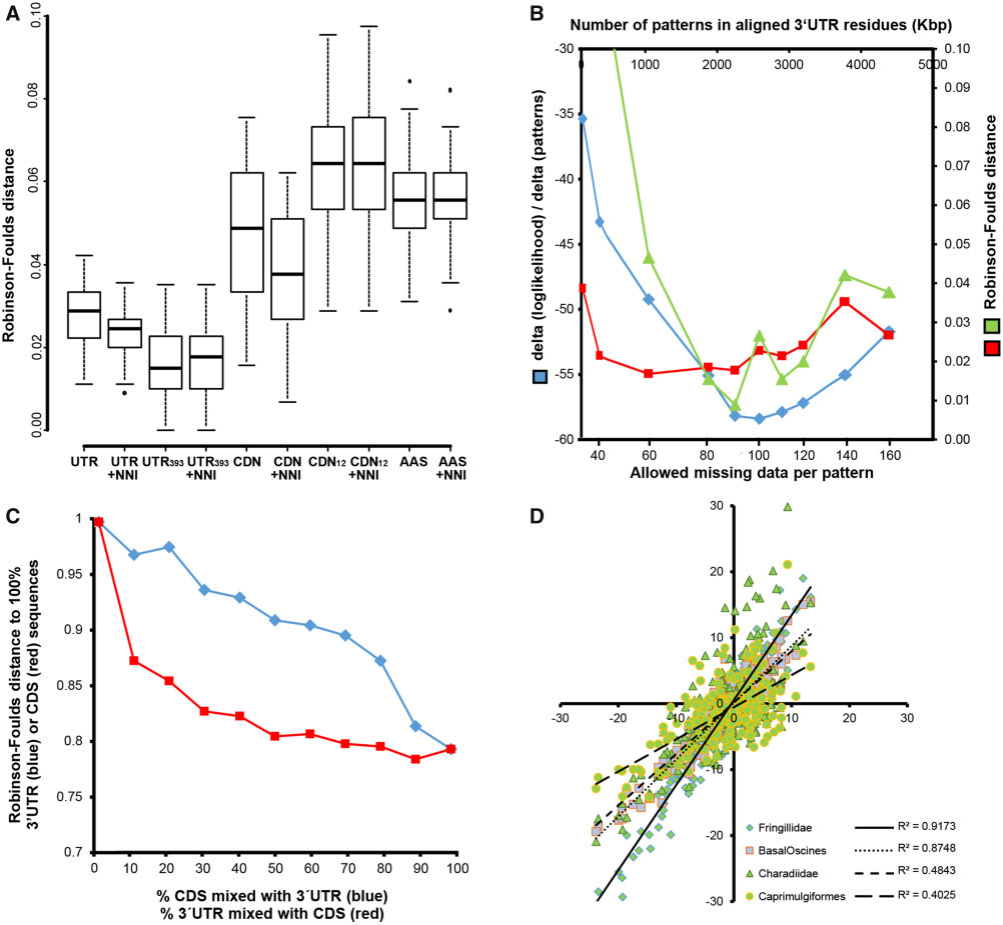
Analysis of tree topology congruency for different noncoding and coding data types (*A*–*C*) and taxon-specific sequences in 3′-UTRs (*D*). In (*A*), multiple tree inferences using distinct starting trees and subsequent refinement by nearest neighbor interchange (NNI) moves resulted in a better tree topology congruency (lower Robinson–Foulds distance) for 3′-UTR trees (UTR, 3′-UTRs of all species; UTR_393_, 3′-UTRs including only seven genomes of which no transcriptomes were available) as compared with trees calculated from similar amounts of coding sequence data (CDN, codons of all species; CDN_12_, codon positions 1 and 2 only, all species; AAS, amino acid sequence, all species); tree inference RAxML fast mode (-f E), model GTRCAT (or PROTCATJTTF) without or with NNI improvement under GTRGAMMA (PROTGAMMAJTTF) RAxML(-f J). In (*B*), we compared the rate of change of average per-site likelihood (blue) with the tree topology convergence (red; average Robinson–Foulds distances of ten trees), and the convergence of average trees from neighboring data points (green; Robinson–Foulds distance; e.g., average tree *n* compared with average tree *n* + 1,…). The rate of change of average per-site likelihood depends on the allowed-missing data in the alignments. The rate of change of average per-site likelihood can be computed fast (single inference per alignment) as compared with tree topology convergences (multiple inferences) and predicts an optimal number of allowed gaps per column in 3′-UTR multiple sequence alignments of about 100 missing species per pattern. (*C*) Influence of mixing 3′-UTR and CDS (coding sequences) on the resulting tree topology. Adding relatively small amounts of 3′-UTR to CDS had already a strong impact on the resulting tree topologies (red line), whereas adding small amounts of CDS to 3′-UTR had a much lower impact on the resulting tree (blue line). Note that both curves are different from the diagonal. (*D*) The 3′-UTRs of avian genes contain evolutionary signals that distinguish order- and family-level taxa. The similarity of the presence of transcription factor binding site motifs (TFBS) in 3′-UTRs of species decreases with increasing evolutionary distance between avian families. Shown are correlations (*Z* values) of the abundance of TFBS in 3′-UTRs of 97 randomly selected genes expressed in the passerine family Estrildidae versus Fringillidae, versus Basal Oscine families, versus family-level taxa of the order Charadriiformes, and the order Caprimulgiformes. The correlation of TFBS abundance between Charadriiformes and Caprimulgiformes (not shown) is *R*^2^=0.694. For the list of analyzed genes and species see supplementary table S3, Supplementary Material online.

**Fig. 2.**
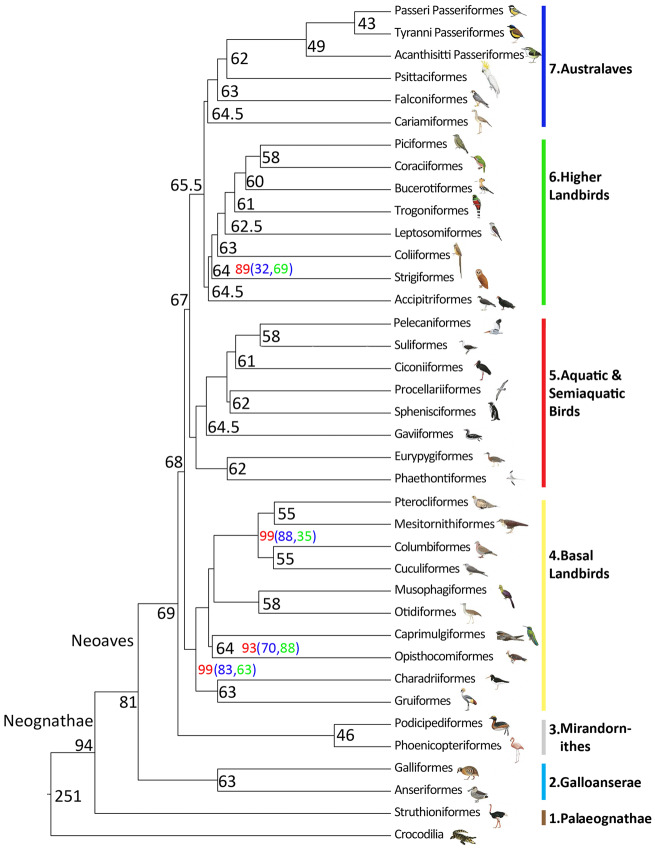
Order-level phylogeny of the birds resulting from the analysis of 3′-UTRs of 221 avian family-level taxa including 379 genera and 429 species (see fig. 3*A* and *B* for all families; supplementary fig. S6, Supplementary Material online for all species). In contrast to all previous phylogenies spanning the entire avian class, the statistical support values are high throughout, that is, the approximate likelihood-based measures of branch supports were maximal (SH-aLRT=100) in most cases, except for four branching points (red values). If we reduced the number of missing samples (gappiness) from 110 to 100, the support levels of these four branching points dropped (blue values), whereas all others remained maximal. In case of SH-aLRT values <100, we provide the support values from IQTREE2 ultrafast bootstrapping (green values). The tree is subdivided into seven higher-level clades, the Palaeognathae, the Galloanserae, the Mirandornithes, the Basal Landbirds, the Aquatic & Semiaquatic Birds, the Higher Landbirds, and the Australaves. Particular colors indicate each of the seven avian higher-level clades in all phylogenetic trees of the study. Thus, trivial names (Basal Landbirds, Higher Landbirds, Aquatic & Semiaquatic Birds) used in previous publications and in the current paper comprise different sets of bird order- and family-level taxa. Note that the hoatzin (Opisthocomiformes) resulted as the sister group of the Caprimulgiformes and that the flamingos (Phoenicopteriformes) and grebes (Podicipediformes) form the sister group Mirandornithes of all other Neoaves in our analysis. Black numbers at the nodes are the calculated divergence times of the order-level taxa in million years ago (Ma). Most of the extant order-level taxa evolved in the Paleocene, the other two during early Eocene and some lineages, likely, diverged already before the K-Pg 66 Ma boundary. For illustration purpose, the branch lengths are not scaled. Bird pictures are reproduced with permission of Lynx Edition.

Of these trees, the concatenated 3′-UTR alignment delivered the most stable tree topologies of the included species based upon repeated subsampling of molecular data (different cut-off levels of allowed-missing data in alignment columns) and repeated tree calculations using different starting trees (fig. 1*A* and *B*). 3′-UTR sequences either derived from trancriptomes and few genomes or derived only from transcriptomes resulted in a much higher congruency of repeated tree calculations with varying starting trees as compared with CDS (AAS, CODON, CODON12) sequences (fig. 1*A*). Besides separation of different sequence types (fig. 1*A* andsupplementary fig. S5, Supplementary Material online), we found that it is highly important to identify a suitable trade-off for allowed-missing data and total alignment length. This is especially true for transcriptome data sets in which some mRNAs were not detected or not expressed, so that not allowing gaps would result in losing most of the aligned data. For repeated tree inferences from alignments with distinct gappiness (defined as: filtering alignment patterns with a maximum allowed amount of gap characters=gap cut-off), tree convergence reached an optimum between 90 and 110 missing samples (fig. 1*B*, green line). Fewer fluctuations were observed in that gappiness range (fig. 1*B*, red line) when comparing the congruency of average trees of “neighboring alignments” (average tree [gap cut-off_*n*__−1_] vs. average tree [gap cut-off_*n*_]). Interestingly, the rate of change of average per site likelihood scores between distinct alignments predicted an optimal gappiness quite well (fig. 1*B*, blue line minimum at gap cut-off 100 [100 gappiness]), and it was computationally very efficient to calculate as it required just a single inference instead of multiple inferences per alignment. Thus, we used a gap cut-off (gappiness) of maximal 100 respective 110 gappiness for calculations of phylogenetic trees (see figs. 2, 3*A*, and 3*B*).

**Fig. 3.**
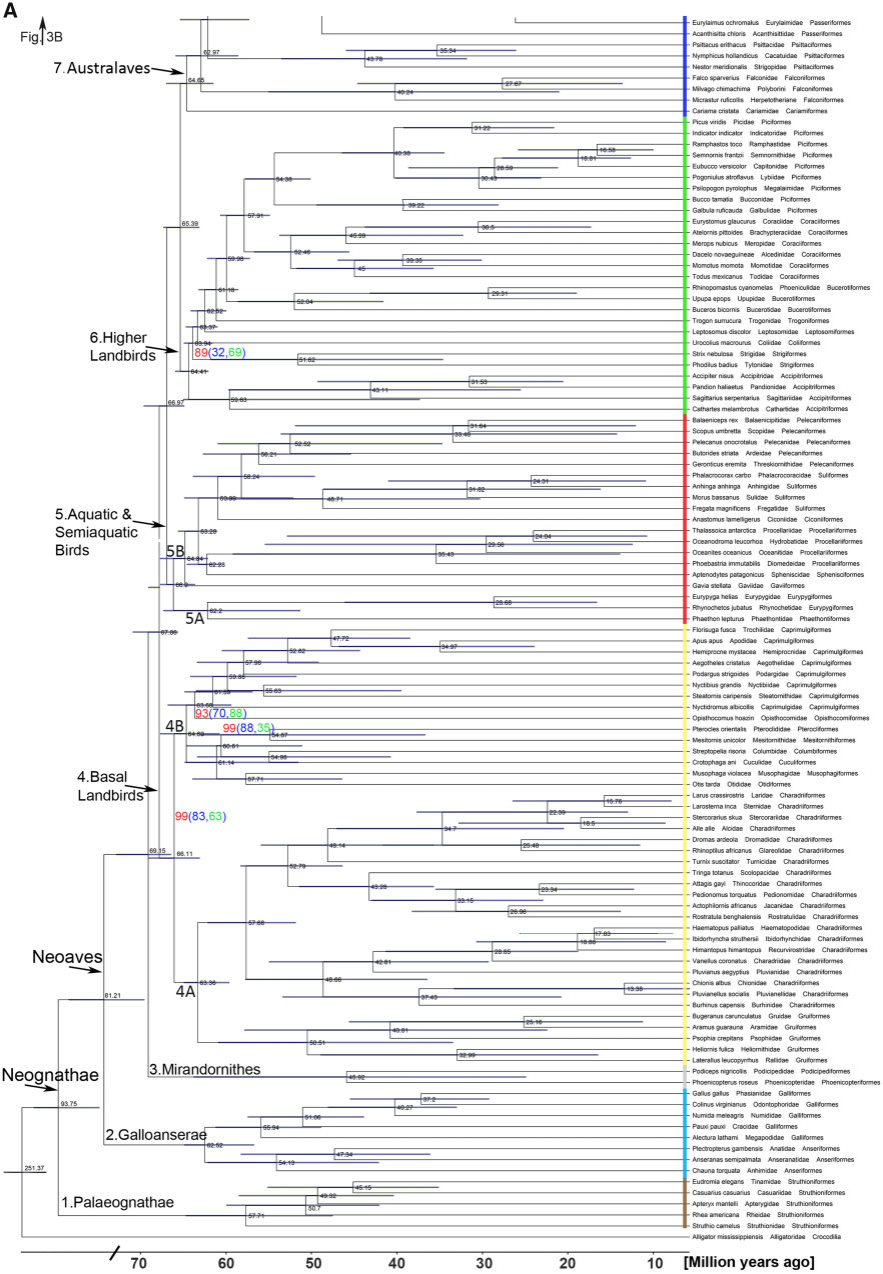
A family-level phylogeny of birds based on 3′-UTR sequences including all (106) nonpasserine (*A*) and most (115) passerine (*B*) family-level taxa. For simplicity, each of the families is represented by one species, listed as the species name, followed by the family name and the order name. In (*A*), the family-level taxa of the seven higher-level clades, the Palaeognathae, the Galloanserae, the Mirandornithes, the Basal Landbirds, the Aquatic & Semiaquatic Birds, the Higher Landbirds, and the Australaves are shown. The higher-level clades are color-coded as in figure 2. Of the Passeriformes (*B*), the suborders Acanthisitti (New Zealand wrens), Tyranni (suboscines), and Passeri (oscines or songbirds) are indicated and the Passeri is subdivided into ten oscine higher-level clades (OHCs). The tree was calculated by RAxML-ng using a large concatenated alignment of 3′-UTR residues as input (2,584,785 analyzable patterns, maximum 100 or 110 missing taxa [gappiness]). Approximate likelihood-based measures of branch support delivered maximal values (SH-aLRT=100) except those shown in red (for 110-gappiness) and blue (for 100-gappiness). SH-aLRT values are considered as quite conservative. In case of SH-aLRT values <100, we also provide support values from IQTREE2 ultrafast bootstrapping (UFBS, green values). In the few cases were SH-aLRT support was <80 (two for 110-gappiness; seven for 100-gappiness), the UFBS approach still reached good values of support in the range of 86–99. The timing of the branching points was calculated by DPPDiv. The entire tree including all 429 species is provided in supplementary figure S6, Supplementary Material online. Error bars are confidence intervals (95%). Time scale and divergence times are in million years ago. Diagonal bars indicate the part of the tree that is not scaled in order to reduce the size of the tree and the PDF.

**Fig. 3.**
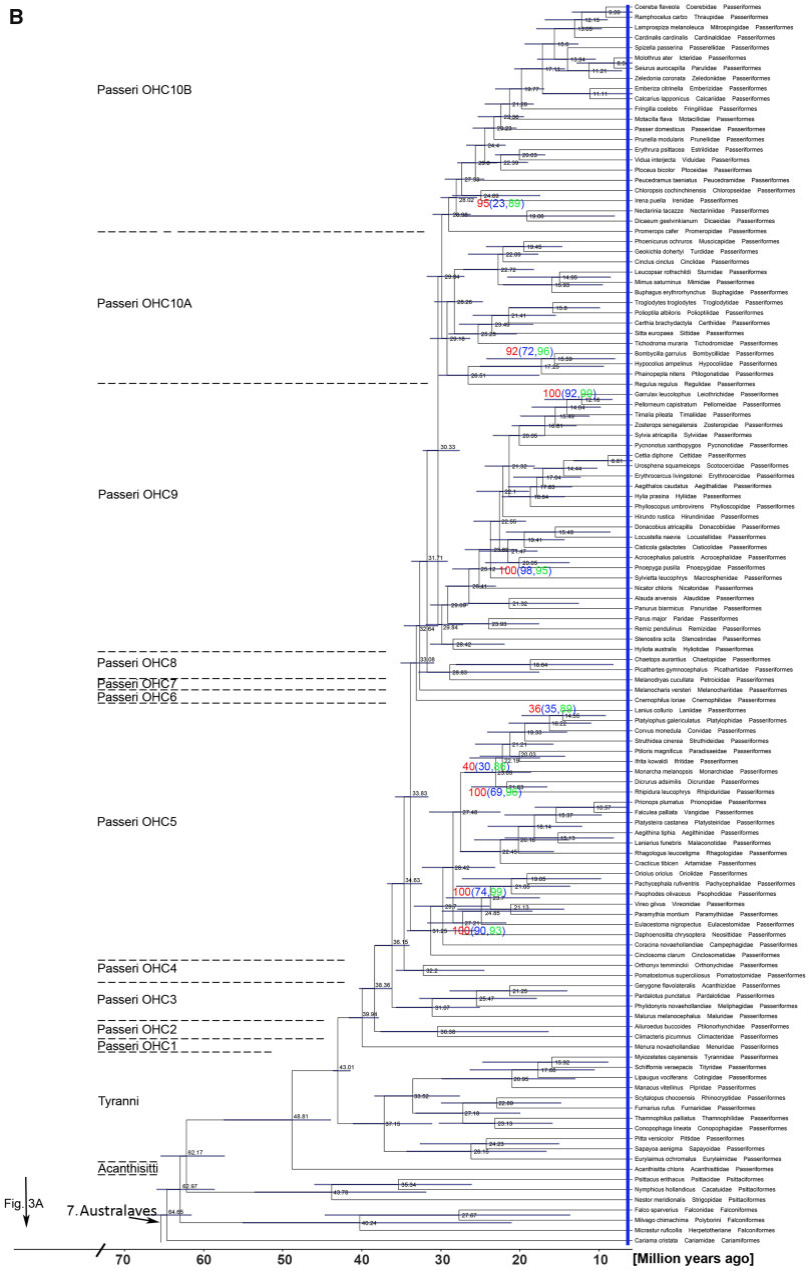
continued

In addition to tree inference from concatenated alignments, we also calculated the species tree using a coalescence approach (Mirarab et al. 2014) including up to 5,127 trees derived from gene-specific 3′-UTR sequences. The coalescent 3′-UTR-based avian species tree resulted in nine higher-level clades (supplementary fig. S4*D*, Supplementary Material online). The order-level and family-level relationships of both bioinformatic procedures converged for some clades, in particular, the Galloanserae and the Australaves (fig. 2 and supplementary fig. S4*D*, Supplementary Material online). However, other clades of the coalescent species tree, in particular, the relationships of family-level and order-level taxa comprised in the Mirandornithes (Sangster 2005), the Basal Landbirds, the Aquatic & Semiaquatic Birds of the concatenated tree (Clades 4–6 of fig. 2) were disarranged in ways (Clades 4–7 of supplementary fig. S4*D*, Supplementary Material online) that are not compatible with morphological and molecular evidence. We think that this is due to many very short 3′-UTR alignments for single genes, which fail to generate 3′-UTR gene trees of sufficient quality to be used in the coalescent approach. This shortcoming of the coalescent approach in combination with 3′-UTRs might be solved in future by statistical binning methods. From these data, we argue that a concatenation-based inference method is better suited than a coalescent approach to resolve the entire avian tree of life using 3′-UTR sequences. The coalescent approach with coding sequence data was not considered, as it did not deliver a comprehensive tree before (Jarvis et al. 2014).

In summary, by careful selection of sequence data type (3′-UTR) and content of missing data (gappiness), we found identical best log-likelihood scoring tree topologies, if using fast approximate or exhaustive tree inference methods and the concatenation method. The relatively conservative SH-aLRT branch support (values >80 are typically considered as high support) was maximal (SH-aLRT=100) for most of the branches (98.2% in case of 110 gappiness; 97.1% in case of 100 gappiness) and was fully supported by ultrafast bootstrapping (UFBS=100) for 93.6% of the branching points (fig. 3*A* and *B*). In the few cases were SH-aLRT support was <80 (*n* = 2 [0.5%] for 110 gappiness; *n* = 7 [1.7%] for 100 gappiness), UFBS still reached good values of support in the range of 86–99 (figs. 2, 3*A*, and 3*B*). Only the branch leading to the Strigiformes in the Higher Landbirds had weaker support values for both SH-aLRT (88 for 100 gappiness, 32 for 110 gappiness) and UFBS (32). Concerning well-established clades, such as the Charadriiformes and the Piciformes, our 3′-UTR family-level topologies agreed entirely or largely with previous studies that focused on these clades (Baker et al. 2007; Winkler 2015). At the lower taxonomic level, in each case, in which several species of a well-known family were sequenced, these species were correctly assigned to that family (e.g., Tinamidae, Trochilidae, Picidae, Paridae, Estrildidae, Fringillidae; supplementary fig. S6, Supplementary Material online). Similarly, species of the same genus were in each case correctly assigned to the expected genus in the 3′-UTR tree (e.g., genus *Podiceps* of the Podicipediformes, *Columba* of Columbiformes, *Charadrius* of Charadriiformes, *Falco* of Falconiformes, *Uraeginthus* and *Amadina* of Passeriformes; supplementary fig. S6, Supplementary Material online). In all these cases, statistical support was 100%.

We therefore conclude that, given sufficiently large data sets of noncoding 3′-UTR sequences in terms of number of taxa and alignment length (fig. 1*A* and *B*), RAxML’s fast approximate method enables computationally efficient phylogenomics, even for difficult-to-resolve phylogenies such as the avian tree of life (figs. 2, 3*A*, and 3*B*; supplementary fig. S6, Supplementary Material online).

### Comparison of the 3′-UTR-Based Tree and Trees Based on Coding Sequences

The relationships of many higher-level taxa in our 3′-UTR tree differed from those of the coding sequence trees (supplementary fig. S4*A*–*C*, Supplementary Material online). In particular, the coding trees resulted in unlikely relationships of certain higher-level taxa and did not support monophyly of several currently recognized higher-level taxa. For example, in the CODON tree, the Caprimulgiformes were split into distantly related subgroups, and parrots (Psittaciformes) were moved away from the Passeriformes, which resulted as the sister taxon of the mousebirds (Coliiformes) (supplementary fig. S4*A*, Supplementary Material online). In the CODON, CODON12, and the AAS trees, the Falconiformes and Cariamiformes were moved away from the Passeriformes and Psittaciformes to form an assemblage of birds of prey, embedded deeply in the phylogeny (fig. 2 and supplementary fig. S4*A* and *B*, Supplementary Material online). In the AAS tree, even the Strigiformes were enclosed in the birds of prey assemblage (supplementary fig. S4*C*, Supplementary Material online). By contrast, in all recent molecular approaches (Kimball et al. 2013; Jarvis et al. 2014; Prum et al. 2015) including our 3′-UTR tree (figs. 2 and 3*A*), Passeriformes, Psittaciformes, and Falconiformes are closely related and part of the taxon Australaves (Ericson 2012). This latter clade obtained strong support from a previous molecular phylogeny (Suh et al. 2011) and the sister group relationship of Psittaciformes and Passeriformes also conforms with paleontological data (Mayr G 2017). In the CODON12 and AAS tree, the Coliiformes were the closest relatives of the Passeriformes and Psittaciformes (supplementary fig. S4*B* and *C*, Supplementary Material online). Although there is some anatomical support for such a relationship (Berman and Raikow 1982), the Coliiformes were grouped with the Trogoniformes and other Higher Landbird clades in a phylogeny that was based on an analysis of a large number of morphological characters (Livezey and Zusi 2007). The type of sequences (noncoding vs. coding sequences) for tree construction had no impact on the relationships of higher-level taxa at the base of the trees, that is, on the basal position of Palaeognathae and Galloanserae (supplementary fig. S4, Supplementary Material online), even though noncoding and coding trees differed in the interrelationships of some taxa within the Palaeognathae (data not shown).

Mixed sequence data that contained defined amounts of coding and 3′-UTR sequences to analyze the influence of mixing 3′-UTR and CDS on the resulting tree topology resulted in topologies that are different from both pure CDS and pure 3′-UTR topologies (fig. 1*C*). Interestingly, adding relatively low amounts (e.g., 20%) of 3′-UTR to CDS had a strong impact on the resulting topologies, whereas adding low amounts of CDS (e.g., 20%) to the 3′-UTR had much lower impact on the resulting tree (fig. 1*C*). 3′-UTR sequences seem to contain a stronger phylogenetic signal than CDS. Thus, mixing the percentage of 3′-UTR and CDS in evolutionary models (Jarvis et al. 2014) is an arbitrary procedure, as there is no linear relation between the amount of either sequence type and the 100% model for 3′-UTRs and CDS, respectively (fig. 1*C*). The difference of our 3′-UTR tree as compared with trees derived from combined coding and noncoding sequence data is due to sequence type and taxa sampling (supplementary fig. S5, Supplementary Material online). These results agree with the conclusions drawn from a reanalysis of previous molecular phylogenies (Jarvis et al. 2014; Prum et al. 2015) by Reddy et al. (2017).

In summary, at the level of composition of higher taxa, the CODON tree (supplementary fig. S4*A*, Supplementary Material online), the CODON12 tree (supplementary fig. S4*B*, Supplementary Material online), and the AAS tree (supplementary fig. S4*C*, Supplementary Material online) differed considerably from the 3′-UTR tree (fig. 2) and from currently accepted relationships of avian higher-level taxa.

### The 3′-UTRs Contain Motifs Specific for Higher-Level and Lower-Level Clades

To identify signals specific to higher-level clades that are present in 3′-UTRs, we compared such sequences from the Caprimulgiformes, Charadriiformes, and selected subclades of the Passeriformes. The analysis of putative binding sites of RNA binding proteins and of micro RNAs did not show taxon-specific pattern. However, we found that the presence of putative transcription factor binding sites (TFBS) differed between higher-level clades, and between clades within the Passeriformes (fig. 1*D*): *Z*-score analysis of the abundance of TFBS in 3′-UTRs of 97 randomly selected transcribed genes showed high similarity between the closely related estrildid and fringillid songbird families (both in clade OHC10B), lower similarity between estrildid species and species of basal songbird families (in clade OHC1–OHC3), and even lower similarity with charadriiform and caprimulgiform species, as expected from their phylogenetic distance (fig. 1*D*; see Supplementary Material online for discussion of oscine higher-level clades [OHCs]).

Furthermore, we analyzed the pattern of TFBS in detail within family-level taxa of which we had multiple species belonging to at least three genera. As an example, we present the pattern of TFBS in the 3′-UTR of the gene EMC1 of the Spheniscidae, the penguins (supplementary fig. S7, Supplementary Material online). The presence of TFBS in that 3′-UTR shows a family-specific signature (supplementary fig. S7*A*, Supplementary Material online) as well genus-specific signatures for each of the three included genera, that is, *Aptenodytes* (supplementary fig. S7*B*, Supplementary Material online), *Eudyptes* (supplementary fig. S7*C*, Supplementary Material online), and *Pygoscelis* (supplementary fig. S7*D*, Supplementary Material online). The combinatorial pattern of the TFBS distinguished the genera. Comparable results were obtained for songbird families (Estrildidae, Fringillidae) of which we had multiple species of three genera (data not shown). Obviously, a complete representation of all 19 penguin species would be desirable to further solidify that result. Although we did not analyze the TFBS in the 3′-UTRs of all taxa included in our study, the analysis nevertheless suggests that the presence of the TFBS is taxon-specific at the order, family, and genus level. These taxon-specific sequences, likely, represent the evolutionary signals extracted by our bioinformatic procedure used to construct the 3′-UTR avian tree of life.

### The Higher-Level (Order-Level) Avian Tree of Life

The 3′-UTR-based tree of life resolved the relationship of all avian orders including the Opisthocomiformes (hoatzins) with good statistical support (fig. 2). In that phylogeny, extant birds fall into seven major clades (fig. 2). Clade 1 represents the Palaeognathae and Clades 2–7 encompass the Neognathae, which are subdivided into the Galloanserae (landfowl and waterfowl; Clade 2) and the Neoaves (Clades 3–7) (fig. 2). Among the Neoaves, Clade 3 includes the Mirandornithes, the flamingos, and grebes, Clade 4 represents the “Basal Landbirds,” Clade 5 encompasses the “Aquatic and Semiaquatic Birds,” Clade 6 is the “Higher Landbird Clade,” and Clade 7 represents the Australaves (Ericson 2012; Kimball et al. 2013) (fig. 2). Four of the 35 order-level relationships were sensitive to the amount of data: if we decreased the gappiness from 110 to 100 missing samples, the statistical support values of this four branching points dropped from {99%, 99%, 93%, 89%} support to {88%, 32%, 88%, 83%} support, but stayed 100% for all other branching points (fig. 2). In particular, the relationship of the Strigiformes (SH-aLRT: 88, respectively, 32; UFBS: 69) requires further attention.

It should be noted that the composition and interrelationships of Clades 3–6 differ substantially from previous phylogenies (Hackett et al. 2008; Jetz et al. 2012; Jarvis et al. 2014; Prum et al. 2015) as discussed below for family-level taxa. Thus, trivial names (Basal Landbirds, Higher Landbirds, Aquatic & Semiaquatic Birds) used in previous publications and in the current paper comprise different sets of bird order- and family-level taxa. Nevertheless, the interfamilial relationships within some higher-level subclades were similar between the present study and previous reports (Jarvis et al. 2014; Prum et al. 2015) (see below).

In the Neoaves (Clades 3–7; fig. 3*A*), the Mirandornithes (Clade 3; fig. 3*A*), are the sister taxon of all other taxa, which is in contrast to all previous molecular trees (Ericson et al. 2006; Hackett et al. 2008; Jarvis et al. 2014; Prum et al. 2015; Kimball et al. 2019). In previous works, either a clade composed of Mirandornithes, Pterocliformes, Mesitornithiformes, and Columbiformes (Jarvis et al. 2014) or the Charadriiformes (Prum et al. 2015) were suggested to be the sister group of all other Neoaves. A close relationship between Phoenicopteriformes (flamingos) and the Podicipediformes (grebes) is also supported by morphological data (Mayr 2004).

### The Avian Family-Level Tree of Life

We included all 106 currently recognized nonpasserine families and 90% (115) of the passerine family-level clades, which significantly increased the taxon sampling compared with previous comprehensive phylogenies (Hackett et al. 2008: 93 nonpasserine, 24 passerine family-level clades; Jarvis et al. 2014: 39 nonpasserine, two passerine family-level clades; Prum et al. 2015: 91 nonpasserine, 31 passerine family-level clades; Oliveros et al. 2019: five nonpasserine, 125 passerine family-level clades). As adding families impacts the entire tree, a phylogeny missing many family-level taxa is unlikely to maintain its higher-level topology, if further families were included in the phylogenetic analysis. Due to the low amount of sequence data included in the species-rich study of Hackett et al. (2008) and due to the low number of families in the sequence-rich study of Jarvis et al. (2014), we restrict the following comparisons mainly to the Prum et al. (2015) study for nonpasserines and to the Oliveros et al. (2019) phylogeny for passerines. For more detailed considerations of the interfamilial relationships than those presented below, we refer to the supplementary discussion, Supplementary Material online.

Within the Palaeognathae (Clade 1) and the Galloanserae (Clade 2), the interrelationships of the family-level taxa (fig. 3*A*) agree with the phylogeny of Prum et al. (2015). Interestingly, among the Palaeognathae, these relationships differ from those reported on the basis of conserved noncoding elements and a coalescent inference procedure, in which rheas are the sister group of cassowaries, emus, and kiwis (Sackton et al. 2019). The differences are due to the tree inference procedure (see Supplementary Material online). Furthermore, we confirm the interrelationships of the seabird subclade of the waterbird group of Prum et al. (2015), here informally named Aquatic & Semiaquatic Birds (Clade 5), but not its sister group relationship to the Caprimulgiformes and Mirandornithes (fig. 3*A*).

A major difference to the higher-level clades recognized before (Prum et al. 2015) concerns the Clade 4 (fig. 3*A*) of our tree, the Basal Landbirds, which comprises two subclades. One of these (fig. 3*A*, Clade 4A) includes Charadriiformes (shorebirds and allies) and Gruiformes (cranes and allies), whereas the other subclade (fig. 3*A*, Clade 4B) unites the Musophagiformes (turacos), Otidiformes (bustards), Mesitornithiformes (mesites), Pterocliformes (sandgrouse), Columbiformes (doves), and Cuculiformes (cuckoos) on the one hand, and Opisthocomiformes (hoatzins) and Caprimulgiformes on the other. In the phylogeny of Prum et al. (2015), by contrast, the early-diverging landbirds were subdivided in three higher-level clades and Charadriiformes resulted in a clade that also contained the aquatic and semiaquatic birds. Furthermore, the interrelationships of Columbiformes, Cuculiformes, Otidiformes, Musophagiformes, Mesitornithiformes, and Pterocliformes within the Basal Landbirds differ from previous works (Hackett et al. 2008; Prum et al. 2015). In further contrast to the results of Prum et al. (2015), the Caprimulgiformes are not the sister group of all other Neoaves, but are the sister group of the Opisthocomiformes (hoatzin) in our study. Previous works suggested closest relationship of the hoatzin with varying groups, such as Pelecaniformes (Hackett et al. 2008), Gruiformes, and Charadriiformes (Jarvis et al. 2014), or placed it in an isolated clade somewhere between aquatic and semiaquatic birds and birds of prey (Prum et al. 2015).

The interrelationships of the taxa of our Higher Landbirds (Clade 6) agree with the tree topology of Prum et al. (2015) and correspond to the Afroaves group of Jarvis et al. (2014)—although the latter study missed many of the included families. Clade 7 (fig. 3*A*) consists of the Australaves (Hackett et al. 2008; Suh et al. 2011) that include the seriemas (Cariamiformes), the falcons (Falconiformes), the parrots (Psittaciformes), and the passerines (Passeriformes), which is also consistent with previous phylogenetic results (Hackett et al. 2008; Suh et al. 2011; Jarvis et al. 2014; Prum et al. 2015).

In the Passeriformes, the 92–103 studied (the count depends on the classification of IOU or HBW, see Materials and Methods) family-level taxa of songbirds (Oscines) fall into ten higher-level clades (fig. 3*B*, supplementary fig. S6, Supplementary Material online, and see Supplementary Material online). The earliest divergence (fig. 3) is represented by the lyrebirds (Menuridae) and the evolutionarily youngest clades include most taxa previously summarized as the Passerida (Sibley and Ahlquist 1991; Barker et al. 2004). The present passerine phylogeny differs widely from all previous ones in the composition and interrelationships of the OHCs (fig. 3) (Barker et al. 2004; Aggerbeck et al. 2014), with the exception of the recent study of Oliveros et al. (2019), which was based on ultraconserved molecular elements. As the Oliveros-tree of oscine family-level taxa (Oliveros et al. 2019) and that part of our tree are very similar, these trees might converge on the true phylogeny of songbird families. The minor differences between the passerine part of the present tree and the Oliveros-tree might be due to the use of different genera. Despite low SH-aLRT support values, the corvid clades had high UFBS support (85–99) (fig. 3*B*); the future addition of further genera, in particular of the corvid lineages, might solve the ambiguities between the Oliveros-tree and the present tree. The strong differences to other passerine trees (Barker et al. 2004; Jetz et al. 2012; Aggerbeck et al. 2014; Claramunt and Cracraft 2015) are likely due to their lower sampling of families, the overall number of sequences analyzed, and/or the use of mitochondrial and nuclear coding sequences. Further details of interfamilial relationships of oscine families are discussed in Supplementary Material online. Here, we just mention that our data support the removal of the taxon *Hylia* from the Scotocercidae into its own family-level taxon Hyliidae (Bates 1930; Fregin et al. 2012) and that the split of Erythrocercidae, Scotocercidae, and Cettiidae altogether needs reconsideration (supplementary figs. S3 and S6, Supplementary Material online and see Supplementary Material online).

### Time Calibration of the Family-Level Phylogeny

We used DPPDiv (Heath et al. 2012) for time calibration of our family-level phylogeny. DPPDiv uses the dirichlet process prior (DPP) model or the uncorrelated gamma-distributed rates (UGR) model. Although these two models yielded broadly congruent divergence dates for many clades (difference between models: 2.4 ± 4.7 My [mean ± SD]) (Million years), they show various differences in detail and none of the results is entirely congruent with the fossil record (supplementary table S4, Supplementary Material online). In general, the UGR model (figs. 2 and 3) led to divergence times of families that showed less conflict with time-calibrated fossil data as compared with the use of the DPP model. For example, the estimated divergence time of Galliformes and Anseriformes of 62.5 Ma (million years ago) fits well with a recently reported Mesozoic fossil (66.7 Ma) that is close to the last common ancestor of Galloanserae (Field et al. 2020) (supplementary table S4, Supplementary Material online). Furthermore, for phasianine and odontophorine Galliformes, the calculated divergence time is 37 Ma, whereas the earliest record of a galliform belonging to the clade Odontophorinae+Phasianinae, the taxon *Palaeortyx*, stems from the early Oligocene, some 32 Ma (Mayr G 2017; Zelenkov 2019). The divergence dates of the UGR model also conform with the fossil record of crown group Procellariiformes, Gruiformes, and Accipitriformes, with fossils of the procellariiform Diomedeidae, the gruiform Rallidae, and the accipitriform Pandioninae having been described from the early Oligocene, some 32–34 Ma (Mayr 2009; Mayr G 2017). For Mirandornithes, by contrast, the calculated divergence date of 46 Ma for Podicipediformes and Phoenicopteriformes distinctly predates their earliest known fossils, the earliest fossil Podicipediformes being from the late Oligocene/earliest Miocene (∼20 Ma), and the earliest Phoenicopteriformes being from the early Oligocene (32 Ma; Mayr G 2017) (supplementary table S4, Supplementary Material online). This suggests substantial ghost lineages for both Podicipediformes and Phoenicopteriformes. However, most calculated branching points come with large confidence intervals (fig. 3*A* and supplementary fig. S6, Supplementary Material online). Nevertheless, the overall disparity of fossil and molecular age determinations is rather low, being between 9 and 11 My, if all fossil data are considered (supplementary table S4, Supplementary Material online). These discrepancies are likely due to either 1) the limited fossil record of certain clades (Mayr G 2017), 2) the existence of clades with a single extant species, which does not allow molecular dating of the diversification of the crown group (Van Tuinen et al. 2006), and 3) the limited species sampling and large confidence intervals of some molecular age determinations.

**Fig. 4.**
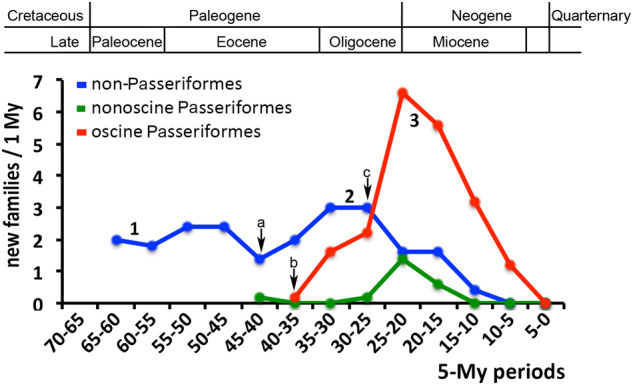
The diversification of oscine passerine families (red) contrasts with that of suboscine passerine families (green) and of nonpasserine families (blue) after the early Miocene epoch. The numbers of new family-level taxa per million year (My) were calculated from the family-level phylogeny according to intervals of 5 My. After the K-Pg boundary (66 Ma), during the Paleocene and early Eocene most neognath order-level taxa emerged with a rather steady rate of new family-level taxa per My (“1”). During the Oligocene epoch, a major diversification event occurred (“2”), which concerned both nonpasserine and passerine family-level taxa (50 families of 12 orders), the highest diversification rate of new family-level clades (3.0 nonpasserine and 2.0 passerine family-level clades/My) taking place between 35 and 25 Ma during the Rupelian and Chattian stages. A third major diversification event (“3”) concerned mainly passerine family-level taxa, having a peak 25–15 Ma in the Aquitanian and Burdigalian stages of the early Miocene (1.6 nonpasserine, 7.1 passerine families/My). Since the Miocene, the radiation of oscine family-level taxa contrasts negatively with diversification rates of nonoscine passerine (New Zealand wrens and suboscines) and nonpasserine families. Arrows indicate the calculated emergence of family-level taxa that evolved vocal learning, the parrots (a), the passerines (b), and the hummingbirds (c). The divergence times of family-level clades were calculated with DPPDiv applying the uncorrected gamma-distributed rate model (see fig. 3*A* and *B*;supplementary fig. S6, Supplementary Material online).

The time-calibrated phylogeny (figs. 2 and 3*A* and *B*;supplementary fig. S6, Supplementary Material online) shows divergence dates for Palaeognathae and Neognathae (94 Ma) and Galloanserae and Neoaves (81 Ma) that are much earlier than those suggested previously (Prum et al. 2015). The divergence dates of the Mirandornithes and those of the Basal Landbirds and of the Aquatic & Semiaquatic Bird lineages precede the K-Pg boundary (fig. 2). The initial divergences within many other neoavian lineages occurred ∼10 My after the K-Pg boundary, in the Eocene. Although an early Cenozoic neoavian radiation is strongly supported by fossil data (Mayr 2014; Ksepka et al. 2017; Mayr G 2017), the confidence intervals of our molecular divergence times (fig. 3*A*) do not exclude the possibility that some Neoaves lineages evolved before the K-Pg mass extinction as suggested before in molecular phylogenies (Suh et al. 2015; Houde et al. 2020). This is also suggested by the occurrence in the earliest Cenozoic of stem group representatives of various only distantly related and deeply neoavian taxa, such as penguins and owls (Mayr 2014). During the Oligocene epoch, a second major diversification event occurred, which concerned both nonpasserine and passerine families (50 families of 12 orders), the highest diversification rate of new family-level clades (3.0 for nonpasserine and 2.0 for passerine family-level clades per million years) taking place between 35 and 25 Ma during the Rupelian and Chattian stages of the Oligocene (fig. 4). This pattern of divergence times is reminiscent of that previously suggested by Van Tuinen et al. (2006).

The third major diversification event concerned mainly passerine family-level clades, having a peak 25–15 Ma in the Aquitanian and Burdigalian stages of the early Miocene (1.6 for nonpasserine, 7.1 for passerine families per million years) (fig. 4). This passerine diversification during the early Miocene (71 new family-level passerine clades; for diversification times, see fig. 3 and supplementary fig. S6, Supplementary Material online) contrasts with the diversification of nonpasserine taxa (16 new family-level clades; for diversification times, see fig. 3 and supplementary fig. S6, Supplementary Material online) (fig. 4). Since the middle Miocene (15 Ma), 22 extant passerine family-level clades (all of the oscine passerines), but only two extant nonpasserine family-level clades, both of the Charadriiformes, evolved. The diversification times of oscine family-level taxa agree with those estimated by Oliveros et al. (2019), but are much later than those calculated in previous studies using coding sequences (Ericson et al. 2014). Thus, although our phylogeny includes only passerines clades that survived until today, there may have been a strong negative impact of the passerine radiation on the evolution of new clades in most other higher-level (ordinal) bird taxa. Alternatively, nonpasserine family-level taxa might have radiated earlier than the oscines families and achieved optimal family-level diversity before the Miocene. Remarkably, however, family-level taxa that underwent speciation during the last 10 My are as common among oscine passerines as they are among nonpasserines: Of family-level taxa that were represented with more than one species in our study, 54% (22 of 41) of the nonpasserine and 58% (21 of 36) of the passerine clades underwent considerable diversification (supplementary fig. S6, Supplementary Material online).

The overall reduced number of family-level taxa during the last 10 My (figs. 3 and 4) may be due to the fact that this comparatively short time interval did not allow for the accumulation of much morphological disparity; many family-level clades that evolved during that interval were distinguished based on molecular differences.

## Discussion

By including representatives of all nonpasserine families and most passerine families, we show that 1) the molecular tree based on 3′-UTRs is, in bioinformatical terms, the most stable tree as compared with trees computed from coding sequences; and 2) the 3′-UTR tree resolves the higher-level relationships of all included taxa without any ad hoc assumptions such as the selection of certain genes (Prum et al. 2015), or the arbitrary combination of coding and noncoding sequences (Jarvis et al. 2014). 3) The tree-building capacity of 3′-UTRs reflects a strong phylogenetic signal, which might be related to the presence of TFBS motifs in the 3′-UTRs. 4) Our phylogeny suggests that the avian tree of life can be resolved using a moderate amount of sequencing data derived from transcriptomes. This avoids specialist knowledge for assembling entire genomes as well as high bioinformatics costs of comparing large numbers of large genomes. 5) The resulting 3′-UTR-tree shows a well-resolved topology including all avian order-level taxa, while dividing the Neoaves into five major clades that differ from previous phylogenies. 6) The Mirandornithes (flamingos and grebes) are the sister group of all Neoaves, and the hoatzin, a previous phylogenetic enigma, is shown to be closely related to the Caprimulgiformes. 7) The negative correlation in the temporal diversification of passerine and nonpasserine family-level clades might be due to the vocal learning capacity of oscine passerines (see fig. 4).

### Are 3′-UTRs Ideal for Molecular Tree Building?

The increased length and the evolution of alternative 3′-UTRs, as they are observed in vertebrates, the amount and type of TFBS, as well as protein binding sites are expected to increase the complexity of species-specific tissue-specific gene expression regulation (Sandberg et al. 2008; Lianoglou et al. 2013; Cohen et al. 2014; Mayr G 2017; Lee and Mayr 2019; Xu et al. 2019). Here, we demonstrate that 3′-UTR-based molecular trees resolve the avian tree of life with good statistical support throughout. On one hand, the taxon-specific presence of TFBS in 3′-UTRs might just be seen as an indicator of conserved sequences with yet unknown function. On the other hand, there are increasing observations of a functional role of transcription factor binding to 3′-UTRs for transcriptional and posttranscriptional processes. Regarding the transcriptional role, a simultaneous binding of transcription factors to 5′-UTR and 3′-UTR has been shown (Jash et al. 2012; Tan-Wong et al. 2012; Sun et al. 2018). This suggests that transcription factors may mediate intra- and intermolecular loop interactions bringing structurally together promoter and terminator, which would ensure the RNA polymerase to reload on the promoter efficiently (Jash et al. 2012; Tan-Wong et al. 2012; Sun et al. 2018). Furthermore, transcription factors bind to RNA; for example, Wilm’s tumor 1 regulates RNAs through binding to the 3′-UTR (Bharathavikru et al. 2017) and binding of SOX9 to 3′-UTR was associated with posttranscriptional processes (Penrad-Mobayed et al. 2018).

Tuğrul et al. (2015) used a biophysical model for directional selection on gene expression to estimate the rates of gain and loss of TFBSs. They showed that multiple TFBSs can evolve simultaneously allowing a biophysical cooperativity between transcription factors, and that the presence of pre-sites for transcription factor binding would facilitate the gain of TFBS. Here, we show the first evidence that links the enrichment of TFBS or TF pre-sites to systematic differences between taxa. Thus, even if there is little difference in the repertoire of protein-coding genes between species, the evolutionary divergence of 3′-UTRs is suggested to be an exceptionally important mechanism for rapid evolution, such as the speciation of cichlid fishes, through increased regulatory complexity of area-specific gene expression (Xiong et al. 2018). Such a scenario might be present as well in birds. In relation to this, most genes thought to be songbird-specific (Lovell et al. 2014) or parrot-specific (Wirthlin et al. 2018) are detectable in improved genomes by including extensive transcriptome data (Yin et al. 2019). Thus, in contrast to the common repertoire of avian genes, our data show strong differences in 3′-UTR sequences between avian orders, between families within orders, and between genera of a family (fig. 1*D*). As these sequence differences concern the presence of TFBS motifs, 3′-UTRs contain, potentially, an evolutionary signal of speciation.

Whether the resolution of the avian–level tree of life is due to particular features of 3′-UTRs and their potential importance for avian speciation, or whether it might also be achieved with other types of noncoding sequences, is open for discussion. Due to the short length of the 5′-UTR and to the nature of transcription, the number of 5′-UTR and of intronic sequences in our data were too few to allow testing of whether these sequences also contain enough evolutionary signal to properly resolve the tree avian tree of life. Ultraconserved elements appear to yield a well-resolved phylogenetic tree of passerine family-level taxa (Oliveros et al. 2019), very similar to the passerine part of our tree. However, it should be noted that the coding sequences, too, delivered a passerine part of the avian tree that had many similarities with the 3′-UTR tree despite the strong differences concerning the interrelationships of nonpasserine orders (data not shown). Furthermore, there are certain differences between Oliveros-tree (Oliveros et al. 2019) and the passerine part of our 3′-UTR tree, which might be due to the taxa used or the different data types. Thus, we need to see, whether coding sequences, ultraconserved elements, and 3′-UTRs converge on the same phylogenetic tree of passerine families in case that enough taxa and sequences are included in the tree calculation. Retroposons are another type of sequence that might resolve the avian phylogeny. However, the techniques involved in their study require very large genomic data sets. Even though analyses of retroposon insertions of bird genomes provided important new insights (Suh et al. 2011), they could not fully resolve the avian phylogenetic tree (Suh et al. 2015).

Interestingly, there are many similarities between nuclear sequence, or mitochondrial sequence-derived phylogenetic trees and the noncoding sequence-derived tree within certain orders and within families, whereas nuclear and mitochondrial data types seem to fail for data sets spanning many orders (Pacheco et al. 2011; Jarvis et al. 2014; Prum et al. 2015). Thus, multiple types of molecular sequences including mitochondrial sequences or nuclear sequences might resolve taxon relationships locally (e.g., within families), whereas the global resolution of the avian tree of life might require particular noncoding sequences such as 3′-UTRs (this study) and ultraconserved elements (Oliveros et al. 2019).

### Why Are Trees Based on 3′-UTR Sequences Different from Those Based on Coding Sequences?

Molecular phylogenetic trees based on bioinformatics tools clearly deliver different trees based on the function of the sequences used, as has been demonstrated before (Jarvis et al. 2014; Reddy et al. 2017). Trees based on coding sequences (CODON, CODON12, AAS) and noncoding-based trees (3′-UTR) are expected to be different due to species-specific selection pressures that favor removal of single nucleotide mutations of coding DNA as compared with noncoding sequences. In contrast, mutations in the 3′-UTR would only affect certain regulatory elements and in consequence might affect gene expression in some tissues (Mayr 2016; Mayr C 2017), but would unlikely stop protein expression body-wide, as might occur in case of mutations in the coding sequences. Thus, similar selection pressures due to similar environmental conditions might favor convergent developments in protein-coding sequences of distantly related species. Such an example might concern the birds of prey that were grouped together in one assemblage in the coding sequence trees (supplementary fig. S4, Supplementary Material online). However, in the case of vocal learning, another rare avian phenotype, which is present in the hummingbirds, parrots, and passerines (Gahr 2000; Jarvis et al. 2000), these three taxa were not grouped together in the coding trees (supplementary fig. S4, Supplementary Material online).

### Molecular Sequences and Anatomy-Based Trees

Concerning some clades, molecular phylogenies, in particular, those spanning the entire class of birds (e.g., this study; Jarvis et al. 2014; Prum et al. 2015), are substantially different from phylogenies derived from anatomical data (Livezey and Zusi 2007). As the present phylogeny includes all nonpasserine families, the differences between anatomical and molecular trees are unlikely to be due to different taxonomic samplings but due to the type of data analyzed. Clearly, the relationships of certain higher-level clades in the molecular phylogenies, such as the relationship of tropicbirds, kagus, and sunbitterns and their proximity to the Aquatic & Semiaquatic Birds, are unexpected and ask for new ontogenetic and morphological studies in order to assess the anatomical plausibility of these findings. In some cases, other surprising clades derived from analyses of molecular data have already been confirmed by morphological data, such as the sister group relationship between the morphologically and behaviorally very disparate grebes and flamingos (Van Tuinen et al. 2001; Mayr 2004). More recently, it was also hypothesized that the plesiomorphic presence of a large lacrimal bone may support the basal position of the Caprimulgidae within Caprimulgiformes, with this bone being reduced in other Caprimulgiformes (Chen et al. 2019; contra Mayr 2010). Much future anatomical work is, however, needed for an improved integrative understanding of avian phylogeny beyond insights derived from molecular sequence data.

### Implications of the Evolution of Vocal Learning for the Avian Tree of Life

The peaks of family-level diversification during the evolution of birds may have been caused by drastic changes of macroecological niches due to events such as global cooling, the related drop in sea levels and thus increased connectivity between landmasses and reduced CO_2_ levels that favored the spread of grassland or the desiccation of landmasses (Zachos et al. 2001). Opposite scenarios of macroecological changes exist for global warming (Zachos et al. 2001). There are, however, no clear catastrophic or macroecological events except for the progressing global cooling that parallels the massive passerine radiation in the late Oligocene and the Miocene (Hansen et al. 2013). A recent paper studying the evolution of the Passeriformes suggested that more complex mechanisms than temperature change or vacant ecological niches are responsible for passerine radiation events (Oliveros et al. 2019). Whatever the scenarios may have been, the significant radiation of oscine passerine family-level taxa since the Miocene strongly contrasts with the subdued diversification of new nonpasserine clades among most arboreal birds and of suboscine passerine family-level taxa (figs. 3*B* and 4).

The hallmark of songbirds is their singing behavior, which is important for mate choice and territorial defense. A key feature of songbird singing behavior is that the songs are learned (Goller and Shizuka 2018). Thus, we discuss, whether the evolution of vocal learning contributes to the extraordinary success of songbirds (comprising about half of all avian family-level taxa and species [∼4,500 species]) and the attenuation of the evolution of nonpasserine families in the last 20 My. Vocal production learning of males occurs in songbird families (suborder oscines of the Passeriformes), parrot families (Psittaciformes) and in the hummingbird family (Trochilidae of the Caprimulgiformes) (Gahr 2000; Jarvis et al. 2000; Cruickshank et al. 2008; Baptista and Schuchmann 2010). The statistical analysis of the distribution of avian families that utter learned vocalizations showed that vocal production learning evolved three times independently (supplementary fig. S8, Supplementary Material online), as hypothesized previously (Jarvis et al. 2014). The alternative scenario, that vocal learning evolved twice in the hummingbirds and in the common ancestor of parrots and passerines and was lost twice in the New Zealand wrens and suboscine passerines, is not favored by the statistical models (supplementary fig. S8, Supplementary Material online).

Among all family-level taxa of songbirds, the songs of males are generally assumed to be learned whereas short vocalizations, the calls, are generally thought to be innate (Vicario 2004). If we combine data from the wild (mainly based on anecdotal observations and the observation of vocal dialects) and from laboratory conditions, species of 70 families show vocal production learning (supplementary table S5, Supplementary Material online). Whether vocal learning occurs in all songbird families needs to be assessed, as calls might be also learned in some species (Zann 1990) and the distinction between songs and calls is a species-specific problem. This is particularly relevant for about 20 family-level taxa that are made up of only a few species of which the vocalizations are not well-known, such as the Rhagologidae or Scotocercidae. Nevertheless, even if we assumed that all oscine family-level taxa, which are currently not known to exhibit vocal learning indeed lack this capacity, the statistical analysis supported the hypothesis that vocal learning evolved just once among the passerines with the emergence of the oscines (supplementary fig. S8, Supplementary Material online). In connection with this ancestral origin of song learning among oscines, males of both the phylogenetically basal lyrebirds and scrubbirds do learn or are even superb song imitators (Armstrong 1963; Robinson and Curtis 1996).

Vocal (song and call) production learning of songbirds requires the development of the so-called song control system (Nottebohm et al. 1976), a neural circuit that orchestrates the movements of the syrinx. The ancestral status of vocal learning suggests the presence of the song control system in all songbird families. Indeed, the anatomical identification of parts of the song control system in species of 43 songbird family-level taxa including fairywrens and gerygones (OHC3) at the base of and true finches (OHC10) at the top of oscine diversification suggests the ancestral evolution of the song control system among oscines (supplementary table S6, Supplementary Material online). Because we found the song system in the brain of each songbird species that we could study (supplementary table S6, Supplementary Material online; Gahr M, unpublished data), we also expect to find it in lyrebirds and scrubbirds, at the base of the songbird clade. Whether the evolution of precursors of the oscine forebrain song control system had already occurred in subocines (Liu et al. 2013) or whether some suboscine genera convergently developed forebrain song control areas requires further comparative study.

The negative correlation between the evolution of new songbird families and those of new suboscine passerine and nonpasserine families during the last 20 My suggests a faster speciation and exploitation of ecological niches of oscine species. This might be due to vocal learning that is ancestral in oscine passerines (supplementary fig. S8, Supplementary Material online). A comparison of oscine and suboscine South-American taxa showed that evolutionary bursts in rates of speciation and song evolution coincide (Mason et al. 2017). However, overall rates of vocal evolution are higher among taxa with learned songs as compared with taxa with innate songs (Mason et al. 2017). Furthermore, sexual selection of song promotes the capacity of adult song learning (Robinson et al. 2019). As mate choice and territoriality are highly dependent on vocal displays in songbirds, vocal production learning is likely a key invention for fast speciation and the macroevolutionary pattern of species richness of the oscines. Vicariance and dispersal, too, likely play a major role in the evolution of Neotropical avifauna (Smith et al. 2014). Therefore, we suggest that the combination of vocal learning and dispersal behaviors of songbirds allowed songbirds to attenuate the evolution of nonvocal learning taxa competing for the same ecological niches ever since songbirds emigrated out of Australia in the Miocene some 20 Ma (Claramunt and Cracraft 2015; Oliveros et al. 2019).

In summary, we suggest that the 3′-UTRs contain significant evolutionary signals that result in true relationships, if used in unbiased phylogenetic tree solving procedures. Therefore, as we included all nonpasserine family-level taxa, the presented phylogenetic tree shows the relationship of all these taxa, that is, of all avian orders. The evolutionary timing of the divergences of family-level taxa suggests that the strong radiation of oscine passerine family-level taxa attenuated the evolution of new forms of nonpasserine taxa in the last 20 My, a process that might involve the evolution of the vocal learning behavior of songbirds.

## Materials and Methods

### Species and Tissue Samples

We produced whole transcriptomes of various tissues, preferentially brain or blood samples (supplementary table S1, Supplementary Material online). If possible, brain tissue was used to provide the most complex transcriptome libraries in terms of number of expressed transcripts. However, for animal protection reasons, in many cases, we used small blood samples or cryopreserved museum specimens (mainly liver or muscle) that were kindly provided by various collections (supplementary table S1, Supplementary Material online).

Different taxonomists recognize somewhat different genera as discrete family-level taxa. Thus, in 2017, the HBW recognized 243 families, whereas the IOU recognized 234 families (Del Hoyo and Collar 2014, 2016; Gill and Donsker 2017). Especially within passerines, the number and identity of recognized bird families are currently very dynamic and the number of families has increased continuously over the last 10 years. In 2012, when we started this study, our avian tree of life would have represented 97% (214 of 220) of all IOU families, which is 91% (214 of 234) of all IOU families recognized in 2017 (Gill and Donsker 2012, 2017). Thus, we are missing certain families, because they were only recognized after our study began. This is primarily due to a split of previous families into several new families, most with just one or very few species (species number of the missing families: 2 ± 1 [mean ± SD] species). We use the definitions of bird “families” and bird “orders” according to the IOU and HBW lists but use the terms “family-level” and “order-level” to hint to the arbitrary nature of these higher-level clades, which is reflected in the ever-changing number of avian families recognized by the above-mentioned standard references.

We sequenced RNA of 308 bird species, included published transcriptomes of 80 bird species, and 3′-UTR sequences extracted from genomes of 66 (64 bird and two alligator species) species of which seven bird species were sequenced by us and the others were publicly available (supplementary table S1, Supplementary Material online). From 27 of these 429 species, we had either two transcriptomes or a transcriptome and a genome-derived sequence (supplementary table S1, Supplementary Material online). Of nine family-level taxa, we had only genome-derived data available for the bioinformatics analysis (the seven MPIO-sequenced genomes, Acanthisittidae, and Mesitornithidae; supplementary table S1, Supplementary Material online). In total, in the construction of the molecular trees, we included RNA sequences, or their orthologous genome-derived sequences of 429 bird species comprising all avian orders (supplementary table S1, Supplementary Material online). Thus, if we consider family-level taxa recognized by the different nomenclatures, we studied between 209 and 221 bird family-level taxa: 209 of 227 recognized by both IOU and HBW, 214 of 234 recognized by IOU, 215 of 243 recognized by HBW, and 220 of 250 recognized by either IOU or HBW. Furthermore, we suggest one additional family not recognized by either HBW or IOU, the Hyliidae, first suggested by Bates (1930). In the shown family-level trees (fig. 3 and supplementary fig. S6, Supplementary Material online), all 221 potential families are labeled as such.

### RNA Preparation and Sequencing

Isolation of RNA was carried out using Qiagen RNAeasy Mini Kits (Cat No. 74106) according to the manufacturer’s instructions following the optional DNAse digestion step using 20 mg of tissue or 50 μl of blood. Blood samples were processed with Sigma TRI Reagent BD (T3809) according to manufacturer’s instructions. The RNA was extracted from the aqueous phase according to the protocol of the Qiagen RNAeasy Mini Kit (74106). The RNA quality was assessed with the Agilent 2100 Bioanalyzer Instrument (Model G2939A, Agilent Technologies RNA). Concentrations were measured with the Nanodrop 1000 spectrometer (Thermo Fisher Scientific). About 1 µg of total RNA per sample was used to construct RNA-sequencing libraries using the Trueseq RNA Sample Preparation Kit, v2 (Illumina Inc., San Diego, CA). The resulting libraries were barcoded and analyzed on Illumina Hiseq 2500 and HiSeq 4000 systems. The sequencing protocol was set to high output mode with paired-end 50- or 75-bp reads. We aimed at an output of 60–100 million reads per sample.

### De Novo Transcript Assembly

RNA-sequencing short-read data were de novo assembled using the IDBA transcriptome assembler version 1.1 (Peng et al. 2012). We used default parameters for the transcriptome assembly. Assembled transcripts were clustered using cd-hit-test (Li and Godzik 2006; Fu et al. 2012) to filter for the longest assembled transcript of a cluster of alternatively spliced/assembled transcripts. The qualities of transcriptomes were measured by basic statistics (N50 transcript length, total assembled transcript length) as well as BUSCO (gene numbers; default transcriptome parameters using the aves_odb9 gene set; Simão et al. 2015) and by counting nucleotide matches of 3′-UTR and of CDS with the canary reference genome. The reads of all sequenced species have been provided to the Sequence Read Archive of NCBI. Transcriptome de novo assemblies are available at Dryad (doi: 10.5061/dryad.ngf1vhhpx).

### Genomic Data Sources

To extend our species list, we extracted the putative transcriptomes (i.e., sequences homologous to those of our sequenced transcriptomes) from published bird genomes (supplementary table S1, Supplementary Material online). Genome assemblies (57 bird species, two alligators) were downloaded from NCBI/ENSEMBL repositories. The seven de novo assembled genomes are available at Dryad (doi: 10.5061/dryad.ngf1vhhpx) (supplementary table S1, Supplementary Material online).

### Transcript and Genome Multiple Alignments to Reference Genome

The canary (*Serinus canaria*) genome was used as reference genome during the subsequent mapping steps of all transcriptomes (supplementary fig. S1, Supplementary Material online). To construct pairwise alignments of genomes and transcriptomes, we used LAST aligner version 266 (Kielbasa et al. 2011), as it provides high sensitivity to align even distantly related genomes and transcriptomes in a computationally effective manner. Output MAF (multiple alignment format) was filtered for orthologous alignments using single_cov2 from the TBA/MULTIZ package (Blanchette et al. 2004) (two-way filtering, ref→query and query→ref). The pairwise transcriptome/genome alignments were combined into a multiple genome alignment using MULTIZ. All required steps were run on split parts of the reference genome by custom scripts using GNU PARALLEL (Tange 2011) to enable the use of multithreaded CPUs. The final MAF is available from Dryad (doi: 10.5061/dryad.ngf1vhhpx).

### Extraction of Coding, Noncoding, and Codon-Based Multiple Alignments

We used our annotation of the canary genome (http://public-genomes-ngs.molgen.mpg.de) to define bed files with coordinates of the coding, 3′-UTR and 5′-UTR, intronic and intergenic regions of the genome. The mafsInRegion tool from the Kent utilities (Kent et al. 2002) was used to extract the different fractions of the genome into coding/noncoding MAF files according to the bed files. Further processing of the alignments included removing alignments that did not align with an outgroup species to remove potential reference bias; here, we used the ostrich (*Struthio camelus*) as a “must match.” Both the coding and noncoding multiple alignments were written into a concatenated multiple fasta alignment file using mafToFa (Kent utilities) followed by custom scripts for concatenation and adding “-” characters for missing data to the sequence.

To generate a codon-based alignment, we extracted coding exons for each gene from the coding MAF file, using bed-files defining the coding exons in the canary genome for plus and minus strands separately. Exons of each gene were concatenated. Afterward, minus-strand gene sequences were reverse complemented by SEQTK. The coding gene sequences were aligned by BLAT (Kent 2002) against their corresponding canary protein sequence to identify and remove frameshifts by custom scripts. The final multiple codon alignment of gene sequences was performed by TranslatorX (Abascal et al. 2010) choosing MAFFT for multiple alignment (Katoh and Standley 2013). All codon aligned genes were concatenated into a large alignment which was used to translate codons into a multiple amino acid alignment or to extract files containing codon positions (1, 2, or 1 + 2).

### Finding a Suitable Gap versus Data Content for the Multiple Alignments

We filtered the multiple alignment fasta files to allow only a certain amount of missing data per column. In this regard, we generated multiple alignments with the following numbers of allowed gaps per alignment column: 10, 20, 40, 60, 80, 90, 100, 110, 120, 140, and 160. Alignments for 3′-UTR, CODON, or AAS with different gap cut-offs are available from Dryad (doi: 10.5061/dryad.ngf1vhhpx). For each alignment, ten trees were calculated using different maximum parsimony starting trees and RAxML (v8.2.4; Stamatakis 2014) for fast approximate tree inference (parameter: –f E –m GTRCAT or PROTCAT for AAS) and subsequent nearest neighbor interchange (NNI) refinement and SH-aLRT support calculation (parameter –f J –m GTRGAMMA or PROTGAMMA for AAS). RAxML and other tools for ML tree inference use heuristic methods to infer tree topologies and are often unable to find the best fitting tree topology by a single run. We found that for our data set ten replicates were a good trade-off between computational time needed and probability of finding the best fitting topology when using 3′-UTR alignments. To assess tree topology convergence, ten trees for each alignment file were compared with each other by calculating pairwise Robinson–Foulds (RF) distances (RAxML –f r option).

Additionally, we computed coalescent consensus trees for the ten trees per alignment file (subsets) by ASTRAL-III v5.6.1 (Zhang et al. 2018). We then calculated the RF distances of the neighboring subset coalescent trees (subset1 vs. subset2; subset2 vs. subset3;…).

### Phylogenetic Tree Calculations by Concatenated Alignments

Computing time is a major bottleneck in large phylogenomic projects. Replicates of our large data sets with millions of aligned bases for >400 species can so far only be efficiently computed (as described above) using fast approximate methods and using NNI-optimization and SH-aLRT calculation under the GTRGAMMA model instead of a standard bootstrapping method to calculate branch supports. To further improve trees derived from the fast method, candidate trees (derived from alignments with gap cut-off [100, respectively, 110] and exhibiting the best LogLH scores after NNI-optimization under GTRGAMMA) were subjected to a thorough optimization using RAxML-NG (Kozlov et al. 2019). After this final optimization, topological changes were zero for 3′-UTR underlining that the fast method was equivalent to the exhaustive method in case of 3′-UTR. Few changes were observed for CODON, CODON, and AAS trees (17, 14, and 6 of 451 splits changed, respectively) underlining that the phylogenetic signal is more difficult to resolve in these cases. In figures 2 and 3 and supplementary figure S6, Supplementary Material online, we show statistical support values for gappiness 100 (current data set of 429 species) and for gappiness 110 (a previous data set of 427 species).

To corroborate the 3′-UTR tree SH-aLRT branch supports by another method, we calculated 1,000 UFBS trees using IQTREE2 (Minh et al. 2020). We considered all branches as highly supported if the SH-aLRT values reached the maximum value (100). For branches not meeting this criteria, SH-aLRT and UFBS values are shown in the trees (figs. 2 and 3*A* and *B*).

To test, if other evolutionary models than GTR (Tavaré 1986) would fit better to our alignments, we split the concatenated alignment into chunks of 10 kb and performed iqtree model test on each chunk. For the 3′-UTR, the best fitting model to the majority of chunks was the GTR model (51.2%), followed by TVM (18.6%) (Posada 2003) and SYM (18.2%) (Zharkikh 1994) models (supplementary fig. S6, Supplementary Material online). For CDS, the SYM or GTR models were the best models for a nearly equal fraction of chunks (34.8% and 33.5%, respectively) (supplementary fig. S6, Supplementary Material online). We refined the best-scoring tree topology obtained from CDS and the GTR model by using RAxML-NG exhaustive tree topology search with the SYM model, which did not change the tree topology.

Depicted are trees that either contain all species (supplementary fig. S6, Supplementary Material online) or, for clarity, only one species per family (fig. 3*A* and *B*) or just order-level taxa (fig. 2 and supplementary fig. S4, Supplementary Material online). The trees not showing all species were prepared using FigTree (http://tree.bio.ed.ac.uk/software/figtree/; last accessed August 4, 2020) and jsTree (http://lh3lh3.users.sourceforge.net/jstree.shtml; last accessed August 4, 2020) from the all-species-trees. The depicted statistical values (fig. 3*A* and *B*; supplementary fig. S6, Supplementary Material online) are derived from the all-species-trees.

### Final Phylogenetic Species Tree Calculations by a Coalescent Approach

Besides the tree inference from concatenated alignments, we also tested inferring the species tree using a coalescence approach. We calculated 5,127 trees for 3′-UTRs of distinct gene models using iqtree (version 1.6.12; parameters: -alrt 1000 -m GTR+R4+FO). These trees were used to compute a species tree by ASTRAL (version 5.6.1; default parameters). We calculated coalescent trees from different amounts of input gene trees (252, 471, 862, 1,600, 3,200, and 5,127), which were also sorted by the amount of input data from which they were derived (highest first). Thus, we had a lower number of higher quality gene trees from longer 3′-UTR alignments and a high number of lower quality trees from shorter 3′-UTR alignments. We observed the highest similarities (RF: 97.8%) between coalescent species trees calculated from 471, 862, and 1,600 input gene trees. Increasing numbers of gene trees in the calculations (>1,600) reduced the similarity between repeated tree calculations (data not shown).

### Time-Calibrated Phylogenetic Tree

We used DPPDiv (Heath et al. 2012) for time calibration of our family-level phylogeny, which used the DPP model or the UGR model. This tool has the advantage of being able to use parallel computation. Nevertheless, we had to downscale the included amount of data to an alignment length of 10,000–100,000 nucleotides to finish computation within a reasonable amount of time (several days to weeks) on high-power computing servers (96 or 192 CPU threads). Calculations were performed twice using different starting conditions (with/without parameter: -ubl) and we checked for convergence for both, the DPP model and the UGR model (parameter: -urg), named UGR model (Heath et al. 2012). The analyses were run until linear correlation of the median divergence times between the two runs of the same model reached *R*^2^ values >0.99 and a slope of 1.0. Eighteen nodes in the tree were calibrated with fossil data, which were also used before (Jarvis et al. 2014); the divergence date for the split of pigeons and Mirandornithes was omitted due to differences between the Jarvis tree (Jarvis et al. 2014) and our 3′-UTR tree (fig. 3*A*). The time-calibrated tree was visualized with FigTree (http://tree.bio.ed.ac.uk/software/figtree/; last accessed August 4, 2020).

### Extracting 3′-UTR Sequences from the RNAseq Assemblies for the Detection of Transcription Binding Sites and Transcription Binding Sites Models

To detect phylogenetically relevant signals in 3′-UTRs, we compared species of the orders Charadriiformes, Caprimulgiformes, and Passeriformes. For family comparisons within the Passeriformes, we compared species of the Estrildidae, the Fringillidae, and of Basal Oscines; the latter is an artificial group including species of the basal radiations of oscines (see supplementary table S3, Supplementary Material online for species). We compared the 3′-UTRs of 97 randomly selected genes (supplementary table S3, Supplementary Material online) among the species.

We carried out analyses using the Genomatix software suite (Precigen Bioinformatics Germany GmbH) combining several mining sources (overrepresentation of transcription binding sites and MatInspector tools). In order to determine the occurrence of TFBS, we searched binding elements in the extracted 3′-UTRs using the Over-Represented TFBS tool (MatBase genomatix definition, Genomatix), which selects the presence of TFBS within the input sequences, generates statistics of single TFBS and calculates *Z*-scores of the representation of TFBS based on the TFBS abundances in the whole zebra finch genomic sequence (Ho Sui et al. 2005). The TFBS occurrences were calculated with MatInspector. The *Z*-score correlation graph (fig. 1*D*) was produced using the Estrildidae as a reference.

To investigate the pattern of TFBS of genera and families, we pursued deeper analysis of 3′-UTRs using the FrameWorker-Genomatix tool suite (Precigen Bioinformatics Germany GmbH). FrameWorker calculates the most complex models of TFBS that are common to sequences of the included species. Models are defined as all TFBS that occur in the same order and in a certain distance range in all (or a subset of) the input sequences (Cartharius et al. 2005). As an example, we analyzed the TFBS within the family Spheniscidae (supplementary fig. S7, Supplementary Material online). For a genus-specific model of TFBS, we compared species of the genera *Aptenodytes*, *Eudyptes*, and *Pygoscelis* using the EMC1 3′-UTR sequences. EMC1 codes for subunit 1 of the endoplasmatic reticulum membrane protein complex.

### Literature-Based Analysis of Singing

Vocal production learning, abbreviated in this paper as vocal learning, was considered present in a species, if studies had reported imitation of conspecifics, mimicry of heterospecifics, or mimicry of nonbird sounds in that species, or if studies had reported local dialects in that species. As sources, we studied all available publications as well as various encyclopedias, the HBW, the Handbook of Western Palearctic Birds, the Handbook of Australian, New Zealand and Antarctic Birds, and The Birds of Africa. For the family-level analysis, we considered vocal learning as present in a family, if at least one species of a family fulfilled the criteria above. The family-level taxa and related references to vocal learning are listed in supplementary table S5, Supplementary Material online. To test the association between the phylogenetic tree and the occurrence of vocal learning, in the family-level taxa, we used TreeBreaker (https://github.com/ansariazim/treeBreaker; last accessed August 4, 2020), an inference procedure based on a Bayesian statistical method (Ansari and Didelot 2016). The software uses a Bayesian model to deduce whether the phenotype of interest is randomly distributed on the tips of the tree and to estimate which clades, if any, have a distinct distribution from the rest of the tree (Ansari et al. 2019).

## Data Availability

Transcriptome assemblies used in this study have been made available through as a Dryad archive (https://doi.org/10.5061/dryad.ngf1vhhpx).

## Supplementary Material

Supplementary data are available at *Molecular Biology and Evolution* online.

## Author Contributions

H.K.: design of bioinformatic pipeline, data processing and analysis, and manuscript writing. C.F.: comparative analysis of 3′-UTR structure. A.B.: preparation of all RNAs and DNAs. G.M.: evaluation of time-calibration and fossil data. G.N.: tissue sampling. S.T.B., S.K., and B.T.: sequencing. M.G.: concept, tissue sampling, meta-analysis of vocal learning, and writing of the manuscript.

## Supplementary Material

msaa191_Supplementary_Data

## Notes

Transcriptome sequencing reads have been deposited under the BioProject accession PRJNA599522.
