# Supplementary material for: An Unbiased Molecular Approach Using 3′-UTRs Resolves the Avian Family-Level Tree of Life
Source: Mol Biol Evol. 2020 Nov 8;38(1):108–27. doi: 10.1093/molbev/msaa191 (PMC7783168; doi:10.1093/molbev/msaa191)
Supplement: msaa191_Supplementary_Data [file msaa191_supplementary_data.pdf]

Supplementary Information

An Unbiased Molecular Approach Using 3'-UTRs Resolves the Avian Family-Level Tree of Life

Kuhl H[1,2,3], Frankl-Vilches [C1], Bakker A[1], Mayr G[4], Nikolaus G[1], Boerno ST[2], Klages S[2], Timmermann B[2], Gahr M[1].

1: Max Planck Institute for Ornithology, Dept. Behavioural Neurobiology, Seewiesen, Germany; 2: Max Planck Institute for Molecular Genetics, Sequencing Core facility, Berlin, Germany; 3: Leibniz-Institute of Freshwater Ecology and Inland Fisheries, Dept. Ecophysiology and Aquaculture, Berlin, Germany. 4: Senckenberg Research Institute, Ornithological Section, Frankfurt am Main, Germany

**1. Supplementary Figures - Legends**	page 2-5
**2. Supplementary Tables - Legends**	page 6-7
**3. Supplementary Results and Discussion**	page 8 -12
**4. Supplementary Figures**	page 13-20
**5. Supplementary Tables**	page 21-42

Supplementary Figure legends:

Fig. S1: The steps used to combine transcriptomic and genomic assemblies into multiple alignments for phylogenetic analysis.

Fig. S2: Transcriptome size of various tissues (A) and the effect of gap filters (gappiness) on tissue specific transcriptome sizes (B). In **A** we show that the transcriptome sizes and the resulting amount of non-coding 3'-UTR sequences (blue) and of CDS (coding) sequences (red) differed between tissue types. Brain samples resulted in the largest transcriptomes and blood samples in the smallest transcriptomes based on the amount of alignable sequence in the reference genome (depicted is the median and error of the median of nucleotides aligned to the canary reference genome). For each tissue type, the fraction of nucleotides aligned to CDS or 3'-UTR was similar, i.e. was not tissue type specific. Miscellaneous tissue types were named "body"; these tissues were neither brain nor blood and mainly represent the museum specimens. The numbers on the columns represent the sample size per tissue (Tab. S2). Statistical comparison (ANOVA followed by Tukey HSD, $p = 0.001$, $F (5, 299) = 30.14$; see Tab. S2D for data) showed that the transcriptome sizes of brain tissue were similar to skin but differed from all other tissues and that blood, skin, liver and muscle derived transcriptomes were similar (tissue types with the same letter are not significantly different). However, note the small number of skin tissues. In **B**, we depict that the application of gap filters (gappiness) of around 100 missing nucleotides per reference nucleotide (dashed circle) normalizes the size of the used transcriptomes. Shown are the mean numbers of aligned nucleotides per tissue for 3'-UTRs; "all" is the combined aligned transcriptome of all tissues. Essentially, the gappiness criteria removes nucleotides from the multiple alignment that are aligned in only few samples due to differences in transcriptome sequencing quality and tissue specific or species specific expression. Statistical comparison (ANOVA followed by Tukey HSD, $p<0.0001$, $F (4, 282) = 7.2317$; see Tab. S2E for data) showed that the transcriptome sizes resulting of gap filtering (gappiness 100) of each tissue-specific transcriptome were similar for blood, brain, liver and muscle. Only those of "body" differed from the other tissues due to a few smaller transcriptomes. Thus, transcriptomes derived of blood are as good as those derived from other organs for phylogenetic procedures.

Fig. S3. GTR bioinformatics evolutionary model was the best model independent of sequence type. The GTR evolutionary model performed best for the majority of the 10 kbp chunks of the 3'-UTR data. In case of CDS (coding) data, SYM and GTR-F models performed similar, while independent calculations using GTR-F or SYM models resulted in the same tree topology. For testing, the

concatenated alignments were split into 10 kbp chunks to perform iqtree model tests on each chunk. Abbreviations: GTR = generalized time-reversible model, Tavaré, 1986; SYM = symmetrical model, Zharkikh, 1994. All models are described in http://www.iqtree.org/ doc/Substitution-Models.

Fig. S4: Phylogenetic trees derived from coding sequences (CODON, **A**), coding sequences with the 3rd nucleotide removed from the codons (CODON12, **B**), amino acid sequences (AAS, **C**), all calculated with a concatenated bioinformatics procedure, and a noncoding sequence tree (3'-UTR, **D**) calculated with a coalescent bioinformatics procedure. The color code refers to the higher-level clades identified in the 3'-UTR concatenated tree (Fig. 2): blue = Australaves; green = Higher Landbirds; red = Aquatic & Semiaquatic Birds; yellow = Basal Landbirds; grey = Mirandornithes; light blue = Galloanserae; brown = Palaeognathae. Note that the higher-level taxa of Fig. 2 are mixed up in A-D. Furthermore, the number and composition of higher-level clades of A-D vary. Note that the families of the order Caprimulgiformes (*) are split into two non-related taxa in the CODON tree (A). The Falconiformes (#) are moved away from the Australaves in A-C and are fused to some (B) or all (C) other orders of birds of prey. However, in the CODON12 tree (B) the Cariamiformes are now sister to Accipitriformes, but not the Falconiformes as in the 3'-UTR concatenated tree of Fig. 2 or as suggested previously (e.g. Suh et al., 2011). Orders with more than one species were collapsed to one branch. Branch lengths of the trees are not scaled to facilitate illustration.

Fig. S5: The difference of our 3'-UTR tree as compared to the "Jarvis" tree (which combined coding and noncoding sequences) is due to sequence type and taxa sampling. Depicted are phylogenetic trees derived from noncoding sequences (3'-UTR) using different amounts of taxa or tree calculations and collapsed to the taxa included in the Jarvis tree (Jarvis et al., 2014). In **A** we show that the 3'-UTR tree differs from the Jarvis-tree even if we consider only those 50 species (taxa) represented in the Jarvis-tree. This is possibly due to differences in type of data. In **B**, we increased the number of taxa to 96 by adding taxa that were most closely related to the taxa used by Jarvis et al. (in most cases transcriptomes of the very same genus were added to the calculation). This resulted in a tree most similar to the Jarvis tree and shows that restrictions of data types and amount may possibly be compensated by biological replicates of species used for the tree calculation. In **C**, we increased the number of taxa to 121 by adding those taxa, which had the most basal split in each order. This already resulted in a topology very similar to our final topology (D) and shows the importance taxon sampling. In **D** we included all 429 species plus some biological replicates (452 samples). [!] indicates differences of these 3'-UTR trees (A-D) as compared to the Jarvis-tree that was calculated based on a mixture of coding and noncoding sequences. Of the 50 taxa represented in the Jarvis tree, most

differences with our tree concern the relationships of owls among the Higher Landbirds, of the taxa of the Basal Landbirds, and of the basal position of the Mirandornithes among the Neoaves (see Fig. 2).

Fig. S6: A species-level phylogeny of birds (429 species of 379 genera) based on 3'-UTR sequences including all (106) non-passerine and most (115) passerine family-level taxa. Each species is represented by one species, listed as the species name, followed by the family name and the order name. The higher-level clades are color-coded as in Fig. 2 (right column). The suborder Passeri (oscines or songbirds) of the Passeriformes is subdivided into ten oscine-higher clades (OHC1 to OHC10). The intrafamily relationships of the Fringillidae (#) and the Paridae ($) have been studied in some detail and fit to previous assumptions. The questionable split of the Cettiidae and Scotocercidae into separate families is indicated (*). For all genera represented with two or more species, such species were correctly assigned to the expected genus. In a number of cases, species were represented by both transcriptomes and genomes (see Tab. S1). The tree was calculated by RAxML-ng using a large concatenated alignment of 3'-UTR residues as input (2,584,785 analyzable patterns, maximum 100 or 110 missing taxa (gappiness). Approximate likelihood-based measures of branch support delivered maximal values (SH-aLRT = 100) except those shown in red (for 110-gappiness) and blue (for 100-gappiness). SH-aLRT values are considered as quite conservative. In case of SH-aLRT values below 100, we also provide support values from IQTREE2 ultrafast bootstrapping (UFBS, green values). In the few cases were SH-aLRT support was below 80 (two for 110-gappiness; seven for 100-gappiness), the UFBS approach still reached good values of support in the range of 86 – 99. Note that the entire backbone of the phylogenetic tree had maximal support (SH-aLRT and UFBS = 100) values. The timing of the divergence nodes was calculated by DPPDiv as detailed in the methods Error bars are confidence intervals (95%). Time scale and divergence times are in Million years ago. Diagonal bars indicate the part of the tree that is not scaled in order to reduce the size of the tree and PDF.

Fig. S7: The pattern of transcription factor binding site motifs (TFBS) of a 3'-UTR differs in genus-specific manner within the family Spheniscidae (penguins). The 3'-UTRs of the gene EMC1 of seven species of three genera of penguins are depicted schematically. The presence of TFBS in that 3'-UTR shows a family-specific signature in all species (**A**) as well genus-specific signatures for each of the three genera, the *Aptenodytes* (**B**), the *Eudyptes* (**C**) and the *Pygoscelis* (**D**). The combinatorial pattern of the TFBS distinguished the genera. TFBS that occur in all or several species are aligned above the black line, those that occur in only one species are listed below the black line.

Fig. S8: Vocal production learning evolved three times during avian evolution, (1.) in hummingbirds (Fam. Trochilidae) nested deeply in the Caprimulgiformes, (2.) in the ancestor of parrots and thus is present in the three parrot families of the Psittaciformes, and (3.) in the ancestor of the oscine passerines, the songbirds, and thus is present throughout the songbird families but absent in suboscine passerine families. The thickness and redness of the branches are proportional to the posterior probability of having a change-point assessed by TreeBreaker algorithm, a Bayesian inference calculation method. This analysis indicates that the alternative model is supported, i.e., the distribution of vocal production learning at the tips of the branches is different under those clades as compared to the null model of no association. Turquoise dots label family-level taxa with vocal production learning (Tab. S5), green dots those that lack vocal production learning (detailed information about song without mentioning vocal learning), and dark blue dots those of which information concerning singing and vocal production learning is widely missing. The phylogenetic tree used for the calculations is the 3'-UTR family-level tree (Fig. 3A & 3B). The diagonal bar indicates that this part of the family-level tree of avian life is not scaled.

Supplementary Tables

Tab. S1: List of species, family-level tax, order-level taxa and the source of tissues, transcriptomes and genomes.

Tab. S2: Quality of transcriptome and genome assemblies included in the present study using idba_tran or idba_ud, respectively. (**A**) shows basic features of the transcriptomes generated by us, (**B**) of the publically available transcriptomes, and (**C**) of the genomes generated by us. (**D**) shows statistical analysis (ANOVA followed by Tukey HSD test) of basic transcriptome features (consensus length, N50 transcript length, number of assembled transcripts). The complexity of transcriptomes (i.e. number of genes) was verified by BUSCO and alignment with the reference genome to estimate the amount of uniquely alignable CDS and 3'-UTR sequence (A, B). The same statistics were calculated for draft genome assemblies (C), but neglecting the BUSCO scoring, as BUSCO does not work well on highly fragmented genome assemblies. (**E**) shows the transcriptome length after gap filtering for various levels of gappiness (number of allowed missing samples) for the various tissues. Statistical analysis (ANOVA followed by Tukey HSD test) showed that only "body" differs significantly from the other tissues due to a few species for which gappiness resulted in smaller transcriptomes; statistics shown is for 100 missing samples (gappiness 100).

Tab. S3: List of species (**A**) and genes (**B**) used for the analysis of transcription factor binding site motifs (TFBS) of 3'-UTRs (see Fig. 1D).

Tab. S4: Comparison of fossil data and divergence times of family-level taxa as estimated by the UGR (uncorrected gamma-distributed rate model). We considered well-documented and temporally well constrained fossils, which represent either crown- or stem-group representatives of certain clades. Molecular data are taken from Fig. S6. For fossils, whose age is determined as a time range, we calculated the minimal and the maximal differences between fossil and molecular age. From these values, we calculated the mean difference between molecular and fossil data as being 9.4 ± 8.8 My (minimal; mean ± SD) and 10.7 ± 9.5 My (maximum; mean ± SD), respectively. For simplicity, we did not consider confidence intervals of the molecular-dated clades.

Tab. S5: List of songbird family-level taxa with at least one species reported to show vocal production learning, as indicated by mimicry, experimental evidence or dialect formation (see Fig. S8).

Tab. S6: List of species with anatomical evidence for the presence of the song control system of oscine passerine family-level taxa.

Supplementary Results and Discussion

The avian family-level tree of life.

The interrelationships of the family-level taxa of Clade 1, the Palaeognathae (Fig. 3A) agree with the phylogeny of Prum (Prum et al., 2015). The ostriches are the sister group of all other palaeognath birds and the volant tinamous (Tinamidae) are nested within the clade. The South-American tinamous are the sister group of the Australian cassowaries and emus (Casuariidae). These relationships differ from those reported on basis of conserved noncoding elements and a coalescent inference procedure, in which the rheas are sister to the cassowaries, emus and kiwis (Sackton et al., 2019). The differences are due to the tree inference procedure. The divergence time (58 Mya) of the ostriches (Struthionidae) from the tinamous (Tinamidae), rheas (Rheidae) and cassowaries (Casuariidae), as well the divergence times of the rheas, tinamous, and cassowaries (51-45 Mya) are earlier than previously suggested (Prum et al., 2015), which matters for the scenario of evolution of flightlessness of palaeognath species (see Supplementary discussion)

The interrelationships within Clade 2, the Galloanserae (Fig. 3A), which include the Galliformes (landfowl) and Anseriformes (waterfowl), are also in concordance with previous analyses (Hackett et al., 2008; Prum et al., 2015). The South American screamers (Anhimidae) are the sister group of all other Anseriformes. Among the Galliformes, the megapodes (Megapodiidae) are the sister group of all other species.

For the Neoaves (Clades 3-7; Fig. 3A), the relationships of the included taxa are partially already described in the main part of the paper. Thus, for Clade 4 we refer entirely to the main paper.

Clade 5 (Fig. 3A) of our phylogeny comprises the Aquatic & Semiaquatic Birds, which fall into two subclades. One subclade includes most seabirds (Fig. 3A, Clade 5B) (Gaviiformes, Sphenisciformes, Procellariiformes, Suliformes, Pelecaniformes) as well as storks (Ciconiiformes). The other subclade (Fig. 3A, Clade 5A) comprises the tropicbirds (Phaethontiformes), and the kagu and sunbittern (Eurypygiformes). Clade 5 corresponds with the clade "Core waterbirds" of the Jarvis et al (2014) tree - although the latter does not include Ciconiiformes, which were not included in the study - but differs entirely in its composition from the waterbird clade recovered in Prum's phylogeny (Prum et al., 2015). In that clade, Prum's phylogeny combines the Charadriiformes, the Mirandornithes, and those orders making-up our Aquatic & Semiaquatic Birds clade. A sister group relationship between storks and the seabird clade has been suggested before (Kuramoto et al., 2015).

In Clade 6 (Fig. 3A), the Higher Landbirds, the hawks, eagles, and the new world vultures (order Accipitriformes) (Fig. 3A) are the sister group of an assemblage of seven orders (Fig. 3A), the owls (Strigiformes), mousebirds (Coliiformes), cuckoo rollers (Leptosomiformes), trogons

(Trogoniformes), hornbills (Bucerotiformes), rollers and allies (Coraciiformes) and the woodpeckers and allies (order Piciformes). This Higher Landbirds confirm to the Afroaves clade of Jarvis et al (2014), while the included orders were subdivided into two (Prum et al., 2015) or three (Hackett et al., 2008) higher-level clades in other studies.

Clade 7 (Fig. 3B) consists of the Australaves (Suh et al., 2011; Hackett et al., 2008) that include the seriemas (Cariamiformes), the falcons (Falconiformes), the parrots (Psittaciformes) and the passerines (Passeriformes), which is consistent with previous phylogenetic results (Hackett et al., 2008; Suh et al., 2011, Jarvis et al., 2014; Prum et al., 2015). However, some family relationships of oscine passerines are different in our 3'-UTR tree as compared to previous trees (e.g. Prum et al., 2015; Oliveros et al., 2019), as discussed in more detail below.

In the Passeriformes, the 92-107 studied (the count depending on the classification, see Methods) family-level taxa of songbirds (suborder Passeri [or oscine passerines] of the Passeriformes) fall into ten higher-level oscine clades (OHC) (Fig. 3B; Fig. S6). The basal OHC1 (Fig. 3B) represents the lyrebirds (Menuridae); OHC2 (Fig.3B) the Australian treecreepers and bowerbirds (Climacteridae and Ptilonorhynchidae); OHC3 (Fig.3B) unites the Fairy-wrens, the honeyeaters, pardalotes and Australian "warblers" (Maluridae, Meliphagidae, Pardalotidae and Acanthizidae); OHC4 (Fig.3B) are the Australo-Papuan babblers and longrunners (Pomatostomidae and Orthonychidae); OHC5 (Fig. 3B) comprises most of the families formerly summarized as parvorder Corvida (Sibley and Ahlquist, 1991; Barker et al., 2004); OHC6 (Fig. 3B) are the satinbirds (Cnemophilidae); OHC7 (Fig.3) are the berrypeckers and longbills (Melanocharitidae); OHC8 (Fig. 3B) are the Australo-Papuan robins, the African rockfowl and rockjumpers (Petroicidae, Picathartidae, and Chaetopidae) [OHC6-OHC8 were formerly grouped loosely as transitional oscines]; and the evolutionarily youngest taxa OHC9-OHC10 include most taxa previously summarized as the Passerida (Sibley and Ahlquist, 1991; Barker et al., 2004). OHC9 (Fig. 3B) comprises the Sylvioidea and Paroidea. OHC10A (Fig. 3) consists of the Muscicapoidea, Sittoidea, and Regulidae. OHC10B (Fig. 3B) assembles the Passeroidea including the nine-primaried oscines (a polyphyletic group), the Dicaeoidea, Promeropidae, and Bombycilloidea, as well as one taxon previously not assigned to the Passerida, the Chloropsidae.

The present phylogeny differs widely from all previous ones in the composition of oscine higher-level clades and the interrelationships of the family-level taxa within these clades (e.g. Barker et al., 2004; Aggerbeck et al., 2014) with the exception of the recent study of Oliveros et al (2019), which was based on ultra-conserved molecular elements. Since the Oliveros-tree of oscine families and that part of our tree are very similar, these trees might converge on the true phylogeny of songbird families. The small differences between the passerine part of the present tree and the Oliveros-tree

might be due to the use of different genera. The strong differences to other passerine trees (e.g. Barker et al., 2004; Jetz et al., 2012; Aggerbeck et al., 2014; Claramunt and Cracraft, 2015) are likely due to their lower sampling of families, the overall number of sequences analysed, and/or the use of mitochondrial and nuclear coding sequences. The future addition of further genera, in particular of the corvid relationship (OHC5) shall solve the ambiguities between the Oliveros-tree and the presented tree.

The missing oscine families might affect the relationships of some families within the OHCs described above. In particular, the lack of the scrubbird family (Atrichornithidae) is unfortunate since it is likely the sister group of the lyrebirds (OHC1) (Chesser and ten Have, 2007). However, most missing families are expected to fall within either the Corvida assemblage (OHC5) or the Passerida assemblage (OHC9-OHC10). Thus, the 10% missing families are unlikely to affect the overall structure of songbird clades (OHCs), which is quite similar to the one of Oliveros et al (2019). Of 21 recently defined songbird family-level taxa, our data confirm the split of previous families in fifteen cases. The split of the previous Timaliidae into Pellorneidae, Leiotrichidae and Timaliidae may be questioned, since these three families are each other's closest relatives (Fig. 3B & Fig. S6). Furthermore, our data strongly suggest the removal of the genus *Hylia* from the Scotocercidae into their own family, the Hyliidae (Bates, 1930; Fregin et al., 2012), but do not support the Cettiidae being its own (IOU) family, separate from the Scotocercidae (Fig S6). Otherwise, either the genus *Abroscopus* or the genus *Urosphena* of Scotocercidae need to be considered part of the Cettiidae or the genus *Urosphena* has to be assigned to a separate family-level taxon. Thus, since the Erythrocercidae were recently removed from the Cettiidae (Gill and Donsker, 2017), the split of Erythrocercidae, Scotocercidae and Cettiidae altogether needs reconsideration. The split of the Thraupidae into several families could not be validated, because material was only available for the Coerebidae (banana quit), which was assigned as the closest relative of the Thraupidae.

On the subfamily level, we studied in some detail the Paridae (tits), the Fringillidae (finches), and the Estrildidae (waxbills), since these genera-rich families are rather well known. The observed relationships of the tit species/genera (Paridae) as well as that of finch species/genera (Fringillidae) matched recently published detailed molecular trees of these families using mitochondrial data (Zuccon et al., 2012; Johansson et al., 2013). For the Estrildidae, too, in all cases our tree-building procedure placed the closely related species of the same genus correctly. However, the relationship of the waxbill genera differed strongly from previous phylogenies (Sorenson et al., 2004; Arnaiz-Villena et al., 2009; Hooper and Price, 2015), which was based on little sequence information.

References

Aggerbeck M, Fjeldsa J, Christidis L, Fabre PH, Jonsson KA. 2014. Resolving deep lineage divergences in core corvoid passerine birds supports a proto-Papuan island origin. *Mol Phylogenet Evol.* 70:272-285.

Arnaiz-Villena A, Ruiz-del-Valle V, Gomez-Prieto P, Reguera R, Parga Lozano CH, Serrano-Vela J. 2009. Estrildinae Finches (Aves, Passeriformes) from Africa, South Asia and Australia: a Molecular Phylogeographic Study. *Open Ornithol J.* 2:29-36.

Barker FK, Cibois A, Schikler P, Feinstein J, Cracraft J. 2004. Phylogeny and diversification of the largest avian radiation. *Proc Natl Acad Sci USA* 101:11040-11045.

Bates GL. 1930. Handbook of the birds of West Africa. London: Bale Sons and Danielson.

Chesser R, Have J. 2007. On the phylogenetic position of the scrub-birds (Passeriformes: Menurae: Atrichornithidae) of Australia. *J Ornithol*. 148:471-476.

Claramunt S, Cracraft J. 2015. A new time tree reveals Earth history's imprint on the evolution of modern birds. *Sci Adv* 1:e1501005.

Fregin S, Haase M, Olsson U, Alström P. 2012. New insights into family relationships within the avian superfamily Sylvioidea (Passeriformes) based on seven molecular markers. BMC *Evol Biol.* 12:157.

Gill F, Donsker D. 2017. IOC World Bird List (v. 7.3). International Ornithologists' Union (doi: 10.14344/IOC.ML.7.3).

Hackett SJ, Kimball RT, Reddy S, Bowie RC, Braun EL, Braun MJ, Chojnowski JL, Cox WA, Han K-L, Harshman J. 2008. A phylogenomic study of birds reveals their evolutionary history. *Science*. 320:1763-1768.

Hooper DM, Price TD. 2015. Rates of karyotypic evolution in Estrildid finches differ between island and continental clades. *Evol*. 69:890-903.

Jarvis ED, Mirarab S, Aberer AJ, Li B, Houde P, Li C, Ho SY, Faircloth BC, Nabholz B, Howard JT, et al. 2014. Whole-genome analyses resolve early branches in the tree of life of modern birds. *Science*. 346:1320-1331.

Jetz W, Thomas GH, Joy JB, Hartmann K, Mooers AO. 2012. The global diversity of birds in space and time. *Nature*. 491:444.

Johansson US, Ekman J, Bowie RC, Halvarsson P, Ohlson JI, Price TD, Ericson PG. 2013. A complete multilocus species phylogeny of the tits and chickadees. *Mol Phylogenet Evol*. 69:852-860.

Kuramoto T, Nishihara H, Watanabe M, Okada N. 2015. Determining the Position of Storks on the Phylogenetic Tree of Waterbirds by Retroposon Insertion Analysis. *Genome Biol Evol.* 7:3180-3189.

Oliveros CH, Field DJ, Ksepka DT, Barker FK, Aleixo A, Andersen MJ, Alstrom P, Benz BW, Braun EL, Braun MJ, et al. 2019. Earth history and the passerine superradiation. *Proc Natl Acad Sci USA* 116:7916-7925.

Prum RO, Berv JS, Dornburg A, Field DJ, Townsend JP, Lemmon EM, Lemmon AR. 2015. A comprehensive phylogeny of birds (Aves) using targeted next-generation DNA sequencing. *Nature*. 526:569-573.

Sackton TB, Grayson P, Cloutier A, Hu Z, Liu JS, Wheeler NE, Garnder PP, Clarke JA, Baker AJ, Clamp N, Edwards SV. 2019. Convergent regulatory evolution and loss of flight in paleognathous birds. *Science* 364:74-78.

Sibley CG, Ahlquist JE. 1991. Phylogeny and Classification of the Birds. A Study in Molecular Evolution: Yale University Press. New Haven and London.

Sorenson MD, Balakrishnan CN, Payne RB. 2004. Clade-limited colonization in brood parasitic finches (Vidua spp.). *Syst Biol.* 53:140-153.

Suh A, Paus M, Kiefmann M, Churakov G, Franke FA, Brosius J, Kriegs JO, Schmitz J. 2011. Mesozoic retroposons reveal parrots as the closest living relatives of passerine birds. *Nature Comm*. 2:443.

Tavaré S. 1986. Some Probabilistic and Statistical Problems in the Analysis of DNA Sequences. *Lect Math Life Sci*. 17:57–86

Zharkikh A. 1994. Estimation of evolutionary distances between nucleotide sequences. *J Mol Evol* 39:315-29.

Zuccon D, Prŷs-Jones R, Rasmussen PC, Ericson PGP. 2012. The phylogenetic relationships and generic limits of finches (Fringillidae). *Mol Phylogenet Evol*. 62:581-596.

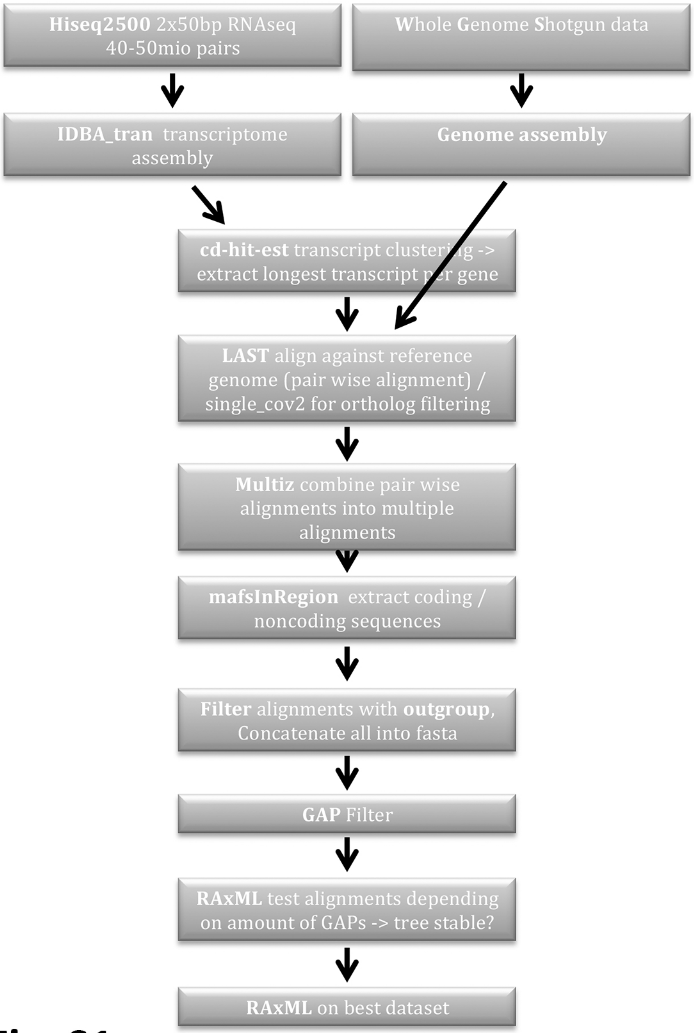
Hiseq2500 2x50bp RNAseq 40-50mio pairs
Whole Genome Shotgun data
IDBA_tran transcriptome assembly
Genome assembly
cd-hit-est transcript clustering -> extract longest transcript per gene
LAST align against reference genome (pair wise alignment) / single_cov2 for ortholog filtering
Multiz combine pair wise alignments into multiple alignments
mafsInRegion extract coding / noncoding sequences
Filter alignments with outgroup, Concatenate all into fasta
GAP Filter
RAxML test alignments depending on amount of GAPs -> tree stable?
RAxML on best dataset

Fig. S1

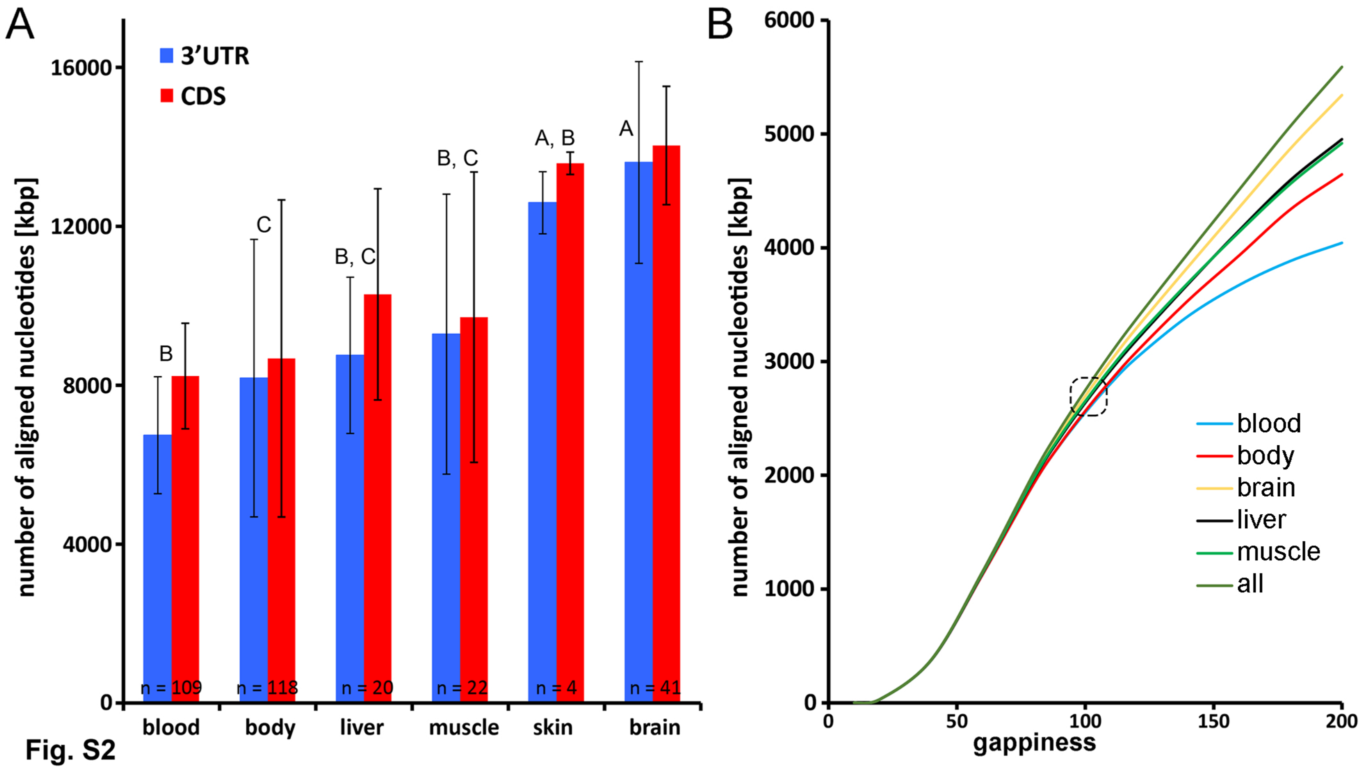
A
3'UTR
CDS
number of aligned nucleotides [kbp]
16000
12000
8000
4000
0
B
C
B, C
B, C
A, B
A
n = 109
n = 118
n = 20
n = 22
n = 4
n = 41
blood
body
liver
muscle
skin
brain
B
6000
5000
4000
3000
2000
1000
0
0
50
100
150
200
gappiness
number of aligned nucleotides [kbp]
blood
body
brain
liver
muscle
all

Fig. S2

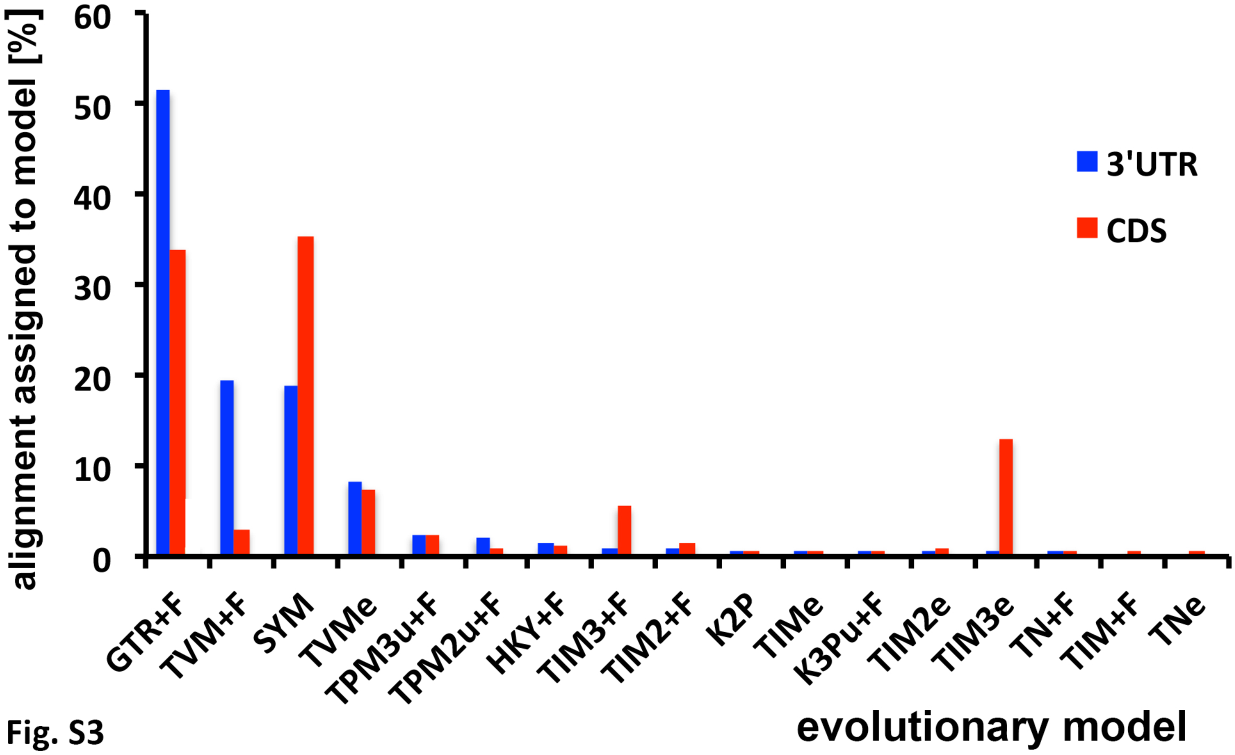

alignment assigned to model [%]
60
50
40
30
20
10
0
3'UTR
CDS
GTR+F
TVM+F
SYM
TVMe
TPM3u+F
TPM2u+F
HKY+F
TIM3+F
TIM2+F
K2P
TIMe
K3Pu+F
TIM2e
TIM3e
TN+F
TIM+F
TNe
evolutionary model

Fig. S3

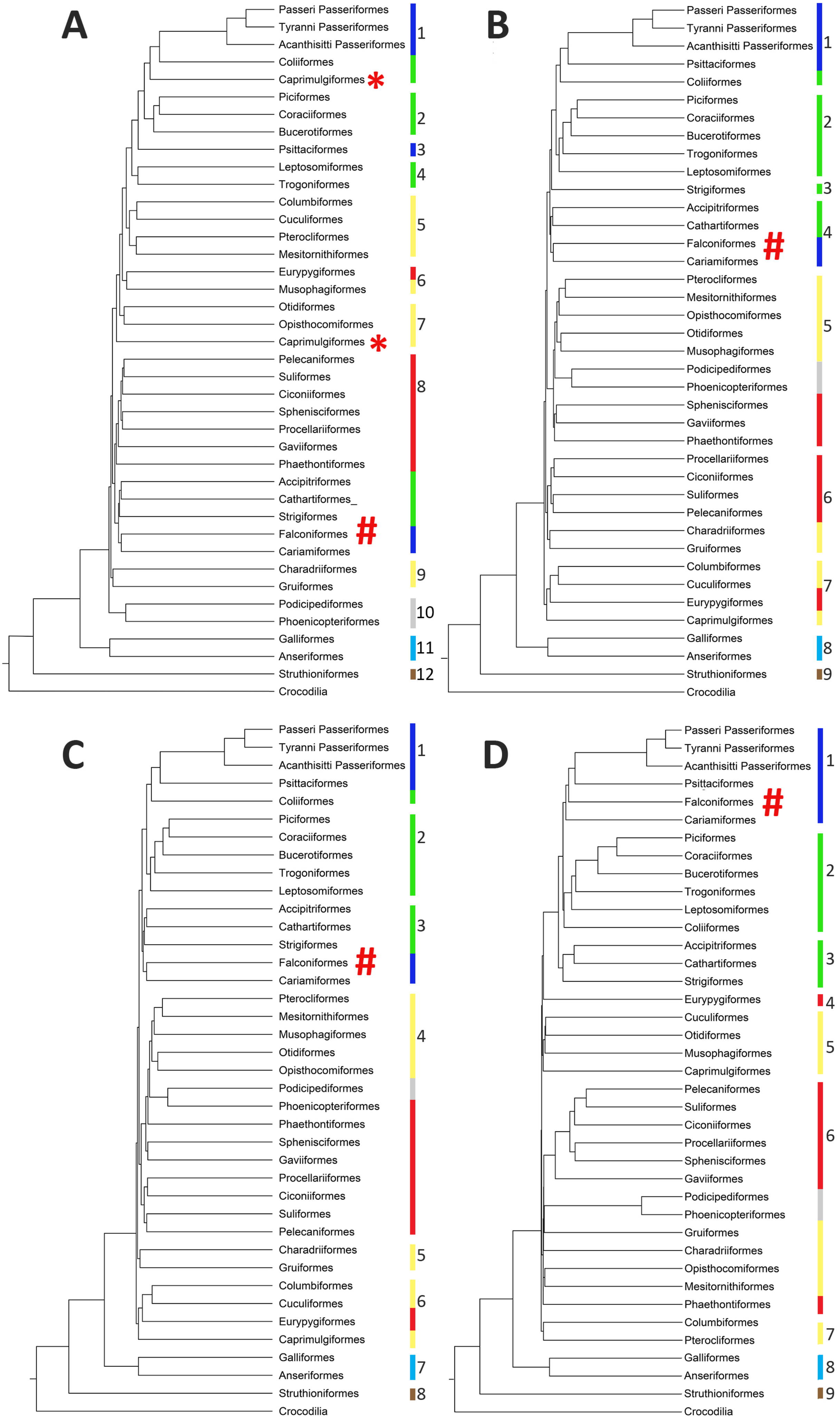
A
Passeri Passeriformes
Tyranni Passeriformes
Acanthisitti Passeriformes
Coliiformes
Caprimulgiformes *
Piciformes
Coraciiformes
Bucerotiformes
Psittaciformes
Leptosomiformes
Trogoniformes
Columbiformes
Cuculiformes
Pterocliformes
Mesitornithiformes
Eurypygiformes
Musophagiformes
Otidiformes
Opisthocomiformes
Caprimulgiformes *
Pelecaniformes
Suliformes
Ciconiiformes
Sphenisciformes
Procellariiformes
Gaviiformes
Phaethontiformes
Accipitriformes
Cathartiformes_
Strigiformes
Falconiformes #
Cariamiformes
Charadriiformes
Gruiformes
Podicipediformes
Phoenicopteriformes
Galliformes
Anseriformes
Struthioniformes
Crocodilia
1
2
3
4
5
6
7
8
9
10
11
12
B
Passeri Passeriformes
Tyranni Passeriformes
Acanthisitti Passeriformes
Psittaciformes
Coliiformes
Piciformes
Coraciiformes
Bucerotiformes
Trogoniformes
Leptosomiformes
Strigiformes
Accipitriformes
Cathartiformes
Falconiformes #
Cariamiformes
Pterocliformes
Mesitornithiformes
Opisthocomiformes
Otidiformes
Musophagiformes
Podicipediformes
Phoenicopteriformes
Sphenisciformes
Gaviiformes
Phaethontiformes
Procellariiformes
Ciconiiformes
Suliformes
Pelecaniformes
Charadriiformes
Gruiformes
Columbiformes
Cuculiformes
Eurypygiformes
Caprimulgiformes
Galliformes
Anseriformes
Struthioniformes
Crocodilia
1
2
3
4
5
6
7
8
9
C
Passeri Passeriformes
Tyranni Passeriformes
Acanthisitti Passeriformes
Psittaciformes
Coliiformes
Piciformes
Coraciiformes
Bucerotiformes
Trogoniformes
Leptosomiformes
Accipitriformes
Cathartiformes
Strigiformes
Falconiformes #
Cariamiformes
Pterocliformes
Mesitornithiformes
Musophagiformes
Otidiformes
Opisthocomiformes
Podicipediformes
Phoenicopteriformes
Phaethontiformes
Sphenisciformes
Gaviiformes
Procellariiformes
Ciconiiformes
Suliformes
Pelecaniformes
Charadriiformes
Gruiformes
Columbiformes
Cuculiformes
Eurypygiformes
Caprimulgiformes
Galliformes
Anseriformes
Struthioniformes
Crocodilia
1
2
3
4
5
6
7
8
D
Passeri Passeriformes
Tyranni Passeriformes
Acanthisitti Passeriformes
Psittaciformes
Falconiformes #
Cariamiformes
Piciformes
Coraciiformes
Bucerotiformes
Trogoniformes
Leptosomiformes
Coliiformes
Accipitriformes
Cathartiformes
Strigiformes
Eurypygiformes
Cuculiformes
Otidiformes
Musophagiformes
Caprimulgiformes
Pelecaniformes
Suliformes
Ciconiiformes
Procellariiformes
Sphenisciformes
Gaviiformes
Podicipediformes
Phoenicopteriformes
Gruiformes
Charadriiformes
Opisthocomiformes
Mesithornithiformes
Phaethontiformes
Columbiformes
Pterocliformes
Galliformes
Anseriformes
Struthioniformes
Crocodilia
1
2
3
4
5
6
7
8
9

Fig. S4

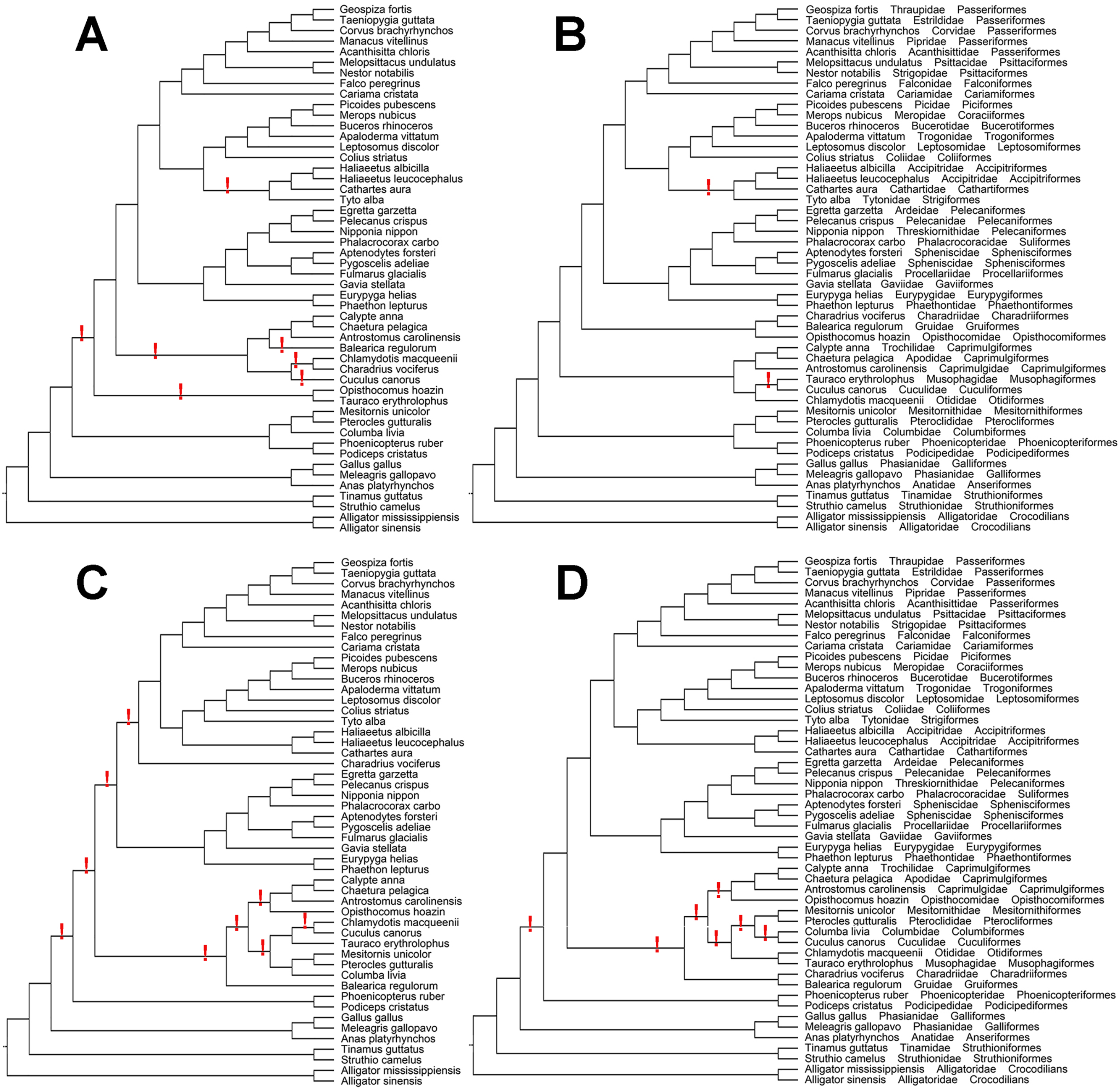
A
Geospiza fortis
Taeniopygia guttata
Corvus brachyrhynchos
Manacus vitellinus
Acanthisitta chloris
Melopsittacus undulatus
Nestor notabilis
Falco peregrinus
Cariama cristata
Picoides pubescens
Merops nubicus
Buceros rhinoceros
Apaloderma vittatum
Leptosomus discolor
Colius striatus
Haliaeetus albicilla
Haliaeetus leucocephalus
Cathartes aura
Tyto alba
Egretta garzetta
Pelecanus crispus
Nipponia nippon
Phalacrocorax carbo
Aptenodytes forsteri
Pygoscelis adeliae
Fulmarus glacialis
Gavia stellata
Eurypyga helias
Phaethon lepturus
Calypte anna
Chaetura pelagica
Antrostomus carolinensis
Balearica regulorum
Chlamydotis macqueenii
Charadrius vociferus
Cuculus canorus
Opisthocomus hoazin
Tauraco erythrolophus
Mesitornis unicolor
Pterocles gutturalis
Columba livia
Phoenicopterus ruber
Podiceps cristatus
Gallus gallus
Meleagris gallopavo
Anas platyrhynchos
Tinamus guttatus
Struthio camelus
Alligator mississippiensis
Alligator sinensis
B
Geospiza fortis Thraupidae Passeriformes
Taeniopygia guttata Estrildidae Passeriformes
Corvus brachyrhynchos Corvidae Passeriformes
Manacus vitellinus Pipridae Passeriformes
Acanthisitta chloris Acanthisittidae Passeriformes
Melopsittacus undulatus Psittacidae Psittaciformes
Nestor notabilis Strigopidae Psittaciformes
Falco peregrinus Falconidae Falconiformes
Cariama cristata Cariamidae Cariamiformes
Picoides pubescens Picidae Piciformes
Merops nubicus Meropidae Coraciiformes
Buceros rhinoceros Bucerotidae Bucerotiformes
Apaloderma vittatum Trogonidae Trogoniformes
Leptosomus discolor Leptosomidae Leptosomiformes
Colius striatus Coliidae Coliiformes
Haliaeetus albicilla Accipitridae Accipitriformes
Haliaeetus leucocephalus Accipitridae Accipitriformes
Cathartes aura Cathartidae Cathartiformes
Tyto alba Tytonidae Strigiformes
Egretta garzetta Ardeidae Pelecaniformes
Pelecanus crispus Pelecanidae Pelecaniformes
Nipponia nippon Threskiornithidae Pelecaniformes
Phalacrocorax carbo Phalacrocoracidae Suliformes
Aptenodytes forsteri Spheniscidae Sphenisciformes
Pygoscelis adeliae Spheniscidae Sphenisciformes
Fulmarus glacialis Procellariidae Procellariiformes
Gavia stellata Gaviidae Gaviiformes
Eurypyga helias Eurypygidae Eurypygiformes
Phaethon lepturus Phaethontidae Phaethontiformes
Charadrius vociferus Charadriidae Charadriiformes
Balearica regulorum Gruidae Gruiformes
Opisthocomus hoazin Opisthocomidae Opisthocomiformes
Calypte anna Trochilidae Caprimulgiformes
Chaetura pelagica Apodidae Caprimulgiformes
Antrostomus carolinensis Caprimulgidae Caprimulgiformes
Tauraco erythrolophus Musophagidae Musophagiformes
Cuculus canorus Cuculidae Cuculiformes
Chlamydotis macqueenii Otididae Otidiformes
Mesitornis unicolor Mesitornithidae Mesitornithiformes
Pterocles gutturalis Pteroclididae Pterocliformes
Columba livia Columbidae Columbiformes
Phoenicopterus ruber Phoenicopteridae Phoenicopteriformes
Podiceps cristatus Podicipedidae Podicipediformes
Gallus gallus Phasianidae Galliformes
Meleagris gallopavo Phasianidae Galliformes
Anas platyrhynchos Anatidae Anseriformes
Tinamus guttatus Tinamidae Struthioniformes
Struthio camelus Struthionidae Struthioniformes
Alligator mississippiensis Alligatoridae Crocodilians
Alligator sinensis Alligatoridae Crocodilians
C
Geospiza fortis
Taeniopygia guttata
Corvus brachyrhynchos
Manacus vitellinus
Acanthisitta chloris
Melopsittacus undulatus
Nestor notabilis
Falco peregrinus
Cariama cristata
Picoides pubescens
Merops nubicus
Buceros rhinoceros
Apaloderma vittatum
Leptosomus discolor
Colius striatus
Tyto alba
Haliaeetus albicilla
Haliaeetus leucocephalus
Cathartes aura
Charadrius vociferus
Egretta garzetta
Pelecanus crispus
Nipponia nippon
Phalacrocorax carbo
Aptenodytes forsteri
Pygoscelis adeliae
Fulmarus glacialis
Gavia stellata
Eurypyga helias
Phaethon lepturus
Calypte anna
Chaetura pelagica
Antrostomus carolinensis
Opisthocomus hoazin
Chlamydotis macqueenii
Cuculus canorus
Tauraco erythrolophus
Mesitornis unicolor
Pterocles gutturalis
Columba livia
Balearica regulorum
Phoenicopterus ruber
Podiceps cristatus
Gallus gallus
Meleagris gallopavo
Anas platyrhynchos
Tinamus guttatus
Struthio camelus
Alligator mississippiensis
Alligator sinensis
D
Geospiza fortis Thraupidae Passeriformes
Taeniopygia guttata Estrildidae Passeriformes
Corvus brachyrhynchos Corvidae Passeriformes
Manacus vitellinus Pipridae Passeriformes
Acanthisitta chloris Acanthisittidae Passeriformes
Melopsittacus undulatus Psittacidae Psittaciformes
Nestor notabilis Strigopidae Psittaciformes
Falco peregrinus Falconidae Falconiformes
Cariama cristata Cariamidae Cariamiformes
Picoides pubescens Picidae Piciformes
Merops nubicus Meropidae Coraciiformes
Buceros rhinoceros Bucerotidae Bucerotiformes
Apaloderma vittatum Trogonidae Trogoniformes
Leptosomus discolor Leptosomidae Leptosomiformes
Colius striatus Coliidae Coliiformes
Tyto alba Tytonidae Strigiformes
Haliaeetus albicilla Accipitridae Accipitriformes
Haliaeetus leucocephalus Accipitridae Accipitriformes
Cathartes aura Cathartidae Cathartiformes
Egretta garzetta Ardeidae Pelecaniformes
Pelecanus crispus Pelecanidae Pelecaniformes
Nipponia nippon Threskiornithidae Pelecaniformes
Phalacrocorax carbo Phalacrocoracidae Suliformes
Aptenodytes forsteri Spheniscidae Sphenisciformes
Pygoscelis adeliae Spheniscidae Sphenisciformes
Fulmarus glacialis Procellariidae Procellariiformes
Gavia stellata Gaviidae Gaviiformes
Eurypyga helias Eurypygidae Eurypygiformes
Phaethon lepturus Phaethontidae Phaethontiformes
Calypte anna Trochilidae Caprimulgiformes
Chaetura pelagica Apodidae Caprimulgiformes
Antrostomus carolinensis Caprimulgidae Caprimulgiformes
Opisthocomus hoazin Opisthocomidae Opisthocomiformes
Mesitornis unicolor Mesitornithidae Mesitornithiformes
Pterocles gutturalis Pteroclididae Pterocliformes
Columba livia Columbidae Columbiformes
Cuculus canorus Cuculidae Cuculiformes
Chlamydotis macqueenii Otididae Otidiformes
Tauraco erythrolophus Musophagidae Musophagiformes
Charadrius vociferus Charadriidae Charadriiformes
Balearica regulorum Gruidae Gruiformes
Phoenicopterus ruber Phoenicopteridae Phoenicopteriformes
Podiceps cristatus Podicipedidae Podicipediformes
Gallus gallus Phasianidae Galliformes
Meleagris gallopavo Phasianidae Galliformes
Anas platyrhynchos Anatidae Anseriformes
Tinamus guttatus Tinamidae Struthioniformes
Struthio camelus Struthionidae Struthioniformes
Alligator mississippiensis Alligatoridae Crocodilians
Alligator sinensis Alligatoridae Crocodilians

Fig. S5

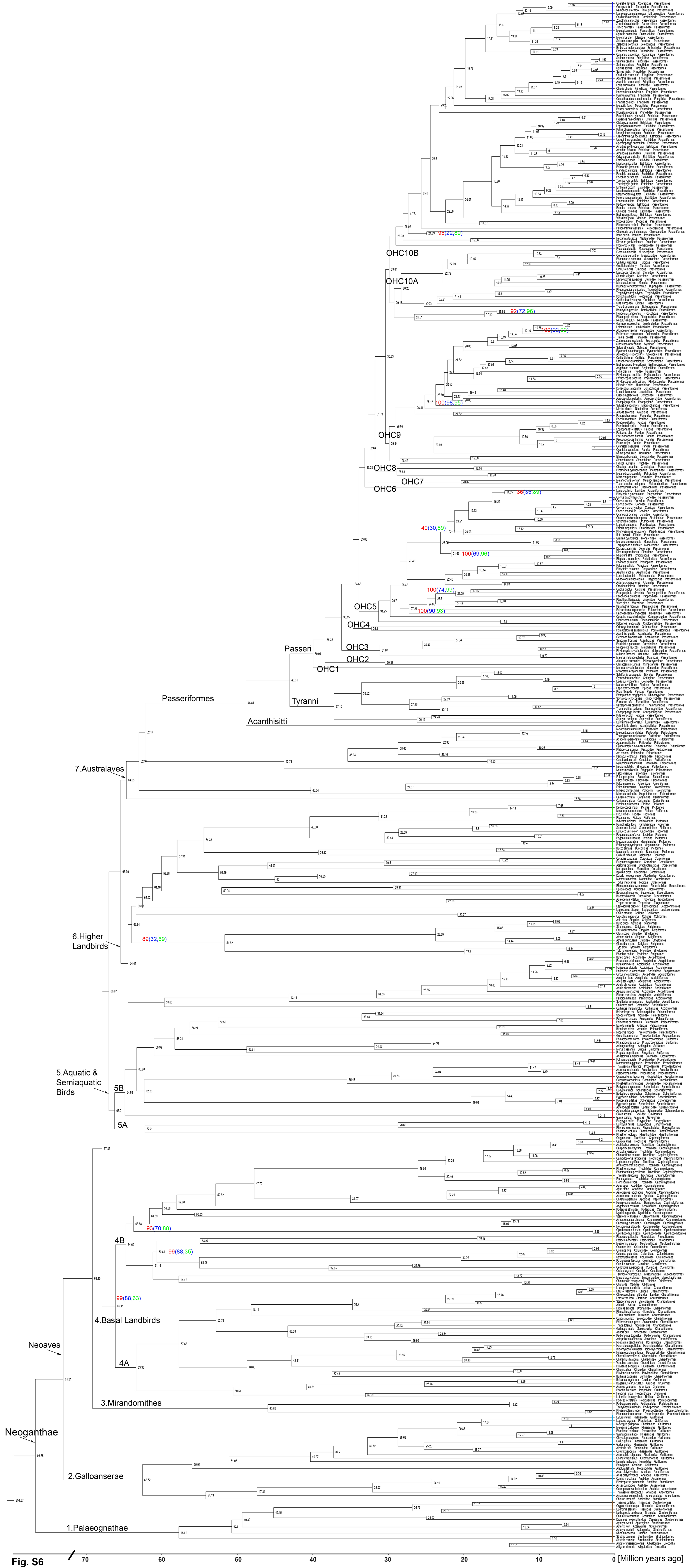

OHC10B
OHC10A
OHC9
OHC8
OHC7
OHC6
OHC5
OHC4
OHC3
OHC2
OHC1
Passeri
Tyranni
Passeriformes
Acanthisitti
7.Australaves
6.Higher
Landbirds
5.Aquatic &
Semiaquatic
Birds
5B
5A
4B
4.Basal Landbirds
4A
3.Mirandornithes
Neoaves
Neoganthae
2.Galloanserae
1.Palaeognathae
95(22,89)
92(72,98)
100(92,99)
100(98,95)
36(35,89)
40(30,89)
100(69,96)
100(74,99)
100(90,93)
89(32,69)
93(70,88)
99(88,35)
99(88,63)
70
60
50
40
30
20
10
0
[Million years ago]

Fig. S6

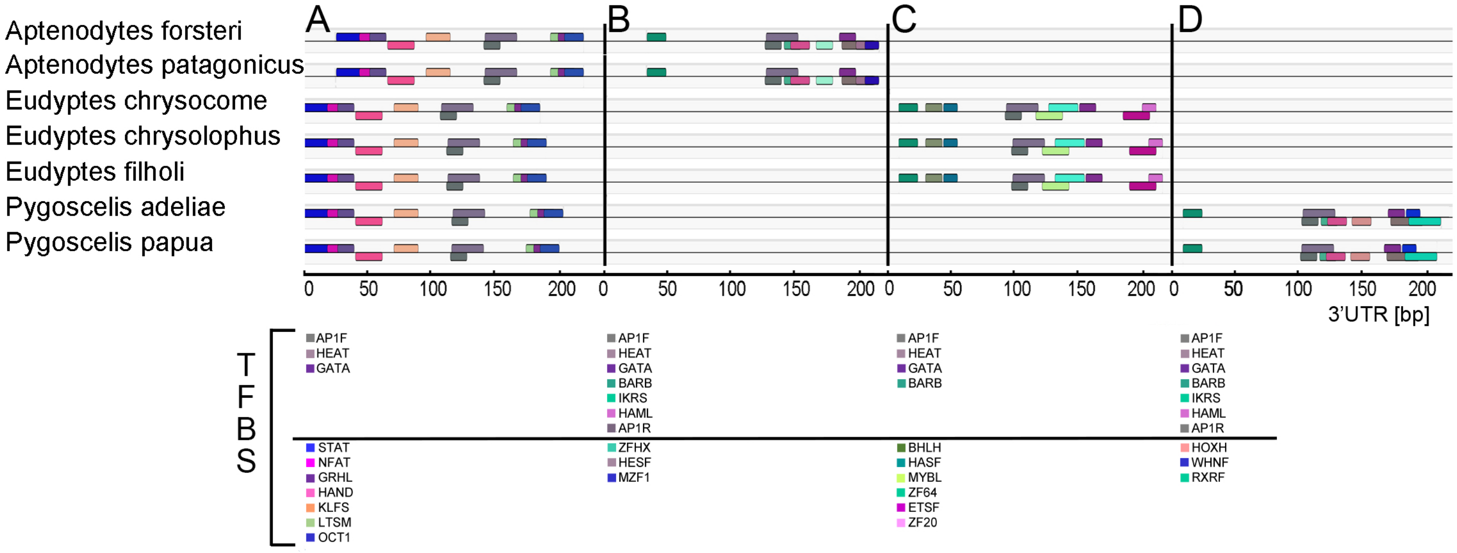
Aptenodytes forsteri
Aptenodytes patagonicus
Eudyptes chrysocome
Eudyptes chrysolophus
Eudyptes filholi
Pygoscelis adeliae
Pygoscelis papua
A
B
C
D
0 50 100 150 200
0 50 100 150 200
0 50 100 150 200
0 50 100 150 200
3'UTR [bp]
TFBS
AP1F
HEAT
GATA
STAT
NFAT
GRHL
HAND
KLFS
LTSM
OCT1
AP1F
HEAT
GATA
BARB
IKRS
HAML
AP1R
ZFHX
HESF
MZF1
AP1F
HEAT
GATA
BARB
BHLH
HASF
MYBL
ZF64
ETSF
ZF20
AP1F
HEAT
GATA
BARB
IKRS
HAML
AP1R
HOXH
WHNF
RXRF

Fig. S7

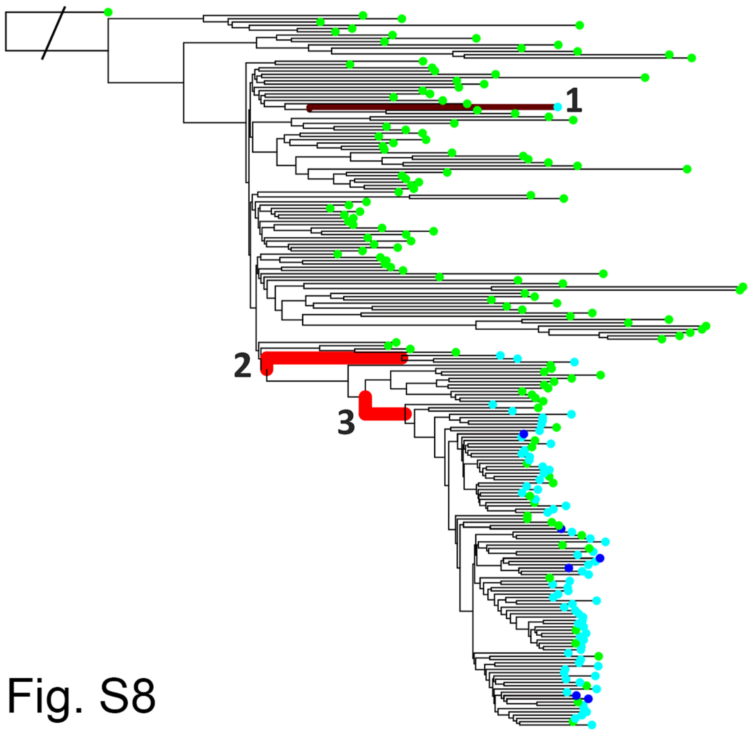

Fig. S8

Tab. S1

Species name	English species name	Family-level taxon	Order-level taxon	Institute	Catalog Number	Country	Year	Data Accesion Numbers
**A. Sequenced Transcriptomes (MPIO)**								
Abroscopus superciliaris	Yellow-belled Warbler	Scotocercidae	Passeriformes	LSUMNS	B-51157	Malaysia		SRR10853103
Acanthiza pusilla	Brown Thornbill	Acanthizidae	Passeriformes	MPIO		missing	2010	SRR10853102
Accipiter nisus	Eurasian Sparrowhawk	Accipitridae	Accipitriformes	MPIO		captive	2011	SRR10852991
Acrocephalus palustris	Marsh Warbler	Acrocephalidae	Passeriformes	CGN		Germany	2015	SRR10852880
Actophilornis africanus	African Jacana	Jacanidae	Charadriiformes	THH		captive	2016	SRR10852854
Aegithalos caudatus	Long-tailed Bushtit	Aegithalidae	Passeriformes	MPIO		Germany	2016	SRR10852843
Aegotheles cristatus	Australian Owlet-Nightjar	Aegothelidae	Caprimulgiformes	ANWC	B53592	Australia		SRR10852832
Aegithina tiphia	Common Iora	Aegithinidae	Passeriformes	CGN		Indonesia	2004	SRR10852821
Agapornis fischeri	Fischer's Lovebird	Psittacidae	Psittaciformes	MPIO		captive	2015	SRR10852810
Agapornis personatus	Yellow-collared Lovebird	Psittacidae	Psittaciformes	MPIO		captive	2015	SRR10852799
Nectarinia tacazze	Tacazze Sunbird	Nectariniidae	Passeriformes	WZS		captive	2015	SRR10853101
Ailuroedus buccoides	White-eared Catbird	Ptilonorhynchidae	Passeriformes	WZS		captive	2015	SRR10853090
Alauda arvensis	Eurasian Skylark	Alaudidae	Passeriformes	MPIO		Germany	2013	SRR10853079
Alcippe morrisonia	Grey-cheeked Fulvetta	Pellorneidae	Passeriformes	CGN		Taiwan	2018	SRR10853068
Alectura lathami	Australian Brushturkey	Megapodidae	Galliformes	VPW		captive	2016	SRR10853057
Alle alle	Little Auk	Alcidae	Charadriiformes	UGP		Poland	2012	SRR10853046
Amandava amandava	Red Avadavat	Estrildidae	Passeriformes	MPIO		captive	2015	*SRR10853035*
Amadina erythrocephala	Red-headed Finch	Estrildidae	Passeriformes	MPIO		captive	2015	SRR10853024
Amadina fasciata	Cut-throat Finch	Estrildidae	Passeriformes	MPIO		captive	2015	SRR10853013
Amazilia versicolor	Versicolored Emerald	Trochilidae	Caprimulgiformes	UFPB		Brazil	2011	SRR10853002
Anastomus lamelligerus	African Openbill	Ciconiidae	Pelecaniformes	MfN		captive		SRR10852990
Anhinga anhinga	Anhinga	Anhingidae	Suliformes	CGN		Suriname	2009	SRR10852979
Anseranas semipalmata	Magpie Goose	Anseranatidae	Anseriformes	ZGB		captive	2015	SRR10852968
Anthracothorax nigricollis	Black-throated Mango	Trochilidae	Caprimulgiformes	UFPB		Brazil	2011	SRR10852957
Apteryx mantelli mantelli	North Island Brown Kiwi	Apterygidae	Struthioniformes	ZGB		captive	2015	SRR10852946
Aptenodytes patagonicus	King Penguin	Spheniscidae	Sphenisciformes	UPS		France	2017	SRR10852935
Apus apus	Common Swift	Apodidae	Caprimulgiformes	MPIO		Germany	2013	SRR10852924
Aramus guarauna	Limpkin	Aramidae	Gruiformes	LSUMNS	B-10769	Peru		SRR10852913
Ardenna tenuirostris	Short-tailed Shearwater	Procellariidae	Procellariiformes	PINP		Australia	2015	SRR10852902
Artamus cyanopterus	Dusky Woodswallow	Artamidae	Passeriformes	ANWC	B50232	Australia		SRR10852891
Atelornis pittoides	Blaukopf-Erdracke	Brachypteraciidae	Coraciiformes	ZZ		captive	2018	SRR10852879
Attagis gayi	Rufous-bellied Seedsnipe	Thinocoridae	Charadriiformes	LSUMNS	B-103907	Peru		SRR10852868
Balaeniceps rex	Shoebill	Balaenicipitidae	Pelecaniformes	MfN		captive		SRR10852862
Bombycilla garrulus	Bohemian Waxwing	Bombycillidae	Passeriformes	MPIO		captive	2014	SRR10852861
Buceros bicornis	Great Hornbill	Bucerotidae	Bucerotiformes	WZS		captive	2015	SRR10852860
Bucco tamatia	Spotted Puffbird	Bucconidae	Piciformes	LSUMNS	B-14722	Bolivia		SRR10852859
Bugeranus carunculatus	Wattled Crane	Gruidae	Gruiformes	ZGK		captive	2015	SRR10852858
Buphagus erythrorhynchus	Red-billed Oxpecker	Buphagidae	Passeriformes	THH		captive	2016	SRR10852857
Burhinus capensis	Spotted Thick-knee	Burhinidae	Charadriiformes	ZGK		captive	2015	SRR10852856
Butorides striata	Striated Heron	Ardeidae	Pelecaniformes	UFPB		Brazil	2011	SRR10852855
Cacatua ducorpsii	Broad-crested Corella	Cacatuidae	Psittaciformes	MPIO		captive	2014	SRR10852853
Calliphlox amethystina	Amethyst Woodstar	Trochilidae	Caprimulgiformes	UFPB		Brazil	2011	SRR10852852
Calcarius lapponicus	Lapland Longspur	Calcariidae	Passeriformes	CGN		Russia	2017	SRR10852851
Campylopterus largipennis	Grey-breasted Sabrewing	Trochilidae	Caprimulgiformes	UFPB		Brazil	2011	SRR10852850
Caprimulgus inornatus	Plain Nightjar	Caprimulgidae	Caprimulgiformes	CGN		South Sudar	2010	SRR10852849
Carduelis cannabina	Common Linnet	Fringillidae	Passeriformes	MPIO		Germany	2017	SRR10852848
Cardinalis cardinalis	Northern Cardinal	Cardinaldidae	Passeriformes	MPIO		captive	2014	SRR10852847
Cariama cristata	Red-legged Seriema	Cariamidae	Cariamiformes	ZGW		captive	2016	SRR10852846
Casuarius casuarius	Southern Cassowary	Casuariidae	Struthioniformes	ANWC	B29953	Australia		SRR10852845
Cathartes melambrotus	Greater Yellow-headed Vulture	Cathartidae	Cathartiformes	CGN		Suriname	2009	SRR10852844
Centropus superciliosus	White-browed Coucal	Cuculidae	Cuculiformes	MPIO		Tanzania	2001	SRR10852842
Certhia brachydactyla	Short-toed Treecreeper	Certhiidae	Passeriformes	MPIO		Germany	2014	SRR10852841
Cereopsis novaehollandiae	Cape Barren Goose	Anatidae	Anseriformes	CLB		captive	2016	SRR10852840
Cettia diphone	Japanese Bush Warbler	Cettidae	Passeriformes	UWBM	47251	Russia	1993	SRR10852839
Ispidina picta	African Pygmy Kingfisher	Alcedinidae	Coraciiformes	CKC		captive	2011	SRR10852838
Chaetops aurantius	Drakensburg Rockjumper	Chaetopidae	Passeriformes	UWBM	52973	South Africa	1994	SRR10852837
Charadrius hiaticula	Common Ringed Plover	Charadriidae	Charadriiformes	ZGK		captive	2015	SRR10852836
Chauna torquata	Southern Screamer	Anhimidae	Anseriformes	TPB		captive	2016	SRR10852835
Chionis albus	Snowy Sheathbill	Chionidae	Charadriiformes	CGN		Argentina	1997	SRR10852834
Chloris chloris	European Greenfinch	Fringillidae	Passeriformes	MPIO		Germany	2017	SRR10852833
Chloropsis cochinchinensis	Blue-winged Leafbird	Chloropseidae	Passeriformes	MPIO		captive	2014	SRR10852831
Chloebia gouldiae	Gouldian Finch	Estrildidae	Passeriformes	MPIO		captive	2015	SRR10852830
Chlorostilbon notatus	Blue-chinned sapphire	Trochilidae	Caprimulgiformes	UFPB		Brazil	2011	SRR10852829
Chroicocephalus ridibundus	Common Black-headed Gull	Laridae	Charadriiformes	MPIO		captive	2014	SRR10852828
Cinclus cinclus	White-throated Dipper	Cinclidae	Passeriformes	AI		captive	2016	SRR10852827
Cinclosoma clarum	Chestnut-backed Quail-thrush	Cinclosomatidae	Passeriformes	ANWC	B51838	Australia		SRR10852826
Cisticola galactotes	Rufous-winged Cisticola	Cisticolidae	Passeriformes	MPIO		Tanzania	2001	SRR10852825
Climacteris picumnus	Brown Treecreeper	Climacteridae	Passeriformes	ANWC	B49917	Australia		SRR10852824
Clytospiza monteiri	Brown Twinspot	Estrildidae	Passeriformes	MPIO		captive	2015	SRR10852823
Cnemophilus loriae	Velvet Satinbird	Cnemophilidae	Passeriformes	MVM	Z43659	Papua NG		SRR10852822

Coccothraustes coccothraustes	Hawfinch	Fringillidae	Passeriformes	MPIO		Germany	2017	SRR10852820
Coereba flaveola	Bananaquit	Coerebidae	Passeriformes	CGN		Suriname	2009	SRR10852819
Columba palumbus	Common Wood Pigeon	Columbidae	Columbiformes	MPIO		captive	2015	SRR10852818
Colinus virginianus	Northern Bobwhite	Odontophoridae	Galliformes	MPIO		captive	2014	SRR10852817
Conopophaga lineata	Rufous Gnateater	Conopophagidae	Passeriformes	UFPB		Brazil	2011	SRR10852816
Coracias caudatus	Lilac-breasted Roller	Coraciidae	Coraciiformes	ZGK		captive	2015	SRR10852815
Corcorax melanorhamphos	White-winged Chough	Struthideidae	Passeriformes	ANWC	B49971	Australia		SRR10852814
Corvus monedula	Western Jackdaw	Corvidae	Passeriformes	MPIO		captive	2015	SRR10852813
Coracina novaehollandiae	Black-faced Cuckooshrike	Campephagidae	Passeriformes	ANWC	B55082	Australia		SRR10852812
Cracticus tibicen	Australian Magpie	Artamidae	Passeriformes	ANWC	B51356	Australia		SRR10852811
Crotophaga ani	Smooth-billed Ani	Cuculidae	Cuculiformes	UFPB		Brazil	2011	SRR10852809
Crypturellus tataupa	Tataupa Tinamou	Tinamidae	Struthioniformes	MPIO		captive	2016	SRR10852808
Cyanoramphus novaezelandiae	Red-fronted Parakeet	Psittacidae	Psittaciformes	MPIO		captive	2015	SRR10852807
Dacelo novaeguineae	Laughing Kookaburra	Alcedinidae	Coraciiformes	ZGK		captive	2015	SRR10852806
Daphoenositta chrysoptera	Varied Sittella	Neosittidae	Passeriformes	ANWC	B43320	Australia		SRR10852805
Dendrocopos major	Great Spotted Woodpecker	Picidae	Piciformes	MPIO		Germany	2017	SRR10852804
Dicrurus adsimilis	Fork-tailed Drongo	Dicruridae	Passeriformes	MPIO		South Africa	2001	SRR10852803
Dicaeum geelvinkianum	Red-capped Flowerpecker	Dicaeidae	Passeriformes	ANWC	B56396	Australia		SRR10852802
Dicrurus paradiseus	Greater Racket-tailed Drongo	Dicruridae	Passeriformes	CGN		Indonesia	2018	SRR10852801
Donacobius atricapillus	Black-capped Donacobius	Donacobiidae	Passeriformes	LSUMNS	B-37871	Bolivia		SRR10852800
Dromas ardeola	Crab-plover	Dromadidae	Charadriiformes	CGN		Tanzania	2019	SRR10852798
Dromaius novaehollandiae	Emu	Casuariidae	Struthioniformes	ANWC	B44264	Australia		SRR10852797
Elminia albonotata	White-tailed Crested-Flycatcher	Stenostiridae	Passeriformes	CGN		Kenya	2017	SRR10852796
Emberiza citrinella	Yellowhammer	Emberizidae	Passeriformes	MPIO		Germany	2017	SRR10852795
Emblema pictum	Painted Finch	Estrildidae	Passeriformes	MPIO		captive	2015	SRR10852794
Erythrura psittacea	Red-throated Parrot-Finch	Estrildidae	Passeriformes	MPIO		captive	2015	SRR10852793
Estrilda melpoda	Orange-cheeked Waxbill	Estrildidae	Passeriformes	MPIO		captive	2015	SRR10852792
Eubucco versicolor	Versicolored Barbet	Capitonidae	Piciformes	UWBM	77164	Bolivia		SRR10852791
Eudromia elegans	Elegant Crested Tinamou	Tinamidae	Struthioniformes	MPIO		captive	2016	SRR10852790
Euodice cantans	African Silverbill	Estrildidae	Passeriformes	MPIO		captive	2015	SRR10852789
Eurystomus glaucurus	Broad-billed Roller	Coraciidae	Coraciiformes	CKC		captive	2011	SRR10853100
Eurypyga helias	Sunbittern	Eurypygidae	Eurypygiformes	UFPB		Brazil	2011	SRR10853099
Eurylaimus ochromalus	Black-and-yellow Broadbill	Eurylaimidae	Passeriformes	LSUMNS	B-50329	Malaysia		SRR10853098
Euschistospiza dybowskii	Dybowski's Twinspot	Estrildidae	Passeriformes	CGN		South Sudan	2010	SRR10853097
Falculea palliata	Sickle-billed Vanga	Vangidae	Passeriformes	VPW		captive	2016	SRR10853096
Falco sparverius	American Kestrel	Falconidae	Falconiformes	MNHNC		captive		SRR10853095
Florisuga fuscus	Black Jacobin	Trochilidae	Caprimulgiformes	UFPB		Brazil	2011	SRR10853094
Florisuga mellivora	White-necked Jacobin	Trochilidae	Caprimulgiformes	UFPB		Brazil	2011	SRR10853093
Fregata magnificens	Magnificent Frigatebird	Fregatidae	Suliformes	UWBM	73908	Panama		SRR10853092
Fringilla coelebs	Common Chaffinch	Fringillidae	Passeriformes	MPIO		Germany	2017	SRR10853091
Furnarius rufus	Rufous Hornero	Furnariidae	Passeriformes	UFPB		Brazil	2011	SRR10853089
Galbula ruficauda	Rufous-tailed Jacamar	Galbulidae	Piciformes	LSUMNS	B-18480	Bolivia		SRR10853088
Garrulax leucolophus	White-crested Laughingthrush	Leiothrichidae	Passeriformes	MPIO		captive	2014	SRR10853087
Gavia stellata	Red-throated Loon	Gaviidae	Gaviiformes	UWBM	44653	Russia		SRR10853086
Geronticus eremita	Northern Bald Ibis	Threskiornithidae	Pelecaniformes	THM		captive	2016	SRR10853085
Gerygone flavolateralis	Fan-tailed Gerygone	Acanthizidae	Passeriformes	TUM		New Caledo	2010	SRR10853084
Glaucidium nanum	Austral Pygmy Owl	Strigidae	Strigiformes	MNHNC		Chile		SRR10853083
Grallina cyanoleuca	Magpielark	Monarchidae	Passeriformes	ANWC	B55153	Australia		SRR10853082
Gymnoderus foetidus	Bare-necked Fruitcrow	Cotingidae	Passeriformes	CGN		Suriname	2009	SRR10853081
Haematopus palliatus	American Oystercatcher	Haematopodidae	Charadriiformes	UFPB		Brazil	2011	SRR10853080
Heliornis fulica	Sungrebe	Heliornithidae	Gruiformes	CGN		Suriname	2009	SRR10853078
Hemiprocne mystacea	Moustached Treeswift	Hemiprocnidae	Caprimulgiformes	ANWC	B56259	Australia		SRR10853077
Heteromunia pectoralis	Pictorella Munia	Estrildidae	Passeriformes	ANWC	B55173	Australia		SRR10853076
Himantopus himantopus	Black-winged Stilt	Recurvirostridae	Charadriiformes	WZS		captive	2015	SRR10853075
Hirundo rustica	Barn Swallow	Hirundinidae	Passeriformes	MPIO		Germany	2017	SRR10853074
Hylia prasina	Green Hylia	Hyliidae	Passeriformes	CGN		South Sudan	2010	SRR10853073
Hypocolius ampelinus	Hypocolius	Hypocoliidae	Passeriformes	ZH		captive	2016	SRR10853072
Hypargos niveoguttatus	Red-throated Twinspot	Estrildidae	Passeriformes	MPIO		captive	2015	SRR10853071
Ifrita kowaldi	Blue-capped Ifrit	Ifritidae	Passeriformes	MVM	Z43109	Papua NG		SRR10853070
Indicator indicator	Greater Honeyguide	Indicatoridae	Piciformes	LSUMNS	B-39498	Ghana		SRR10853069
Irena puella	Asian Fairy-bluebird	Irenidae	Passeriformes	WZS		captive	2016	SRR10853067
Lagopus lagopus	Willow Ptarmigan	Phasianidae	Galliformes	CLB		captive	2016	SRR10853066
Lagonosticta rubricata	African Firefinch	Estrildidae	Passeriformes	MPIO		captive	2015	SRR10853065
Lamprospiza melanoleuca	Red-billed Pied Tanager	Mitrospingidae	Passeriformes	LSUMNS	B-40857	Peru		SRR10853064
Lanius collurio	Red-backed Shrike	Laniidae	Passeriformes	MPIO		Germany	2017	SRR10853063
Laniarius funebris	Slate-colored Boubou	Malaconotidae	Passeriformes	MPIO		captive	2014	SRR10853062
Larus crassirostris	Black-tailed Gull	Laridae	Charadriiformes	TPB		captive	2016	SRR10853061
Larosterna inca	Inca Tern	Sternidae	Charadriiformes	CLB		captive	2016	SRR10853060
Laterallus leucopyrrhus	Red-and-white Crake	Rallidae	Gruiformes	MPIO		captive	2015	SRR10853059
Leiothrix lutea	Red-billed Leiothrix	Leiothrichidae	Passeriformes	MPIO		captive	2014	SRR10853058
Leptosomus discolor	Cuckoo Roller	Leptosomidae	Leptosomiformes	VPW		captive	2016	SRR10853056
Leucopsar rothschildi	Bali Myna	Sturnidae	Passeriformes	ZGK		captive	2015	SRR10853055
Lipaugus vociferans	Screaming Piha	Cotingidae	Passeriformes	UFPB		Brazil	2011	SRR10853054
Locustella naevia	Common Grasshopper Warbler	Locustellidae	Passeriformes	MfN		Germany		*SRR10853053*
Lonchura striata	White-rumped Munia	Estrildidae	Passeriformes	MPIO		captive	2015	SRR10853052
Lophophanes cristatus	European Crested Tit	Paridae	Passeriformes	MPIO		Germany	2017	SRR10853051
Lophornis magnificus	Frilled Coquette	Trochilidae	Caprimulgiformes	UFPB		Brazil	2011	SRR10853050

Lophorina superba	Superb Bird-of-Paradise	Paradisaeidae	Passeriformes	CGN		Indonesia	2018	SRR10853049
Malurus melanocephalus	Red-backed Fairywren	Maluridae	Passeriformes	WSU		Australia	2012	SRR10853048
Malacoptila panamensis	White-whiskered Puffbird	Bucconidae	Piciformes	UWBM	77010	Panama		SRR10853047
Mandingoa nitidula	Green Twinspot	Estrildidae	Passeriformes	LSUMNS	B-45139	Ghana		SRR10853045
Manacus vitellinus	Golden-collared Manakin	Pipridae	Passeriformes	UV		Panama	2014	SRR10853044
Megalaima asiatica	Blue-throated Barbet	Megalaimidae	Piciformes	WZS		captive	2015	SRR10853043
Melanerpes cruentatus	Yellow-tufted Woodpecker	Picidae	Piciformes	UFPB		Brazil	2011	SRR10853042
Melanodryas cucullata	Hooded Robin	Petroicidae	Passeriformes	ANWC	B32048	Australia		SRR10853041
Melanocharis versteri	Fan-tailed Berrypecker	Melanocharitidae	Passeriformes	ANWC	B-52506	Australia		SRR10853040
Menura novaehollandiae	Superb Lyrebird	Menuridae	Passeriformes	ANWC	B46196	Australia		SRR10853039
Microeca papuana	Canary Flyrobin	Petroicidae	Passeriformes	CGN		Indonesia	2018	SRR10853038
Micrastur ruficollis	Barred Forest Falcon	Herpetotheriane	Falconiformes	LSUMNS	B-14605	Bolivia		SRR10853037
Milvago chimachima	Yellow-headed Caracara	Polyborini	Falconiformes	MNHNC		captive		SRR10853036
Mimus saturninus	Chalk-browed Mockingbird	Mimidae	Passeriformes	UFPB		Brazil	2011	SRR10853034
Molothrus ater	Brown-headed Cowbird	Icteridae	Passeriformes	UWBM	118374	USA		SRR10853033
Momotus momota	Blue-crowned Motmot	Momotidae	Coraciiformes	CGN		Suriname	2009	SRR10853032
Monarcha melanopsis	Australian Logrunner	Orthonychidae	Passeriformes	ANWC	B46917	Australia		SRR10853031
Morus bassanus	Northern Gannet	Sulidae	Suliformes	WZS		captive	2015	SRR10853030
Motacilla flava	Western Yellow Wagtail	Motacillidae	Passeriformes	MPIO		Germany	2017	SRR10853029
Musophaga violacea	Violet Turaco	Musophagidae	Musophagiformes	ZGK		captive	2015	SRR10853028
Myiozetetes cayanensis	Rusty-margined Flycatcher	Tyrannidae	Passeriformes	UFPB		Brazil	2011	SRR10853027
Neochmia temporalis	Red-browed Finch	Estrildidae	Passeriformes	MPIO		captive	2015	*SRR10853026*
Nestor meridionalis	New Zealand Kaka	Strigopidae	Psittaciformes	WZS		captive	2015	SRR10853025
Nicator chloris	Western Nicator	Nicatoridae	Passeriformes	LSUMNS	B-45344	Ghana		SRR10853023
Nigrita canicapilla	Grey-headed Nigrita	Estrildidae	Passeriformes	CKC		captive	2011	SRR10853022
Numida meleagris	Helmeted Guineafowl	Numididae	Galliformes	MPIO		captive	2011	SRR10853021
Nyctidromus albicollis	Pauraque	Caprimulgidae	Caprimulgiformes	UFPB		Brazil	2011	SRR10853020
Nyctibius grandis	Great Potoo	Nyctibiidae	Caprimulgiformes	LSUMNS	B-15415	Bolivia		SRR10853019
Nymphicus hollandicus	Cockatiel	Cacatuidae	Psittaciformes	MPIO		captive	2014	SRR10853018
Oceanodroma leucorhoa	Leach's Storm Petrel	Hydrobatidae	Procellariiformes	UWBM	60497	missing		SRR10853017
Oceanites oceanicus	Wilson's Storm Petrel	Oceanitidae	Procellariiformes	UG		Argentina	2014	SRR10853016
Opisthocomus hoazin	Hoatzin	Opisthocomidae	Opisthocomiformes	CGN		Suriname	2009	SRR10853015
Oriolus oriolus	Eurasian Golden Oriole	Oriolidae	Passeriformes	MPIO		Germany	2017	SRR10853014
Ortygospiza atricollis	Black-faced Quail-Finch	Estrildidae	Passeriformes	MPIO		captive	2015	SRR10853012
Orthonyx temminckii	Black-faced Monarch	Monarchidae	Passeriformes	ANWC	B47270	Australia		SRR10853011
Otis tarda	Great Bustard	Otididae	Otidiformes	TPB		captive	2016	SRR10853010
Pachycephala rufiventris	Rufous Whistler	Pachycephalidae	Passeriformes	ANWC	B29590	Australia		SRR10853009
Padda oryzivora	Java Sparrow	Estrildidae	Passeriformes	MPIO		captive	2015	SRR10853008
Panurus biarmicus	Bearded Reedling	Panuridae	Passeriformes	MPIO		Germany	2017	SRR10853007
Parmoptila jamesoni	Jameson's Antpecker	Estrildidae	Passeriformes	LSUMNS	B-45141	Ghana		SRR10853006
Parus major	Great Tit	Paridae	Passeriformes	MPIO		Germany	2017	SRR10853005
Paramythia montium olivacea	Crested Berrypecker	Paramythiidae	Passeriformes	CGN		Indonesia	2018	SRR10853004
Pardalotus punctatus	Spotted Pardalote	Pardalotidae	Passeriformes	ANWC	B49956	Australia		SRR10853003
Parabuteo unicinctus	Harris's Hawk	Accipitridae	Accipitriformes	MNHNC		captive		SRR10853001
Passer domesticus	House Sparrow	Passeridae	Passeriformes	MPIO		Germany	2017	SRR10853000
Pauxi pauxi	Northern Helmeted Curassow	Cracidae	Galliformes	MPIO		captive	2015	SRR10852999
Pedionomus torquatus	Plains-wanderer	Pedionomidae	Charadriiformes	ANWC	B44673	Australia		SRR10852998
Pellorneum capistratum	Black-capped Babbler	Pellorneidae	Passeriformes	LSUMNS	B-46995	Malaysia		SRR10852997
Pelecanus onocrotalus	Great White Pelican	Pelecanidae	Pelecaniformes	WZS		captive	2015	SRR10852996
Periparus ater	Coal Tit	Paridae	Passeriformes	MPIO		Germany	2017	SRR10852995
Peucedramus taeniatus	Olive Warbler	Peucedramidae	Passeriformes	UWBM	77814	USA		SRR10852994
Phalacrocorax carbo	Great Cormorant	Phalacrocoracidae	Suliformes	MPIO		Germany	2017	SRR10852993
Phaethon lepturus	White-tailed Tropicbird	Phaethontidae	Phaethontiformes	ULR		France	2010	SRR10852992
Phainopepla nitens	Phainopepla	Ptilogonatidae	Passeriformes	UWBM	117656	USA		SRR10852989
Phaethornis ruber	Reddish Hermit	Trochilidae	Caprimulgiformes	UFPB		Brazil	2011	SRR10852988
Phaethornis superciliosus	Long-tailed Hermit	Trochilidae	Caprimulgiformes	UFPB		Brazil	2011	SRR10852987
Pheugopedius genibarbis	Moustached Wren	Troglodytidae	Passeriformes	UFPB		Brazil	2011	SRR10852986
Philomachus pugnax	Ruff	Scolopacidae	Charadriiformes	MPIO		captive	2014	SRR10852985
Phodilus badius	Oriental Bay Owl	Tytonidae	Strigiformes	TPB		captive	2016	SRR10852984
Phoebastria immutabilis	Laysan Albatross	Diomedeidae	Procellariiformes	UWBM	65657	USA		SRR10852983
Phonygammus keraudrenii	Trumpet Manucode	Paradisaeidae	Passeriformes	ANWC	B43000	Australia		SRR10852982
Phoenicurus ochruros	Black Redstart	Muscicapidae	Passeriformes	MPIO		Germany	2010	SRR10852981
Phoenicopterus roseus	Greater Flamingo	Phoenicopteridae	Phoenicopteriform	WZS		captive	2016	SRR10852980
Phylidonyris novaehollandiae	New Holland Honeyeater	Meliphagidae	Passeriformes	MPIO		missing	2010	SRR10852978
Phylloscopus umbrovirens	Brown Woodland Warbler	Phylloscopidae	Passeriformes	CGN		South Sudar	2010	SRR10852977
Picathartes gymnocephalus	White-necked Picathartes	Picathartidae	Passeriformes	LSUMNS	B-19213	captive		SRR10852976
Picus viridis	European Green Woodpecker	Picidae	Piciformes	MPIO		Germany	2017	SRR10852975
Pitta versicolor	Noisy Pitta	Pittidae	Passeriformes	ANWC	B31463	Australia		SRR10852974
Platysteira castanea	Chestnut Wattle-eye	Platysteiridae	Passeriformes	CGN		South Sudar	2010	SRR10852973
Platylophus galericulatus	Crested Jay	Platylophidae	Passeriformes	LSUMNS	B-38663	Malaysia		SRR10852972
Plectropterus gambensis	Spur-winged Goose	Anatidae	Anseriformes	CLB		captive	2016	SRR10852971
Ploceus bicolor	Dark-backed Weaver	Ploceidae	Passeriformes	MPIO		South Africa	2011	SRR10852970
Plocepasser mahali	White-browed Sparrow-Weaver	Ploceidae	Passeriformes	MPIO		South Africa	2017	SRR10852969
Pluvianus aegyptius	Egyptian Plover	Pluvianidae	Charadriiformes	CLB		captive	2016	SRR10852967
Podiceps nigricollis	Black-necked Grebe	Podicipedidae	Podicipediformes	WZS		captive	2015	SRR10852966
Podargus strigoides	Tawny Frogmouth	Podargidae	Caprimulgiformes	ANWC	B55784	Australia		SRR10852965
Poephila acuticauda	Violet-eared Waxbill	Estrildidae	Passeriformes	MPIO		captive	2015	SRR10852964

Poecile montanus	Willow Tit	Paridae	Passeriformes	MPIO		Germany	2017	SRR10852963
Poecile palustris	Marsh Tit	Paridae	Passeriformes	MPIO		Germany	2017	SRR10852962
Poephila personata	Masked Finch	Estrildidae	Passeriformes	MPIO		captive	2015	SRR10852961
Pogoniulus atroflavus	Red-rumped Tinkerbird	Lybiidae	Piciformes	MPIO		captive	2016	SRR10852960
Pogoniulus bilineatus	Yellow-rumped Tinkerbird	Lybiidae	Piciformes	CGN		South Sudar	2010	SRR10852959
Polioptila albiloris	White-lored Gnatcatcher	Polioptilidae	Passeriformes	LSUMNS	B60746	Honduras		SRR10852958
Pomatostomus superciliosus	White-browed Babbler	Pomatostomidae	Passeriformes	ANWC	B29279	Australia		SRR10852956
Prionops plumatus	White-crested Helmetshrike	Prionopidae	Passeriformes	UWBM	110339	Malawi	2001	SRR10852955
Promerops cafer	Cape Sugarbird	Promeropidae	Passeriformes	MPIO		South Africa	2005	SRR10852954
Prunella modularis	Dunnock	Prunellidae	Passeriformes	MPIO		Germany	2017	SRR10852953
Psittacus erithacus	Grey Parrot	Psittacidae	Psittaciformes	MPIO		captive	2015	SRR10852952
Psilopogon pyrolophus	Fire-tufted Barbet	Megalaimidae	Piciformes	ZGK		captive	2015	SRR10852951
Psophia crepitans	Grey-winged Trumpeter	Psophiidae	Gruiformes	ZGK		captive	2015	SRR10852950
Psophodes olivaceus	Eastern Whipbird	Psophodidae	Passeriformes	ANWC	B43472	Australia		SRR10852949
Pterodroma baraui	Barau's Petrel	Procellariidae	Procellariiformes	ULR		France	2010	SRR10852948
Pteruthius flaviscapis	Pied Shrike-Babbler	Vireonidae	Passeriformes	CGN		Indonesia	2018	SRR10852947
Pteroptochos megapodius	Moustached Turca	Rhinocryptidae	Passeriformes	MNHNC		Chile		SRR10852945
Pterocles orientalis	Black-bellied Sandgrouse	Pteroclididae	Pterocliformes	NARC		UAR	2016	SRR10852944
Ptiloris magnificus	Magnificent Riflebird	Paradisaeidae	Passeriformes	ANWC	B43100	Australia		SRR10852943
Pycnonotus xanthopygos	White-spectacled Bulbul	Pycnonotidae	Passeriformes	WZS		captive	2015	SRR10852942
Pyrrhula pyrrhula	Eurasian Bullfinch	Fringillidae	Passeriformes	MPIO		Germany	2017	SRR10852941
Pytilia phoenicoptera	Red-winged Pytilia	Estrildidae	Passeriformes	MPIO		captive	2015	SRR10852940
Ramphocelus carbo	Silver-beaked Tanager	Thraupidae	Passeriformes	UFPB		Brazil	2011	SRR10852939
Ramphastos toco	Toco Toucan	Ramphastidae	Piciformes	WZS		captive	2015	SRR10852938
Regulus regulus	Goldcrest	Regulidae	Passeriformes	MPIO		Germany	2017	SRR10852937
Remiz pendulinus	Eurasian Penduline Tit	Remizidae	Passeriformes	MPIO		captive	2016	SRR10852936
Rhagologus leucostigma	Mottled Whistler	Rhagologidae	Passeriformes	MVM	Z43546	Papua NG		SRR10852934
Rhea americana	Greater Rhea	Rheidae	Struthioniformes	UFPB		captive	2012	SRR10852933
Rhinoptilus africanus	Double-banded Courser	Glareolidae	Charadriiformes	THH		captive	2016	SRR10852932
Rhipidura atra	Black Fantail	Rhipiduridae	Passeriformes	CGN		Indonesia	2018	SRR10852931
Rhinopomastus cyanomelas	Common Scimitarbill	Phoeniculidae	Bucerotiformes	LSUMNS	B-34251	South Africa		SRR10852930
Rhipidura leucophrys	Willie Wagtail	Rhipiduridae	Passeriformes	ANWC	B29181	Australia		SRR10852929
Rhynochetos jubatus	Kagu	Rhynochetidae	Eurypygiformes	TUM		New Caledo	2010	SRR10852928
Rostratula benghalensis	Greater Painted Snipe	Rostratulidae	Charadriiformes	CKC		captive	2011	SRR10852927
Sagittarius serpentarius	Secretarybird	Sagittariidae	Accipitriformes	TPB		captive	2016	SRR10852926
Sakesphorus canadensis	Black-crested Antshrike	Thamnophilidae	Passeriformes	UFPB		Brazil	2011	SRR10852925
Sapayoa aenigma	Broad-billed Sapayoa	Sapayoidae	Passeriformes	LSUMNS	B-2330	Panama		SRR10852923
Schiffornis veraepacis	Northern Schiffornis	Tityridae	Passeriformes	LSUMNS	B-60681	Honduras		SRR10852922
Scopus umbretta	Hamerkop	Scopidae	Pelecaniformes	TPB		captive	2016	SRR10852921
Scytalopus chocoensis	Choco Tapaculo	Rhinocryptidae	Passeriformes	LSUMNS	B-11960	Ecuador		SRR10852920
Seiurus aurocapillus	Ovenbird	Parulidae	Passeriformes	UWBM	69381	Nicaragua		SRR10852919
Semnornis frantzii	Prong-billed Barbet	Semnornithidae	Piciformes	LSUMNS	B-16016	Costa Rica		SRR10852918
Serinus canaria	Atlantic Canary	Fringillidae	Passeriformes	MPIO		Spain	2001	SRR10852917
Serinus serinus	European Serin	Fringillidae	Passeriformes	MPIO		Germany	2017	SRR10852916
Sitta europaea	Eurasian Nuthatch	Sittidae	Passeriformes	MPIO		Germany	2017	SRR10852915
Spermophaga haematina	Western Bluebill	Estrildidae	Passeriformes	LSUMNS	B-45304	Ghana		SRR10852914
Spizella passerina	Chipping Sparrow	Passerellidae	Passeriformes	UWBM	118304	USA	2014	SRR10852912
Stagonopleura guttata	Diamond Firetail	Estrildidae	Passeriformes	MPIO		captive	2015	SRR10852911
Steatornis caripensis	Oilbird	Steatornithidae	Caprimulgiformes	LSUMNS	B-40597	Peru		SRR10852910
Vidua interjecta	Long-tailed Paradise Whydah	Viduidae	Passeriformes	WZS		captive	2015	SRR10852909
Stenostira scita	Fairy Flycatcher	Stenostiridae	Passeriformes	UWBM	52729	South Africa	1994	SRR10852908
Stercorarius skua	Great Skua	Stercorariidae	Charadriiformes	MfN		Germany		SRR10852907
Struthio camelus	Common Ostrich	Struthionidae	Struthioniformes	MPIO		captive	2012	SRR10852906
Struthidea cinerea	Apostlebird	Struthideidae	Passeriformes	ANWC	B51697	Australia		SRR10852905
Streptopelia risoria	African Collared Dove	Columbidae	Columbiformes	MPIO		captive	2015	SRR10852904
Strix nebulosa	Great Grey Owl	Strigidae	Strigiformes	WZS		captive	2015	SRR10852903
Sylvia atricapilla	Eurasian Blackcap	Sylviidae	Passeriformes	MPIO		Germany	2017	SRR10852901
Sylvietta leucophrys	White-browed Crombec	Macrosphenidae	Passeriformes	CGN		South Sudar	2010	SRR10852900
Tachybaptus ruficollis	Little Grebe	Podicipedidae	Podicipediformes	AI		captive	2016	SRR10852899
Taeniopygia guttata	Zebra Finch	Estrildidae	Passeriformes	MPIO		captive	2015	SRR10852898
Terpsiphone rufiventer	Black-headed Paradise-Flycatcher	Monarchidae	Passeriformes	CKC		captive	2011	SRR10852897
Thalassoica antarctica	Antarctic Petrel	Procellariidae	Procellariiformes	UW		captive		SRR10852896
Thalassornis leuconotus	White-backed Duck	Anatidae	Anseriformes	CLB		captive	2016	SRR10852895
Thamnophilus palliatus	Chestnut-backed Antshrike	Thamnophilidae	Passeriformes	UFPB		Brazil	2011	SRR10852894
Threnetes leucurus	Pale-tailed Barbthroat	Trochilidae	Caprimulgiformes	UFPB		Brazil	2011	SRR10852893
Tichodroma muraria	Wallcreeper	Tichodromidae	Passeriformes	AI		captive	2016	SRR10852892
Timalia pileata	Chestnut-capped Babbler	Timaliidae	Passeriformes	CGN		Indonesia	2018	SRR10852890
Todus mexicanus	Puerto Rican Tody	Todidae	Coraciiformes	LSUMNS	B-16815	Puerto Rico		SRR10852889
Toxorhamphus poliopterus	Slaty-headed Longbill	Melanocharitidae	Passeriformes	CGN		Indonesia	2018	SRR10852888
Trichoglossus moluccanus	Rainbow Lorikeet	Psittacidae	Psittaciformes	MPIO		captive	2015	SRR10852887
Tringa totanus	Common Redshank	Scolopacidae	Charadriiformes	WZS		captive	2015	SRR10852886
Trogon surrucura	Surucua Trogon	Trogonidae	Trogoniformes	UFPB		Brazil	2011	SRR10852885
Troglodytes troglodytes	Winter Wren	Troglodytidae	Passeriformes	MPIO		Germany	2017	SRR10852884
Turnix suscitator	Barred Buttonquail	Turnicidae	Charadriiformes	MPIO		captive	2010	SRR10852883
Upupa epops	Eurasian Hoopoe	Upupidae	Bucerotiformes	WZS		captive	2015	SRR10852882
Uraeginthus bengalus	Red-cheeked Cordon-bleu	Estrildidae	Passeriformes	MPIO		captive	2010	SRR10852881
Uraeginthus cyanocephalus	Blue-capped Cordon-bleu	Estrildidae	Passeriformes	MPIO		captive	2010	SRR10852878

Urocolius macrourus	Blue-naped Mousebird	Coliidae	Coliiformes	ZGB		captive	2015	SRR10852877
Urosphena squameiceps	Asian Stubtail	Scotocercidae	Passeriformes	UWBM	47107	Russia	1993	SRR10852876
Vanellus coronatus	Crowned Lapwing	Charadriidae	Charadriiformes	ZGK		captive	2015	SRR10852875
Vireo gilvus	Warbling Vireo	Vireonidae	Passeriformes	UWBM	117518	USA		SRR10852874
Zeledonia coronata	Wrenthrush	Zeledoniidae	Passeriformes	LSUMNS	B-19938	Costa Rica		SRR10852873
Geokichla dohertyi	Chestnut-backed Thrush	Turdidae	Passeriformes	ZGK		captive	2015	SRR10852872
Zosterops senegalensis	African Yellow White-eye	Zosteropidae	Passeriformes	MPIO		captive	2016	SRR10852871

B. Previously Published Transcriptomes

Acanthis flammea	Common Redpoll	Fringillidae	Passeriformes	SRR1531388
Acanthis hornemanni	Arctic Redpoll	Fringillidae	Passeriformes	SRR1531389
Accipiter virgatus	Besra	Accipitridae	Accipitriformes	SRR3203234
Aegypius monachus	Cinereous Vulture	Accipitridae	Accipitriformes	SRR3203236
Aerodramus fuciphagus	Edible-nest Swiftlet	Apodidae	Caprimulgiformes	SRR4428213
Aerodramus maximus	Black-nest Swiftlet	Apodidae	Caprimulgiformes	SRR4116895
Alectoris rufa	Red-legged Partridge	Phasianidae	Galliformes	SRR2089995
Anas platyrhynchos	Mallard	Anatidae	Anseriformes	SRR1796022
Anser cygnoides	Swan Goose	Anatidae	Anseriformes	SRR1796012
Apteryx owenii	Little Spotted Kiwi	Apterygidae	Struthioniformes	SRR3480320
Apteryx rowi	Okarito Kiwi	Apterygidae	Struthioniformes	SRR3486434
Apus affinis	Little Swift	Apodidae	Caprimulgiformes	SRR4107077
Aquila chrysaetos	Golden Eagle	Accipitridae	Accipitriformes	SRR1818011
Arborophila rufipectus	Sichuan Partridge	Phasianidae	Galliformes	SRR6382407
Archilochus colubris	Ruby-throated Hummingbird	Trochilidae	Caprimulgiformes	SRR6148275
Asio otus	Long-eared Owl	Strigidae	Strigiformes	SRR3203220
Athene noctua	Little Owl	Strigidae	Strigiformes	SRR3203242
Bubo bubo	Eurasian Eagle-Owl	Strigidae	Strigiformes	SRR3203225
Butastur indicus	Grey-faced Buzzard	Accipitridae	Accipitriformes	SRR3203233
Buteo buteo	Common Buzzard	Accipitridae	Accipitriformes	ERR441002
Cairina moschata	Muscovy Duck	Anatidae	Anseriformes	SRR6367503
Calidris pugnax	Ruff	Scolopacidae	Charadriiformes	ERR1018145
Calypte anna	Anna's Hummingbird	Trochilidae	Caprimulgiformes	SRR029422
Catharus ustulatus	Swainson's Thrush	Turdidae	Passeriformes	SRR4340342
Chrysolophus pictus	Golden Pheasant	Phasianidae	Galliformes	SRR3583129
Circus melanoleucos	Pied Harrier	Accipitridae	Accipitriformes	SRR3203217
Columba livia	Common Pigeon	Columbidae	Columbiformes	SRR2094789
Corvus corone	Carrion Crow	Corvidae	Passeriformes	SRR947793
Corvus macrorhynchos	Large-billed Crow	Corvidae	Passeriformes	SRR1023635
Coturnix japonica	Japanese Quail	Phasianidae	Galliformes	SRR1758120
Cyanistes caeruleus	Blue Tit	Paridae	Passeriformes	SRR2040636
Cyanopica cyanus	Azure-winged Magpie	Corvidae	Passeriformes	SRR1917029
Elanus caeruleus	Black-winged Kite	Accipitridae	Accipitriformes	SRR3203227
Emberiza melanocephala	Black-headed Bunting	Emberizidae	Passeriformes	SRR6207197
Eudyptes chrysocome	Rockhopper Penguin	Spheniscidae	Sphenisciformes	SRR1324932
Eudyptes chrysolophus	Macaroni Penguin	Spheniscidae	Sphenisciformes	SRR5253661
Eudyptes filholi	Eastern Rockhopper Penguin	Spheniscidae	Sphenisciformes	SRR1324928
Falco subbuteo	Eurasian Hobby	Falconidae	Falconiformes	SRR3203238
Falco tinnunculus	Common Kestrel	Falconidae	Falconiformes	SRR3203231
Ficedula albicollis	Collared Flycatcher	Muscicapidae	Passeriformes	ERR168848
Gallinago media	Great Snipe	Scolopacidae	Charadriiformes	SRR609950
Gallus gallus	Red Junglefowl	Phasianidae	Galliformes	SRR4040241
Haemorhous mexicanus	House Finch	Fringillidae	Passeriformes	SRR768509
Junco hyemalis	Dark-eyed Junco	Passerellidae	Passeriformes	SRR1759140
Lamprotornis superbus	Superb Starling	Sturnidae	Passeriformes	SRR1565487
Lepidothrix coronata	Blue-crowned Manakin	Pipridae	Passeriformes	SRR3493972
Leucophaeus atricilla	Laughing Gull	Laridae	Charadriiformes	SRR3218020
Loxia curvirostra	Red Crossbill	Fringillidae	Passeriformes	SRR834578
Lyrurus tetrix	Black Grouse	Phasianidae	Galliformes	SRR208078
Macronectes giganteus	Southern Giant Petrel	Procellariidae	Procellariiformes	SRR6902605
Malurus lamberti	Variegated Fairywren	Maluridae	Passeriformes	SRR3901709
Meleagris gallopavo	Wild Turkey	Phasianidae	Galliformes	SRR1796090
Melopsittacus undulatus	Budgerigar	Psittacidae	Psittaciformes	SRR5336547
Melospiza melodia	Song Sparrow	Passerellidae	Passeriformes	SRR1198303
Nesoptilotis leucotis	White-eared Honeyeater	Meliphagidae	Passeriformes	SRR3901721
Nothoprocta perdicaria	Chilean Tinamou	Tinamidae	Struthioniformes	SRR6713504
Oenanthe oenanthe	Northern Wheatear	Muscicapidae	Passeriformes	SRR6054179
Otus bakkamoena	Collared Scops Owl	Strigidae	Strigiformes	SRR3203243
Otus scops	Eurasian Scops Owl	Strigidae	Strigiformes	SRR3203230
Pandion haliaetus	Osprey	Pandionidae	Accipitriformes	SRR3218042
Patagioenas fasciata	Band-tailed Pigeon	Columbidae	Columbiformes	SRR3146192
Phasianus colchicus	Common Pheasant	Phasianidae	Galliformes	SRR1797835
Phylloscopus trochilus	Northern Willow Warbler	Phylloscopidae	Passeriformes	SRR528728
Phylloscopus trochilus	Willow Warbler	Phylloscopidae	Passeriformes	SRR529891
Picus canus	Grey-headed Woodpecker	Picidae	Piciformes	SRR3203240
Pipra filicauda	Wire-tailed Manakin	Pipridae	Passeriformes	SRR6811836
Platycercus eximius	Eastern Rosella	Psittacidae	Psittaciformes	SRR3901724

Poecile atricapillus	Black-capped Chickadee	Paridae	Passeriformes					SRR6255696
Pseudopodoces humilis	Ground Tit	Paridae	Passeriformes					SRR768235
Pygoscelis adeliae	Adelie Penguin	Spheniscidae	Sphenisciformes					SRR5253662
Pygoscelis papua	Gentoo Penguin	Spheniscidae	Sphenisciformes					SRR2980760
Sericornis frontalis	White-browed Scrubwren	Acanthizidae	Passeriformes					SRR3901710
Sinosuthora webbiana	Vinous-throated Parrotbill	Sylviidae	Passeriformes					SRR392516
Spinus spinus	Eurasian Siskin	Fringillidae	Passeriformes					SRR1551785
Spinus tristis	American Goldfinch	Fringillidae	Passeriformes					SRR6255718
Sturnus vulgaris	Common Starling	Sturnidae	Passeriformes					SRR3990508
Syrmaticus mikado	Mikado Pheasant	Phasianidae	Galliformes					SRR5666071
Tyto longimembris	Eastern Grass Owl	Tytonidae	Strigiformes					SRR3203222
Uraeginthus granatina	Violet-eared Waxbill	Estrildidae	Passeriformes					SRR955502
Zonotrichia albicollis	White-throated Sparrow	Passerellidae	Passeriformes					SRR3115424

C. Sequenced Genomes (MPIO)

Erythrocercus livingstonei	Livingstone's Flycatcher	Erythrocercidae	Passeriformes	UWBM	106107	Malawi	2001	SRR10852870
Eulacestoma nigropectus	Wattled Ploughbill	Eulacestomidae	Passeriformes	MVM	Z42739	P New Guinea		SRR10852869
Hyliota australis	Southern Hyliota	Hyliotidae	Passeriformes	UWBM	106011	Malawi	2001	SRR10852867
Ibidorhyncha struthersii	Ibisbill	Ibidorhynchidae	Charadriiformes	LMSU	R-138111 ID	Kazakhstan	1957	SRR10852866
Pluvianellus socialis	Magellanic Plover	Pluvianellidae	Charadriiformes	MACN	Ot-ct 210	Argentina		SRR10852865
Ptilorrhoa leucosticta	Spotted Jewel-Babbler	Cinclosomatidae	Passeriformes	ANWC	B52548	P New Guin	2009	SRR10852864
Pnoepyga pusilla	Pygmy Wren-Babbler	Pnoepygidae	Passeriformes	CGN		Indonesia		SRR10852863

D. Previously Published Genomes

Acanthisitta chloris	Rifleman	Acanthisittidae	Passeriformes					ASM69581v1
Alligator mississippiensis	American alligator	Alligatoridae	Crocodilians					ASM28112v4
Alligator sinensis	Chinese alligator	Alligatoridae	Crocodilians					ASM45574v1
Anas platyrhynchos	Mallard	Anatidae	Anseriformes					BGI_duck_1.0
Antrostomus carolinensis	Chuck-will's-widow	Caprimulgidae	Caprimulgiformes					ASM70074v1
Apaloderma vittatum	Bar-tailed Trogon	Trogonidae	Trogoniformes					ASM70340v1
Aptenodytes forsteri	Emperor Penguin	Spheniscidae	Sphenisciformes					ASM69914v1
Aquila chrysaetos	Golden Eagle	Accipitridae	Accipitriformes					Aquila_chrysaetos-1.0.2
Ara macao	Scarlet Macaw	Psittacidae	Psittaciformes					SMACv1.1
Athene cunicularia	Burrowing Owl	Strigidae	Strigiformes					athCun1
Balearica regulorum	Grey Crowned Crane	Gruidae	Gruiformes					ASM70989v1
Buceros rhinoceros	Rhinoceros Hornbill	Bucerotidae	Bucerotiformes					ASM71030v1
Calypte anna	Anna's Hummingbird	Trochilidae	Caprimulgiformes					ASM69908v1
Cariama cristata	Red-legged Seriema	Cariamidae	Cariamiformes					ASM69053v1
Cathartes aura	Turkey Vulture	Cathartidae	Cathartiformes					ASM69994v1
Chaetura pelagica	Chimney Swift	Apodidae	Caprimulgiformes					ChaPel_1.0
Charadrius vociferus	Killdeer	Charadriidae	Charadriiformes					ASM70802v2
Chlamydotis macqueenii	Macqueen's Bustard	Otididae	Otidiformes					ASM69519v1
Colius striatus	Speckled Mousebird	Coliidae	Coliiformes					ASM69071v1
Columba livia	Common Pigeon	Columbidae	Columbiformes					Cliv_1.0
Corvus brachyrhynchos	American Crow	Corvidae	Passeriformes					ASM69197v1
Corvus cornix	Hooded Crow	Corvidae	Passeriformes					Hooded_Crow
Cuculus canorus	Common Cuckoo	Cuculidae	Cuculiformes					ASM70932v1
Cyanistes caeruleus	Blue Tit	Paridae	Passeriformes					cyaCae2
Egretta garzetta	Little Egret	Ardeidae	Pelecaniformes					ASM68718v1
Eurypyga helias	Sunbittern	Eurypygidae	Eurypygiformes					ASM69077v1
Falco cherrug	Saker Falcon	Falconidae	Falconiformes					F_cherrug_v1.0
Falco peregrinus	Peregrine Falcon	Falconidae	Falconiformes					F_peregrinus_v1.0
Ficedula albicollis	Collared Flycatcher	Muscicapidae	Passeriformes					FicAlb1.5
Fulmarus glacialis	Northern Fulmar	Procellariidae	Procellariiformes					ASM69083v1
Gallus gallus	Red Junglefowl	Phasianidae	Galliformes					Gallus_gallus-4.0
Gavia_stellata genome	Red-throated Loon	Gaviidae	Gaviiformes					ASM69087v1
Geospiza fortis	Medium Ground Finch	Thraupidae	Passeriformes					GeoFor_1.0
Haliaeetus albicilla	White-tailed Eagle	Accipitridae	Accipitriformes					ASM69140v1
Haliaeetus leucocephalus	Bald Eagle	Accipitridae	Accipitriformes					Haliaeetus_leucocephalus-4.0
Leptosomus discolor	Cuckoo Roller	Leptosomidae	Leptosomiformes					ASM69178v1
Meleagris gallopavo	Wild Turkey	Phasianidae	Galliformes					Turkey_2.01
Melopsittacus undulatus	Budgerigar	Psittacidae	Psittaciformes					Melopsittacus_undulatus_6.3
Merops nubicus	Southern Carmine Bee-eater	Meropidae	Coraciiformes					ASM69184v1
Mesitornis unicolor	Brown Mesite	Mesitornithidae	Mesitornithiformes					ASM69576v1
Nestor notabilis	Kea	Strigopidae	Psittaciformes					ASM69687v1
Nipponia nippon	Crested Ibis	Threskiornithidae	Pelecaniformes					ASM70822v1
Opisthocomus hoazin	Hoatzin	Opisthocomidae	Opisthocomiformes					ASM69207v1
Pelecanus crispus	Dalmatian Pelican	Pelecanidae	Pelecaniformes					ASM68737v1
Phaethon lepturus	White-tailed Tropicbird	Phaethontidae	Phaethontiformes					ASM68728v1
Phalacrocorax carbo	Great Cormorant	Phalacrocoracidae	Suliformes					ASM70892v1
Phoenicopterus ruber	American Flamingo	Phoenicopteridae	Phoenicopteriformes					ASM68726v1
Picoides pubescens	Downy Woodpecker	Picidae	Piciformes					ASM69900v1
Podiceps cristatus	Great Crested Grebe	Podicipedidae	Podicipediformes					ASM69954v1
Pseudopodoces humilis	Ground Tit	Paridae	Passeriformes					PseHum1.0
Pterocles gutturalis	Yellow-throated Sandgrouse	Pteroclididae	Pterocliformes					ASM69924v1
Pygoscelis adeliae	Adelie Penguin	Spheniscidae	Sphenisciformes					ASM69910v1

Serinus canaria	Atlantic Canary	Fringillidae	Passeriformes	SCA1
Struthio camelus	Common Ostrich	Struthionidae	Struthioniformes	ASM69896v1
Taeniopygia guttata	Zebra Finch	Estrildidae	Passeriformes	Taeniopygia_guttata-3.2.4
Tauraco erythrolophus	Red-crested Turaco	Musophagidae	Musophagiformes	ASM70936v1
Tinamus guttatus	White-throated Tinamou	Tinamidae	Struthioniformes	ASM70537v2
Tyto alba	Barn Owl	Tytonidae	Strigiformes	ASM68720v1
Zonotrichia albicollis	White-throated Sparrow	Passerellidae	Passeriformes	Zonotrichia_albicollis-1.0.1

Institutes

AI	Alpenzoo Innsbruck, Austria
ANWC	Australian National Wildlife Collection, CSIRO, Canberra, Australia
CGN	Collection Gerhard Nikolaus, Germany
CKC	Collection Kai Clausen, Germany
CLB	Collection Ludger Bremehr, Verl, Germany
JGM	Johannes Gutenberg-Universität Mainz, Germany
LMO	Landesmuseum Oldenburg, Germany
LMSU	Lomonosov Moscow State University, Russia
LSUMNS	Lousiana State University Museum of Natural Science, Baton Rouge, USA
MACN	Museo Argentino De Ciencias Naturales, Argentina
MfN	Museum für Naturkunde, Leibniz-Institut für Evolutions- und Biodiversitätsforschung, Germany
MNHNC	Museo Nacional de Historia Natural, Chile
MPIO	Max Planck Institute for Ornithology, Seewiesen, Germany
MVM	Museum Victoria Melbourne, Australia
NARC	National Avian Research Center, Seihan, United Arab Emirates
PINP	Phillip Island Nature Parks, Cowes, Australia
THH	Tierpark Hagenbeck Hamburg, Germany
THM	Tierpark Hellabrunn München, Germany
TPB	Tierpark Berlin-Friedrichsfelde, Germany
TUM	Technical University of Munich, Germany
UFPB	Federal University of Para, Brazil
UG	University of Giessen, Germany
UGP	University of Gdansk, Poland
ULR	Université de La Réunion, Sainte Clotilde,France
UPS	Université Paris-Sud, Orsay, France
UV	University of Vienna, Austria
UW	Wageningen University and Research, The Netherlands
UWBM	University of Washington Burke Museum, Seattle, USA
VPW	Vogelpark Walsrode, Germany
WSU	Washington State University, Pullman, USA
WZS	Wilhelma Zoologisch Botanischer Garten Stuttgart, Germany
ZGB	Zoologischer Garten Berlin, Germany
ZGK	Zoologischer Garten Köln, Germany
ZGW	Zoologischer Garten Wuppertal, Germany
ZH	Zoo Heidelberg, Germany
ZZ	Zoo Zürich, Switzerland

Tab. S2A Sequenced Transcriptomes (MPIO)

Species name	Tissue	BASIC STATISTICS			BUSCO ANALYSIS RESULTS						MULTIPLE ALIGNMENT ANALYSIS			
		assembled transcripts	total length [bp]	transcript N50 [bp]	complete genes	complete (single copy)	complete (two copies)	fragmented genes	body genes	searched aves_odb9 genes	ref. 3'UTR covered	relative to best	ref. CDS covered	relative to best
Abroscopus superciliaris	body	31037	44521711	2303	55,4%	41,5%	13,9%	7,5%	37,1%	4915	8507246	27,1%	9432429	36,0%
Acanthiza pusilla	body	39277	65827129	2662	75,2%	48,2%	27,0%	5,4%	19,4%	4915	10643631	33,9%	13287852	50,7%
Accipiter nisus	brain	55253	101262938	3405	67,8%	43,6%	24,2%	6,7%	25,5%	4915	10966774	34,9%	12079653	46,1%
Acrocephalus palustris	body	39361	69317481	3107	66,3%	40,7%	25,6%	5,3%	28,4%	4915	10480166	33,4%	11544468	44,0%
Actophilornis africanus	blood	33625	50737283	2510	47,1%	29,7%	17,4%	7,5%	45,4%	4915	5980049	19,0%	7336662	28,0%
Aegithalos caudatus	blood	37787	60142225	2881	37,3%	23,6%	13,7%	8,0%	54,7%	4915	6231908	19,8%	7149273	27,3%
Aegithina tiphia	body	29166	45174068	2547	59,8%	44,0%	15,8%	7,0%	33,2%	4915	8746135	27,8%	10082232	38,4%
Aegotheles cristatus	muscle	4305	3145019	819	7,3%	5,8%	1,5%	3,1%	89,6%	4915	597305	1,9%	835170	3,2%
Agapornis fischeri	brain	83986	150781530	3519	74,8%	43,1%	31,7%	7,7%	17,5%	4915	13440472	42,8%	14668965	55,9%
Agapornis personatus	blood	47758	77791234	2995	44,8%	27,1%	17,7%	8,9%	46,3%	4915	6312162	20,1%	8262621	31,5%
Ailuroedus buccoides	blood	41373	71667835	3207	44,8%	25,2%	19,6%	7,9%	47,3%	4915	7157376	22,8%	8309598	31,7%
Alauda arvensis	brain	71569	134247470	3373	74,3%	31,4%	42,9%	7,2%	18,5%	4915	15397582	49,0%	14176248	54,1%
Alcippe morrisonia	body	54284	81342755	2577	69,2%	36,9%	32,3%	5,2%	25,6%	4915	11556000	36,8%	12492825	47,6%
Alectura lathami	blood	47953	80535460	2996	50,1%	30,5%	19,6%	8,5%	41,4%	4915	5928107	18,9%	8786835	33,5%
Alle alle	body	58978	41638895	902	31,3%	22,2%	9,1%	12,3%	56,4%	4915	6707988	21,4%	4212084	16,1%
Amadina erythrocephala	blood	31650	49222066	2697	37,6%	22,3%	15,3%	7,2%	55,2%	4915	6238732	19,9%	6567915	25,0%
Amadina fasciata	blood	32738	51393627	2764	42,4%	27,8%	14,6%	7,1%	50,5%	4915	6875261	21,9%	7419882	28,3%
Amandava amandava	muscle	76443	127440775	3042	69,3%	40,7%	28,6%	8,9%	21,8%	4915	15779553	50,2%	14418648	55,0%
Amazilia versicolor	body	68463	128528705	3463	75,7%	39,1%	36,6%	5,8%	18,5%	4915	11975845	38,1%	13984119	53,3%
Anastomus lamelligerus	body	39779	64718870	2802	63,9%	42,3%	21,6%	5,9%	30,2%	4915	8886519	28,3%	10952661	41,8%
Anhinga anhinga	body	8357	6339242	878	15,5%	14,0%	1,5%	6,5%	78,0%	4915	1456772	4,6%	1882404	7,2%
Anseranas semipalmata	blood	44852	78004432	3252	53,0%	31,5%	21,5%	6,7%	40,3%	4915	6707358	21,4%	8973006	34,2%
Anthracothorax nigricollis	liver	35870	59034737	2659	68,1%	43,0%	25,1%	6,0%	25,9%	4915	7816911	24,9%	11274180	43,0%
Aptenodytes patagonicus	brain	51216	92952477	3307	73,7%	48,2%	25,5%	6,4%	19,9%	4915	13085381	41,7%	13470939	51,4%
Apteryx mantelli mantelli	blood	61977	105576054	3224	56,3%	32,4%	23,9%	8,2%	35,5%	4915	6798191	21,6%	9830721	37,5%
Apus apus	brain	103773	199739717	3982	64,9%	34,8%	30,1%	14,3%	20,8%	4915	14484609	46,1%	15366885	58,6%
Aramus guarauna	body	33447	44101867	2054	50,8%	37,6%	13,2%	9,2%	40,0%	4915	7665860	24,4%	8126874	31,0%
Ardenna tenuirostris	body	39879	54955992	2165	53,4%	37,9%	15,5%	11,0%	35,6%	4915	9589220	30,5%	8464455	32,3%
Artamus cyanopterus	liver	34959	51257146	2365	57,5%	40,2%	17,3%	8,4%	34,1%	4915	9173737	29,2%	9873801	37,6%
Atelornis pittoides	liver	88347	108708544	2041	62,2%	36,5%	25,7%	11,5%	26,3%	4915	10240126	32,6%	12441093	47,4%
Attagis gayi	body	23289	28986966	1870	41,1%	32,0%	9,1%	9,2%	49,7%	4915	5581198	17,8%	6386454	24,4%
Balaeniceps rex	body	5287	3432823	712	6,3%	6,0%	0,3%	2,7%	91,0%	4915	1233805	3,9%	570276	2,2%
Bombycilla garrulus	blood	37956	63506638	3138	42,5%	26,0%	16,5%	7,6%	49,9%	4915	7072323	22,5%	7894497	30,1%
Bucco tamatia	body	33314	56573184	2875	61,1%	42,3%	18,8%	6,3%	32,6%	4915	7432169	23,7%	9870258	37,6%
Buceros bicornis	blood	53480	97698764	3527	51,6%	29,1%	22,5%	8,3%	40,1%	4915	7298993	23,2%	9408051	35,9%
Bugeranus carunculatus	blood	54208	94623818	3408	49,1%	28,3%	20,8%	8,3%	42,6%	4915	7877553	25,1%	8961222	34,2%
Buphagus erythrorhynchus	blood	23726	34367971	2412	34,4%	22,3%	12,1%	7,7%	57,9%	4915	5558385	17,7%	5479257	20,9%
Burhinus capensis	blood	50022	92108695	3519	54,5%	31,5%	23,0%	7,2%	38,3%	4915	8290674	26,4%	9481359	36,2%
Butorides striata	blood	35761	50466558	2315	49,0%	31,5%	17,5%	6,8%	44,2%	4915	6206673	19,8%	7932375	30,2%
Cacatua ducorpsii	blood	39211	68299750	3256	46,6%	29,6%	17,0%	7,7%	45,7%	4915	6491869	20,7%	8420988	32,1%
Calcarius lapponicus	body	40127	64060581	2715	63,8%	41,9%	21,9%	6,5%	29,7%	4915	10429285	33,2%	11286747	43,0%
Calliphlox amethystina	body	38915	66715066	2889	69,5%	45,5%	24,0%	6,7%	23,8%	4915	8688181	27,7%	11558103	44,1%
Campylopterus largipennis	liver	29142	46059143	2569	61,7%	43,2%	18,5%	6,1%	32,2%	4915	6666960	21,2%	9889857	37,7%
Caprimulgus inornatus	body	40322	56041363	2198	62,5%	40,6%	21,9%	6,9%	30,6%	4915	8021440	25,5%	10291068	39,2%
Cardinalis cardinalis	blood	45221	74400664	3052	47,1%	29,2%	17,9%	9,5%	43,4%	4915	8677375	27,6%	8980440	34,2%
Carduelis cannabina	blood	27650	42728062	2659	35,5%	23,4%	12,1%	8,1%	56,4%	4915	6329814	20,1%	6609036	25,2%
Cariama cristata	blood	44316	66702746	2566	56,0%	32,9%	23,1%	5,8%	38,2%	4915	7125570	22,7%	9199947	35,1%
Casuarius casuarius	body	5311	4372148	1021	11,0%	10,2%	0,8%	4,0%	85,0%	4915	835098	2,7%	1272189	4,9%
Cathartes melambrotus	muscle	35219	57342136	2797	62,6%	47,3%	15,3%	6,6%	30,8%	4915	9346041	29,8%	10939824	41,7%
Centropus superciliosus	body	68382	131810473	3710	74,7%	41,0%	33,7%	7,0%	18,3%	4915	11677773	37,2%	14098692	53,8%
Cereopsis novaehollandiae	blood	48478	84285559	3257	51,5%	30,7%	20,8%	8,1%	40,4%	4915	6384877	20,3%	8911347	34,0%
Certhia brachydactyla	blood	39775	68250186	3256	38,8%	25,0%	13,8%	7,3%	53,9%	4915	6702278	21,3%	7744467	29,5%
Cettia diphone	muscle	36735	51268300	2240	59,2%	43,1%	16,1%	7,5%	33,3%	4915	9194696	29,3%	10270689	39,2%
Chaetops aurantius	muscle	38863	49990063	2002	57,7%	42,3%	15,4%	8,4%	33,9%	4915	9224899	29,4%	9873594	37,6%
Charadrius hiaticula	blood	45735	80799594	3337	50,6%	30,4%	20,2%	6,9%	42,5%	4915	7364063	23,4%	8577651	32,7%
Chauna torquata	blood	48775	83123913	3141	53,9%	31,1%	22,8%	6,9%	39,2%	4915	6771370	21,6%	9216603	35,1%
Chionis albus	body	33886	47977420	1955	6,4%	2,7%	3,7%	1,2%	92,4%	4915	8796844	28,0%	9605586	36,6%
Chloebia gouldiae	muscle	74048	113624574	2846	74,7%	35,0%	39,7%	4,3%	21,0%	4915	12608579	40,1%	14013096	53,4%
Chloris chloris	blood	26941	42291889	2734	32,5%	21,0%	11,5%	7,1%	60,4%	4915	5804467	18,5%	6182283	23,6%
Chloropsis cochinchinensis	blood	36303	59696080	3027	40,3%	24,6%	15,7%	7,5%	52,2%	4915	6393270	20,4%	7295781	27,8%
Chlorostilbon notatus	brain	58593	114638100	3719	72,8%	43,8%	29,0%	6,6%	20,6%	4915	11781216	37,5%	13443360	51,3%
Chroicocephalus ridibundus	brain	64063	114492650	3386	80,2%	47,9%	32,3%	5,4%	14,4%	4915	13607467	43,3%	14853327	56,6%
Cinclosoma clarum	body	32789	41856544	1887	42,1%	28,3%	13,8%	10,6%	47,3%	4915	7950696	25,3%	6682572	25,5%
Cinclus cinclus	blood	31749	50177375	2790	39,8%	26,6%	13,2%	7,8%	52,4%	4915	5903569	18,8%	7105398	27,1%
Cisticola galactotes	blood	38881	64661075	3103	40,2%	25,4%	14,8%	8,7%	51,1%	4915	7119059	22,7%	7623792	29,1%
Climacteris picumnus	body	29909	40878145	2125	46,8%	34,2%	12,6%	10,1%	43,1%	4915	8150665	25,9%	7455261	28,4%
Clytospiza monteiri	blood	58545	85167647	2739	46,7%	23,0%	23,7%	5,8%	47,5%	4915	8350542	26,6%	8701476	33,2%
Cnemophilus loriae	body	35613	53123374	2464	59,0%	41,3%	17,7%	7,0%	34,0%	4915	9763881	31,1%	9931851	37,9%
Coccothraustes coccothraust	blood	30019	41883238	2313	35,7%	22,4%	13,3%	6,7%	57,6%	4915	5406495	17,2%	6329553	24,1%
Coereba flaveola	body	28666	30912277	1554	44,0%	36,9%	7,1%	10,5%	45,5%	4915	8100566	25,8%	7634169	29,1%
Colinus virginianus	blood	36992	62229864	2964	49,8%	28,0%	21,8%	7,0%	43,2%	4915	4170604	13,3%	8277120	31,6%
Columba palumbus	brain	65671	125885116	3749	77,1%	46,3%	30,8%	6,1%	16,8%	4915	13128956	41,8%	14525805	55,4%
Conopophaga lineata	body	39429	75599131	3374	73,8%	47,0%	26,8%	3,9%	22,3%	4915	10918061	34,8%	12839487	49,0%
Coracias caudatus	blood	45383	76446317	3111	47,4%	27,9%	19,5%	7,9%	44,7%	4915	6395061	20,4%	8269140	31,5%
Coracina novaehollandiae	body	18505	15789808	1023	16,3%	13,5%	2,8%	8,2%	75,5%	4915	5401378	17,2%	2321505	8,9%
Corcorax melanorhamphos	liver	21211	20029708	1159	21,3%	18,8%	2,5%	9,8%	68,9%	4915	6999944	22,3%	2877615	11,0%
Corvus monedula	body	51890	95045994	3536	71,0%	48,7%	22,3%	7,0%	22,0%	4915	14492858	46,1%	13389105	51,1%
Cracticus tibicen	muscle	38668	56710246	2338	57,9%	40,2%	17,7%	10,1%	32,0%	4915	10599672	33,7%	9710520	37,0%
Crotophaga ani	brain	61976	121384115	3704	76,1%	40,5%	35,6%	6,8%	17,1%	4915	12908669	41,1%	14069355	53,6%
Crypturellus tataupa	blood	43972	71643802	2852	56,4%	37,8%	18,6%	7,6%	36,0%	4915	4800380	15,3%	9182121	35,0%
Cyanoramphus novaezelandi	blood	44252	73593767	3102	44,8%	26,1%	18,7%	8,3%	46,9%	4915	6303035	20,1%	7995447	30,5%
Dacelo novaeguineae	blood	37390	61715105	3026	47,9%	32,2%	15,7%	7,4%	44,7%	4915	6456007	20,6%	8173629	31,2%
Daphoenositta chrysoptera	muscle	33707	40953044	1806	48,7%	32,7%	16,0%	9,5%	41,8%	4915	7705279	24,5%	8021565	30,6%
Dendrocopos major	body	50787	87780388	3178	70,6%	50,0%	20,6%	6,8%	22,6%	4915	10132686	32,3%	12861012	49,0%
Dicaeum geelvinkianum	muscle	7308	6228016	1070	16,5%	13,5%	3,0%	5,4%	78,1%	4915	1857066	5,9%	1571235	6,0%
Dicrurus adsimilis	blood	35327	58467363	3069	45,6%	28,8%	16,8%	8,0%	46,4%	4915	7514776	23,9%	7965765	30,4%
Dicrurus paradiseus	body	50349	72169135	2455	67,7%	42,4%	25,3%	5,6%	26,7%	4915	11054289	35,2%	12252747	46,7%
Donacobius atricapillus	body	35359	46985840	2062	55,9%	40,4%	15,5%	8,5%	35,6%	4915	9275277	29,5%	9307329	35,5%
Dromaius novaehollandiae	body	30761	39713600	1923	45,5%	35,3%	10,2%	11,6%	42,9%	4915	6538443	20,8%	6874293	26,2%
Dromas ardeola	body	27920	44230724	2639	60,4%	48,4%	12,0%	6,4%	33,2%	4915	8029173	25,6%	10221261	39,0%
Elminia albonotata	body	37211	59339586	2680	65,4%	47,2%	18,2%	5,7%	28,9%	4915	9727362	31,0%	11259879	42,9%
Emberiza citrinella	blood	56417	78634337	2536	45,3%	21,9%	23,4%	6,4%	48,3%	4915	7651159	24,4%	8297811	31,6%
Emblema pictum	brain	40460	60467886	2421	65,0%	44,5%	20,5%	6,9%	28,1%	4915	10269935	32,7%	11482329	43,8%
Erythrura psittacea	blood	36090	55068602	2725	39,6%	26,0%	13,6%	8,2%	52,2%	4915	6405531	20,4%	7429332	28,3%
Estrilda melpoda	brain	39640	61516787	2572	63,7%	41,3%	22,4%	6,8%	29,5%	4915	10023641	31,9%	11198100	42,7%
Eubucco versicolor	body	14880	13021946	1052	16,8%	14,2%	2,6%	7,0%	76,2%	4915	2762155	8,8%	2207463	8,4%
Eudromia elegans	blood	48941	82826474	3043	52,2%	28,8%	23,4%	8,2%	39,6%	4915	4423264	14,1%	8835726	33,7%
Euodice cantans	blood	78118	109908286	2597	48,3%	20,2%	28,1%	7,7%	44,0%	4915	9218146	29,3%	9435567	36,0%
Eurylaimus ochromalus	body	33135	55593429	2900	61,1%	39,3%	21,8%	6,2%	32,7%	4915	8982158	28,6%	10253439	39,1%
Eurypyga helias	body	44747	72453352	2756	69,7%	42,9%	26,8%	5,0%	25,3%	4915	8090132	25,8%	11825856	45,1%
Eurystomus glaucurus	body	33110	44995997	2120	55,2%	39,0%	16,2%	7,9%	36,9%	4915	7593839	24,2%	8674224	33,1%
Euschistospiza dybowskii	body	16831	16229471	1227	27,4%	22,3%	5,1%	8,7%	63,9%	4915	4775884	15,2%	3779490	14,4%
Falco sparverius	blood	25908	38045548	2308	43,2%	32,1%	11,1%	7,3%	49,5%	4915	6234443	19,8%	6352263	24,2%
Falculea palliata	blood	33879	52179723	2687	40,5%	26,6%	13,9%	8,4%	51,1%	4915	6829276	21,7%	7103451	27,1%

Florisuga fuscus	body	77841	143066846	3376	77,1%	36,2%	40,9%	5,2%	17,7%	4915	12223136	38,9%	14181294	54,1%
Florisuga mellivora	body	102028	185694003	3219	79,9%	28,3%	51,6%	6,2%	13,9%	4915	13314308	42,4%	14956500	57,0%
Fregata magnificens	body	31063	36534949	1670	33,8%	27,1%	6,7%	12,2%	54,0%	4915	7300678	23,2%	6031908	23,0%
Fringilla coelebs	brain	53500	102325060	3500	75,4%	40,3%	35,1%	5,1%	19,5%	4915	15301023	48,7%	14035704	53,5%
Furnarius rufus	skin	50274	91731254	3231	73,0%	45,5%	27,5%	6,2%	20,8%	4915	12332071	39,3%	13208391	50,4%
Galbula ruficauda	body	30541	51246445	2812	58,0%	39,4%	18,6%	6,2%	35,8%	4915	6971547	22,2%	9361479	35,7%
Garrulax leucolophus	blood	37786	58002294	2783	44,9%	29,8%	15,1%	8,2%	46,9%	4915	7429960	23,7%	8160708	31,1%
Gavia stellata	body	38523	63869093	2896	65,6%	47,3%	18,3%	6,8%	27,6%	4915	10220249	32,5%	11343321	43,3%
Geokichla dohertyi	blood	33641	54710831	3044	42,6%	29,5%	13,1%	8,0%	49,4%	4915	6614720	21,1%	7930215	30,2%
Geronticus eremita	blood	38774	65719245	3014	56,9%	36,9%	20,0%	6,1%	37,0%	4915	7526534	24,0%	9305184	35,5%
Gerygone flavolateralis	skin	45557	76449686	2777	75,7%	50,4%	25,3%	6,4%	17,9%	4915	12854792	40,9%	13638423	52,0%
Glaucidium nanum	liver	34021	35132531	1390	36,0%	28,9%	7,1%	13,4%	50,6%	4915	7503256	23,9%	5866950	22,4%
Grallina cyanoleuca	body	31467	35810290	1603	34,1%	24,9%	9,2%	12,6%	53,3%	4915	8492802	27,0%	5734482	21,9%
Gymnoderus foetidus	blood	29182	41230702	2222	39,3%	26,5%	12,8%	8,7%	52,0%	4915	6726659	21,4%	5999169	22,9%
Haematopus palliatus	blood	41182	67293542	2864	51,5%	32,5%	19,0%	6,8%	41,7%	4915	7211958	23,0%	8575902	32,7%
Heliornis fulica	body	8666	6991791	972	18,2%	15,6%	2,6%	5,9%	75,9%	4915	1528740	4,9%	1901847	7,3%
Hemiprocne mystacea	body	20028	22348689	1626	23,9%	18,2%	5,7%	9,3%	66,8%	4915	3203657	10,2%	4672146	17,8%
Heteromunia pectoralis	body	24330	25670228	1491	33,3%	24,7%	8,6%	10,5%	56,2%	4915	6941013	22,1%	5089041	19,4%
Himantopus himantopus	blood	42293	74848889	3329	49,4%	27,8%	21,6%	6,0%	44,6%	4915	6779365	21,6%	8349249	31,8%
Hirundo rustica	brain	28845	40350690	2316	51,3%	37,4%	13,9%	9,2%	39,5%	4915	7683254	24,5%	9370950	35,7%
Hylia prasina	muscle	37437	59129214	2601	62,3%	38,0%	24,3%	6,9%	30,8%	4915	10139072	32,3%	10164723	38,8%
Hypargos niveoguttatus	blood	64000	97116457	2845	58,9%	31,9%	27,0%	5,6%	35,5%	4915	10137005	32,3%	10667436	40,7%
Hypocolius ampelinus	blood	29997	44040781	2468	38,2%	26,6%	11,6%	7,6%	54,2%	4915	6975636	22,2%	6276780	23,9%
Ifrita kowaldi	body	33481	51020628	2554	57,0%	41,9%	15,1%	7,2%	35,8%	4915	9291806	29,6%	9667266	36,9%
Indicator indicator	body	25843	30279439	1784	36,1%	28,7%	7,4%	9,7%	54,2%	4915	4751061	15,1%	6334527	24,2%
Irena puella	blood	23616	35744981	2540	39,8%	23,9%	15,9%	6,5%	53,7%	4915	5631710	17,9%	5877795	22,4%
Ispidina picta	body	28336	33321090	1716	46,6%	33,7%	12,9%	8,3%	45,1%	4915	5451085	17,4%	7203312	27,5%
Lagonosticta rubricata	blood	39328	67555761	3220	47,2%	29,0%	18,2%	8,3%	44,5%	4915	7866688	25,0%	8646600	33,0%
Lagopus lagopus	blood	37156	61084508	2952	48,9%	29,9%	19,0%	6,3%	44,8%	4915	4545367	14,5%	7918665	30,2%
Lamprospiza melanoleuca	body	37630	50136236	2056	57,3%	38,5%	18,8%	8,6%	34,1%	4915	9519081	30,3%	9904575	37,8%
Laniarius funebris	blood	40190	67926963	3161	45,3%	27,4%	17,9%	7,9%	46,8%	4915	7439585	23,7%	8180205	31,2%
Lanius collurio	brain	67068	129981340	3614	68,5%	35,2%	33,3%	9,7%	21,8%	4915	15882385	50,6%	13596015	51,8%
Larosterna inca	liver	33002	42259123	1975	54,6%	44,0%	10,6%	9,2%	36,2%	4915	7246995	23,1%	9332040	35,6%
Larus crassirostris	blood	43954	69015446	2903	47,6%	31,7%	15,9%	7,2%	45,2%	4915	6492071	20,7%	7970394	30,4%
Laterallus leucopyrrhus	blood	49045	80150915	3007	52,0%	30,8%	21,2%	7,3%	40,7%	4915	6652375	21,2%	9101853	34,7%
Leiothrix lutea	blood	35694	54701888	2658	44,0%	26,4%	17,6%	7,4%	48,6%	4915	6380723	20,3%	7840545	29,9%
Leptosomus discolor	blood	34749	54059225	2575	42,8%	28,2%	14,6%	10,0%	47,2%	4915	6576806	20,9%	7127229	27,2%
Leucopsar rothschildi	blood	45198	75908175	3191	51,8%	34,8%	17,0%	9,0%	39,2%	4915	8930741	28,4%	9678276	36,9%
Lipaugus vociferans	body	96388	193043240	3818	78,8%	35,5%	43,3%	6,8%	14,4%	4915	17137716	54,6%	15530487	59,2%
Locustella naevia	body	13290	9679550	806	14,3%	11,5%	2,8%	5,4%	80,3%	4915	3024043	9,6%	1807305	6,9%
Lonchura striata	brain	52172	96594188	3447	72,5%	46,1%	26,4%	7,3%	20,2%	4915	15389218	49,0%	13536945	51,6%
Lophophanes cristatus	blood	35559	58901537	3048	35,4%	22,3%	13,1%	9,0%	55,6%	4915	6828502	21,7%	6896874	26,3%
Lophorina superba	body	25203	36776961	2408	51,2%	37,9%	13,3%	7,1%	41,7%	4915	8178507	26,0%	8087115	30,8%
Lophornis magnificus	brain	49293	88503907	3291	70,0%	45,5%	24,5%	7,6%	22,4%	4915	10596023	33,7%	12776058	48,7%
Malacoptila panamensis	body	34055	51424322	2398	53,9%	36,2%	17,7%	8,9%	37,2%	4915	6988136	22,2%	8683893	33,1%
Malurus melanocephalus	brain	62882	109635799	2997	63,8%	33,0%	30,8%	10,8%	25,4%	4915	14654150	46,6%	12070638	46,0%
Manacus vitellinus	brain	53030	94398294	3178	73,3%	45,1%	28,2%	6,0%	20,7%	4915	13598105	43,3%	13415526	51,2%
Mandingoa nitidula	body	40140	52624385	2012	59,7%	40,5%	19,2%	9,1%	31,2%	4915	9765125	31,1%	10500222	40,0%
Megalaima asiatica	blood	40346	62623777	2741	44,3%	26,7%	17,6%	7,2%	48,5%	4915	4536849	14,4%	7670688	29,2%
Melanerpes cruentatus	brain	71071	122700646	3223	71,9%	42,5%	29,4%	9,8%	18,3%	4915	11009167	35,0%	14091873	53,7%
Melanocharis versteri	body	26532	38645763	2299	54,3%	36,5%	17,8%	7,8%	37,9%	4915	8130847	25,9%	8558295	32,6%
Melanodryas cucullata	body	1919	1274562	740	3,3%	3,0%	0,3%	1,6%	95,1%	4915	333379	1,1%	336030	1,3%
Menura novaehollandiae	body	32018	42445937	2037	48,3%	37,5%	10,8%	10,1%	41,6%	4915	8583562	27,3%	7824252	29,8%
Micrastur ruficollis	body	31744	40571666	1957	51,1%	39,6%	11,5%	9,2%	39,7%	4915	7199626	22,9%	8174502	31,2%
Microeca papuana	liver	54503	88820930	2914	72,0%	35,9%	36,1%	4,6%	23,4%	4915	11647847	37,1%	13033800	49,7%
Milvago chimachima	muscle	27906	42141515	2421	57,0%	42,9%	14,1%	6,6%	36,4%	4915	7468709	23,8%	8629179	32,9%
Mimus saturninus	skin	52656	92297360	3139	75,9%	49,0%	26,9%	6,6%	17,5%	4915	13584722	43,2%	13995096	53,4%
Molothrus ater	body	30626	48268825	2660	57,2%	39,9%	17,3%	7,3%	35,5%	4915	9642588	30,7%	9651918	36,8%
Momotus momota	body	32683	42026298	1909	49,8%	35,0%	14,8%	8,5%	41,7%	4915	6310239	20,1%	7622913	29,1%
Monarcha melanopsis	body	32777	46598616	2276	54,8%	41,9%	12,9%	9,2%	36,0%	4915	9556977	30,4%	9003867	34,3%
Morus bassanus	blood	51963	89808136	3262	52,7%	31,7%	21,0%	8,1%	39,2%	4915	8007139	25,5%	9465897	36,1%
Motacilla flava	blood	33887	54594189	2957	38,9%	23,7%	15,2%	6,8%	54,3%	4915	6274614	20,0%	6972744	26,6%
Musophaga violacea	blood	62685	106022226	3326	50,3%	30,9%	19,4%	10,2%	39,5%	4915	8406644	26,8%	9758961	37,2%
Myiozetetes cayanensis	brain	62309	100118320	2991	78,9%	62,8%	16,1%	5,6%	15,5%	4915	15662420	49,9%	12952533	49,4%
Nectarinia tacazze	blood	41958	69880007	3094	47,8%	30,8%	17,0%	8,1%	44,1%	4915	8030046	25,6%	8903043	33,9%
Neochmia temporalis	blood	28290	41438713	2450	36,8%	24,7%	12,1%	8,1%	55,1%	4915	6482124	20,6%	6586260	25,1%
Nestor meridionalis	blood	55248	89482348	3065	45,4%	27,5%	17,9%	9,1%	45,5%	4915	7099154	22,6%	8757834	33,4%
Nicator chloris	body	33310	46913544	2241	57,2%	39,3%	17,9%	7,4%	35,4%	4915	9012555	28,7%	9618543	36,7%
Nigrita canicapilla	body	22486	22787012	1388	27,9%	23,4%	4,5%	11,9%	60,2%	4915	6089799	19,4%	5243652	20,0%
Numida meleagris	body	56574	100849971	3248	75,9%	50,3%	25,6%	6,6%	17,5%	4915	8583748	27,3%	13454580	51,3%
Nyctibius grandis	body	20283	16736293	979	15,2%	12,1%	3,1%	8,3%	76,5%	4915	4407445	14,0%	2362767	9,0%
Nyctidromus albicollis	brain	56636	108661503	3442	76,0%	36,1%	39,9%	5,7%	18,3%	4915	12577603	40,0%	13607385	51,9%
Nymphicus hollandicus	brain	86794	160560573	3508	76,5%	38,8%	37,7%	7,0%	16,5%	4915	13943960	44,4%	14842110	56,6%
Oceanites oceanicus	muscle	54578	84972174	2617	71,0%	46,6%	24,4%	6,8%	22,2%	4915	10791566	34,4%	12810093	48,8%
Oceanodroma leucorhoa	body	4873	3292428	744	3,5%	3,1%	0,4%	1,7%	94,8%	4915	676882	2,2%	592179	2,3%
Opisthocomus hoazin	muscle	38886	53279268	2220	62,7%	47,1%	15,6%	7,0%	30,3%	4915	8458640	26,9%	10082196	38,4%
Oriolus oriolus	blood	39319	62660428	2947	41,3%	27,0%	14,3%	8,7%	50,0%	4915	6893427	21,9%	7750782	29,6%
Orthonyx temminckii	liver	26627	30883943	1641	38,5%	29,8%	8,7%	11,3%	50,2%	4915	7700046	24,5%	6304224	24,0%
Ortygospiza atricollis	blood	60478	86096566	2574	47,2%	19,7%	27,5%	6,2%	46,6%	4915	7916470	25,2%	8566542	32,7%
Otis tarda	blood	53365	88089908	3078	53,5%	31,3%	22,2%	7,5%	39,0%	4915	7768496	24,7%	9463059	36,1%
Pachycephala rufiventris	body	15229	11042788	803	13,3%	11,0%	2,3%	7,7%	79,0%	4915	3311619	10,5%	2036484	7,8%
Padda oryzivora	brain	35931	53539021	2422	60,9%	43,8%	17,1%	6,5%	32,6%	4915	9491903	30,2%	10521144	40,1%
Panurus biarmicus	blood	32809	51431114	2791	34,5%	22,0%	12,5%	8,1%	57,4%	4915	5723183	18,2%	6594036	25,1%
Parabuteo unicinctus	blood	25929	38134315	2312	43,2%	31,9%	11,3%	7,3%	49,5%	4915	6940679	22,1%	6832542	26,1%
Paramythia montium	liver	68675	105856651	2810	72,9%	36,0%	36,9%	4,1%	23,0%	4915	12467612	39,7%	13255056	50,5%
Pardalotus punctatus	muscle	35157	50493387	2308	55,4%	38,4%	17,0%	10,2%	34,4%	4915	10241038	32,6%	9714588	37,0%
Parmoptila jamesoni	body	40073	57059361	2284	58,3%	41,4%	16,9%	8,4%	33,3%	4915	9928998	31,6%	10062741	38,4%
Parus major	brain	59148	120566709	3944	74,9%	45,6%	29,3%	6,6%	18,5%	4915	15954260	50,8%	14444187	55,1%
Passer domesticus	body	99016	156758144	3089	79,7%	35,4%	44,3%	4,9%	15,4%	4915	16364418	52,1%	15701646	59,9%
Pauxi pauxi	blood	48897	87086271	3477	49,3%	29,7%	19,6%	8,8%	41,9%	4915	5793528	18,4%	8854389	33,8%
Pedionomus torquatus	muscle	3329	2182790	710	6,1%	5,6%	0,5%	2,6%	91,3%	4915	597525	1,9%	537414	2,0%
Pelecanus onocrotalus	blood	48500	78592471	2971	48,3%	28,6%	19,7%	8,7%	43,0%	4915	7565013	24,1%	8692446	33,1%
Pellorneum capistratum	body	40047	55130289	2120	56,6%	37,6%	19,0%	8,9%	34,5%	4915	9339938	29,7%	10594563	40,4%
Periparus ater	blood	36144	59682848	3045	42,0%	25,2%	16,8%	7,4%	50,6%	4915	7244400	23,1%	7611279	29,0%
Peucedramus taeniatus	body	2580	1775909	765	5,0%	4,5%	0,5%	2,0%	93,0%	4915	438378	1,4%	534210	2,0%
Phaethon lepturus	body	4192	2608926	630	1,7%	1,6%	0,1%	1,2%	97,1%	4915	416259	1,3%	236373	0,9%
Phaethornis ruber	liver	41750	73049441	3054	71,9%	45,3%	26,6%	4,7%	23,4%	4915	8684298	27,6%	12267156	46,8%
Phaethornis superciliosus	brain	69070	135521974	3735	72,4%	44,8%	27,6%	8,9%	18,7%	4915	12429264	39,6%	14180784	54,1%
Phainopepla nitens	body	5566	3285118	606	6,2%	5,4%	0,8%	3,0%	90,8%	4915	1188542	3,8%	652305	2,5%
Phalacrocorax carbo	brain	56396	99741133	3387	77,1%	51,0%	26,1%	5,6%	17,3%	4915	13092620	41,7%	14335695	54,7%
Pheugopedius genibarbis	brain	63997	124410496	3752	72,9%	44,4%	28,5%	8,2%	18,9%	4915	16205382	51,6%	14311614	54,6%
Philomachus pugnax	liver	30944	43873695	2204	63,3%	46,5%	16,8%	6,5%	30,2%	4915	7124169	22,7%	10024977	38,2%
Phodilus badius	blood	54583	95330419	3253	52,0%	29,3%	22,7%	8,4%	39,6%	4915	8082830	25,7%	9399459	35,8%
Phoebastria immutabilis	body	26648	30942905	1691	40,5%	33,3%	7,2%	9,9%	49,6%	4915	7109901	22,6%	6432060	24,5%
Phoenicopterus roseus	blood	42960	72233373	3035	52,0%	31,7%	20,3%	7,2%	40,8%	4915	7262744	23,1%	8618115	32,9%
Phoenicurus ochruros	brain	65599	127440695	3641	75,2%	41,0%	34,2%	7,9%	16,9%	4915	16034290	51,0%	14542857	55,5%
Phonygammus keraudrenii	muscle	26974	34667443	2004	44,9%	35,9%	9,0%	9,7%	45,4%	4915	8111059	25,8%	7768722	29,6%
Phylidonyris novaehollandiae	body	35021	53027287	2388	68,8%	51,4%	17,4%	5,8%	25,4%	4915	9383392	29,9%	12193407	46,5%
Phylloscopus umbrovirens	body	27310	33750500	1890	53,5%	42,9%	10,6%	7,7%	38,8%	4915	7469553	23,8%	8591220	32,8%
Picathartes gymnocephalus	body	6868	4202345	652	8,4%	7,9%	0,5%	4,2%	87,4%	4915	1489390	4,7%	859929	3,3%
Picus viridis	brain	46380	74778666	2857	64,0%	47,0%	17,0%	7,9%	28,1%	4915	7893384	25,1%	11431428	43,6%

Pitta versicolor	body	33056	45649110	2138	56,1%	41,7%	14,4%	8,7%	35,2%	4915	8248893	26,3%	8856756	33,8%
Platylophus galericulatus	body	37678	57195978	2497	57,3%	37,1%	20,2%	7,7%	35,0%	4915	9408365	29,9%	10100574	38,5%
Platysteira castanea	muscle	38777	64574271	2831	64,3%	42,1%	22,2%	7,0%	28,7%	4915	11466833	36,5%	11002086	41,9%
Plectropterus gambensis	blood	38702	64127422	2960	48,8%	31,0%	17,8%	6,8%	44,4%	4915	5275640	16,8%	8015592	30,6%
Plocepasser mahali	liver	37261	49400272	2037	65,6%	48,8%	16,8%	7,3%	27,1%	4915	9489543	30,2%	10731825	40,9%
Ploceus bicolor	brain	53857	100228065	3477	68,4%	44,5%	23,9%	7,7%	23,9%	4915	14590983	46,4%	13122414	50,0%
Pluvianus aegyptius	blood	49548	85492845	3157	51,6%	31,7%	19,9%	7,5%	40,9%	4915	7547838	24,0%	8899527	33,9%
Podargus strigoides	body	59152	99518866	3127	53,5%	27,1%	26,4%	8,1%	38,4%	4915	8081046	25,7%	9842016	37,5%
Podiceps nigricollis	blood	47660	77864091	2992	51,6%	29,8%	21,8%	7,7%	40,7%	4915	7252041	23,1%	9027483	34,4%
Poecile montanus	blood	30881	50436311	3004	37,4%	24,3%	13,1%	7,1%	55,5%	4915	5957864	19,0%	6917019	26,4%
Poecile palustris	liver	25866	40998810	2723	52,9%	42,0%	10,9%	7,2%	39,9%	4915	7517820	23,9%	9016173	34,4%
Poephila acuticauda	blood	34170	54314576	2818	45,4%	27,9%	17,5%	7,1%	47,5%	4915	6760828	21,5%	7753392	29,6%
Poephila personata	blood	67303	94318494	2580	49,2%	23,3%	25,9%	6,8%	44,0%	4915	9037509	28,8%	9165189	34,9%
Pogoniulus atroflavus	blood	43577	61442278	2378	40,1%	24,9%	15,2%	7,8%	52,1%	4915	4020997	12,8%	7458174	28,4%
Pogoniulus bilineatus	body	12683	11158607	1113	20,4%	17,3%	3,1%	6,2%	73,4%	4915	2103550	6,7%	2920386	11,1%
Polioptila albiloris	body	28417	36787813	1994	46,4%	36,5%	9,9%	10,2%	43,4%	4915	8353442	26,6%	7824360	29,8%
Pomatostomus superciliosus	body	29072	35576758	1821	42,3%	32,6%	9,7%	11,2%	46,5%	4915	7692990	24,5%	6990555	26,7%
Prionops plumatus	body	32378	48114434	2517	59,0%	44,0%	15,0%	6,2%	34,8%	4915	9036212	28,8%	10026036	38,2%
Promerops cafer	blood	37780	62919202	3099	38,6%	24,2%	14,4%	6,8%	54,6%	4915	6620249	21,1%	7059873	26,9%
Prunella modularis	blood	33834	54281313	2890	38,4%	23,9%	14,5%	6,9%	54,7%	4915	6452786	20,5%	7063710	26,9%
Psilopogon pyrolophus	blood	44766	69943518	2837	43,6%	27,2%	16,4%	9,0%	47,4%	4915	4996531	15,9%	7874352	30,0%
Psittacus erithacus	blood	47169	77524005	3097	46,2%	28,4%	17,8%	8,4%	45,4%	4915	6668531	21,2%	8616348	32,9%
Psophia crepitans	blood	54981	92285658	3191	46,2%	26,4%	19,8%	9,8%	44,0%	4915	7094565	22,6%	8843091	33,7%
Psophodes olivaceus	muscle	25294	27069984	1488	35,3%	26,7%	8,6%	10,8%	53,9%	4915	7270861	23,1%	5740401	21,9%
Pterocles orientalis	blood	47553	79543218	3097	48,3%	25,7%	22,6%	7,7%	44,0%	4915	6754291	21,5%	8491524	32,4%
Pterodroma baraui	body	24905	24015306	1294	21,9%	16,9%	5,0%	10,9%	67,2%	4915	5110777	16,3%	3293253	12,6%
Pteroptochos megapodius	liver	37026	54454014	2458	57,1%	42,3%	14,8%	6,4%	36,5%	4915	8821060	28,1%	9223830	35,2%
Pteruthius flaviscapis	liver	77106	113102771	2707	74,0%	37,6%	36,4%	4,2%	21,8%	4915	12905675	41,1%	13540713	51,6%
Ptiloris magnificus	muscle	31390	43512852	2156	52,2%	40,4%	11,8%	11,1%	36,7%	4915	10120540	32,2%	8249667	31,5%
Pycnonotus xanthopygos	blood	35461	60579990	3287	39,5%	24,7%	14,8%	7,8%	52,7%	4915	6741357	21,5%	7651557	29,2%
Pyrrhula pyrrhula	brain	75766	123991335	2896	76,6%	46,9%	29,7%	7,2%	16,2%	4915	16597502	52,8%	15295365	58,3%
Pytilia phoenicoptera	blood	37003	61804692	3038	46,8%	27,5%	19,3%	7,2%	46,0%	4915	7337354	23,4%	8263299	31,5%
Ramphastos toco	blood	49947	75073032	2702	45,0%	28,7%	16,3%	8,6%	46,4%	4915	5351631	17,0%	8231487	31,4%
Ramphocelus carbo	brain	59349	114048723	3522	74,0%	39,8%	34,2%	6,2%	19,8%	4915	15583273	49,6%	13944702	53,2%
Regulus regulus	blood	55361	99446549	3370	73,4%	48,2%	25,2%	7,3%	19,3%	4915	15078619	48,0%	13767138	52,5%
Remiz pendulinus	blood	43199	74622086	3231	46,1%	29,3%	16,8%	8,8%	45,1%	4915	8336291	26,5%	8560890	32,6%
Rhagologus leucostigma	body	45067	64217516	2311	51,5%	35,6%	15,9%	13,0%	35,5%	4915	11658667	37,1%	9370776	35,7%
Rhea americana	body	39871	64155437	2734	70,9%	49,1%	21,8%	3,8%	25,3%	4915	6718549	21,4%	11406030	43,5%
Rhinopomastus cyanomelas	body	12476	8553110	739	13,4%	11,4%	2,0%	5,2%	81,4%	4915	2032228	6,5%	1470540	5,6%
Rhinoptilus africanus	blood	31071	48292797	2555	46,9%	30,8%	16,1%	6,8%	46,3%	4915	6152658	19,6%	7033236	26,8%
Rhipidura atra	liver	47027	72539682	2704	65,2%	36,2%	29,0%	5,6%	29,2%	4915	10649145	33,9%	11755692	44,8%
Rhipidura leucophrys	body	13190	9725872	836	11,8%	9,8%	2,0%	6,6%	81,6%	4915	2580245	8,2%	2005746	7,6%
Rhynochetos jubatus	body	43003	66612795	2660	63,7%	43,8%	19,9%	6,6%	29,7%	4915	8258055	26,3%	11104794	42,3%
Rostratula benghalensis	body	29813	40640725	2185	52,7%	37,7%	15,0%	7,1%	40,2%	4915	6221909	19,8%	8490861	32,4%
Sagittarius serpentarius	blood	55520	93452795	3154	51,1%	30,3%	20,8%	8,9%	40,0%	4915	8121529	25,9%	9301803	35,5%
Sakesphorus canadensis	body	38023	62025542	2757	63,2%	43,8%	19,4%	7,9%	28,9%	4915	9723659	31,0%	11072853	42,2%
Sapayoa aenigma	body	11789	9019462	884	10,5%	9,3%	1,2%	7,2%	82,3%	4915	2800745	8,9%	1750008	6,7%
Schiffornis veraepacis	body	20105	21636577	1516	28,2%	23,8%	4,4%	10,2%	61,6%	4915	5187943	16,5%	4783146	18,2%
Scopus umbretta	blood	46396	77392570	3001	50,5%	31,0%	19,5%	7,8%	41,7%	4915	7381070	23,5%	8853960	33,8%
Scytalopus chocoensis	body	31016	47145590	2549	58,1%	44,4%	13,7%	7,8%	34,1%	4915	9364100	29,8%	9455949	36,1%
Seiurus aurocapillus	body	33836	56919821	2791	59,7%	34,2%	25,5%	6,3%	34,0%	4915	9861725	31,4%	10063044	38,4%
Semnornis frantzii	body	33568	50117333	2455	57,6%	41,6%	16,0%	7,0%	35,4%	4915	6636517	21,1%	9398451	35,8%
Serinus canaria	brain	63503	124708835	3746	75,3%	48,7%	26,6%	7,8%	16,9%	4915	17618695	56,1%	14979852	57,1%
Serinus serinus	blood	38011	61841338	2965	42,4%	24,8%	17,6%	7,6%	50,0%	4915	7587478	24,2%	7727262	29,5%
Sitta europaea	brain	46743	81156800	3129	72,4%	48,8%	23,6%	6,1%	21,5%	4915	13957742	44,4%	13395654	51,1%
Spermophaga haematina	body	38037	53081777	2207	58,1%	39,1%	19,0%	8,6%	33,3%	4915	10003974	31,8%	10217763	39,0%
Spizella passerina	muscle	40499	58291887	2302	63,0%	39,9%	23,1%	7,1%	29,9%	4915	9847081	31,3%	11008590	42,0%
Stagonopleura guttata	blood	28315	43599878	2708	35,7%	22,0%	13,7%	6,7%	57,6%	4915	5877940	18,7%	6235668	23,8%
Steatornis caripensis	body	45693	72063696	2699	63,0%	41,7%	21,3%	7,1%	29,9%	4915	9255908	29,5%	10891050	41,5%
Stenostira scita	muscle	35656	48327450	2155	53,3%	38,1%	15,2%	9,9%	36,8%	4915	9449115	30,1%	9044535	34,5%
Stercorarius skua	body	256	151612	659	0,5%	0,4%	0,1%	0,3%	99,2%	4915	1070370	3,4%	1099029	4,2%
Streptopelia risoria	brain	63079	119916176	3714	77,5%	46,9%	30,6%	6,7%	15,8%	4915	13080891	41,6%	14584974	55,6%
Strix nebulosa	blood	47471	81268890	3147	53,5%	32,3%	21,2%	7,9%	38,6%	4915	7610061	24,2%	9273711	35,4%
Struthidea cinerea	body	29924	34989335	1702	35,3%	27,6%	7,7%	13,6%	51,1%	4915	8369354	26,6%	7437792	28,4%
Struthio camelus	brain	91283	152787413	3205	74,8%	46,1%	28,7%	7,5%	17,7%	4915	10788109	34,3%	14340351	54,7%
Sylvia atricapilla	liver	38268	57636746	2395	59,9%	39,2%	20,7%	8,2%	31,9%	4915	9670222	30,8%	10550730	40,2%
Sylvietta leucophrys	body	43979	74438395	3041	68,1%	47,1%	21,0%	6,3%	25,6%	4915	12191949	38,8%	12318546	47,0%
Tachybaptus ruficollis	blood	33813	55362799	2862	48,9%	29,4%	19,5%	5,7%	45,4%	4915	5905970	18,8%	7851870	29,9%
Taeniopygia guttata	brain	35950	49419480	2490	50,0%	42,7%	7,3%	9,0%	41,0%	4915	8212726	26,1%	9253818	35,3%
Terpsiphone rufiventer	body	28003	29928262	1458	34,2%	26,3%	7,9%	12,4%	53,4%	4915	6888589	21,9%	5991444	22,8%
Thalassoica antarctica	body	1874	1026709	554	2,4%	2,1%	0,3%	1,2%	96,4%	4915	206129	0,7%	231672	0,9%
Thalassornis leuconotus	blood	43802	73649043	3080	50,6%	30,9%	19,7%	7,1%	42,3%	4915	6066383	19,3%	8496777	32,4%
Thamnophilus palliatus	brain	61776	121764552	3841	71,9%	45,2%	26,7%	8,1%	20,0%	4915	14506690	46,2%	13991067	53,3%
Threnetes leucurus	liver	30777	46007017	2414	59,4%	46,2%	13,2%	6,2%	34,4%	4915	6909763	22,0%	9465210	36,1%
Tichodroma muraria	blood	3594	3110932	1110	6,6%	5,6%	1,0%	2,3%	91,1%	4915	590779	1,9%	956658	3,6%
Timalia pileata	liver	59063	90363713	2835	70,5%	41,4%	29,1%	5,2%	24,3%	4915	12180379	38,8%	13055022	49,8%
Todus mexicanus	body	9098	5838637	682	9,0%	7,7%	1,3%	4,3%	86,7%	4915	1434719	4,6%	1065084	4,1%
Toxorhamphus poliopterus	body	38790	51305428	1996	63,4%	40,1%	23,3%	7,3%	29,3%	4915	8963999	28,5%	10640628	40,6%
Trichoglossus moluccanus	body	36159	57457818	2687	55,8%	40,0%	15,8%	9,6%	34,6%	4915	8740775	27,8%	9918912	37,8%
Tringa totanus	blood	38924	63602885	2880	46,1%	26,9%	19,2%	7,1%	46,8%	4915	5707658	18,2%	8093592	30,9%
Troglodytes troglodytes	brain	50204	97891415	3626	79,0%	49,0%	30,0%	5,1%	15,9%	4915	15207131	48,4%	14599506	55,7%
Trogon surrucura	skin	56701	99144341	3213	74,5%	48,6%	25,9%	7,4%	18,1%	4915	11439920	36,4%	13536468	51,6%
Turnix suscitator	body	47910	86109869	3224	72,6%	45,4%	27,2%	6,9%	20,5%	4915	9279066	29,5%	13115076	50,0%
Upupa epops	blood	41559	70012992	3106	44,3%	24,3%	20,0%	6,9%	48,8%	4915	4443407	14,1%	7697514	29,3%
Uraeginthus bengalus	brain	58447	111430458	3679	74,1%	49,2%	24,9%	7,9%	18,0%	4915	16346132	52,0%	14202528	54,2%
Uraeginthus cyanocephalus	brain	65464	131277525	3914	79,4%	46,3%	33,1%	5,8%	14,8%	4915	16860296	53,7%	15525561	59,2%
Urocolius macrourus	blood	47776	76860764	3000	47,0%	25,7%	21,3%	8,7%	44,3%	4915	6004644	19,1%	8316654	31,7%
Urosphena squameiceps	muscle	35359	42168094	1727	44,0%	29,3%	14,7%	12,5%	43,5%	4915	8376944	26,7%	7830681	29,9%
Vanellus coronatus	blood	53032	92893330	3318	54,0%	29,2%	24,8%	7,4%	38,6%	4915	8273238	26,3%	9437256	36,0%
Vidua interjecta	blood	47921	78012234	3036	41,4%	25,6%	15,8%	9,0%	49,6%	4915	7577262	24,1%	8253987	31,5%
Vireo gilvus	body	28820	37843492	2045	43,6%	32,6%	11,0%	10,4%	46,0%	4915	8397957	26,7%	7295475	27,8%
Zeledonia coronata	body	31070	42455334	2100	53,9%	39,3%	14,6%	8,5%	37,6%	4915	8957139	28,5%	8658177	33,0%
Zosterops senegalensis	blood	28664	44160117	2730	39,3%	26,1%	13,2%	6,4%	54,3%	4915	5631212	17,9%	7104930	27,1%

Tab. S2B Publically available transcriptomes

Species name	Origin	BASIC STATISTICS			BUSCO ANALYSIS RESULTS						MULTIPLE ALIGNMENT ANALYSIS			
		assembled transcripts	total length [bp]	transcript N50 [bp]	complete genes	complete (single copy)	complete (two copies)	fragmented genes	body genes	searched aves_odb9 genes	ref. 3'UTR covered	relative to best	ref. CDS covered	relative to best
Acanthis fla	NCBI	58850	68623129	1900	56,4%	32,4%	24,0%	11,6%	32,0%	4915	11836355	37,7%	12185439	46,5%
Acanthis ho	NCBI	57191	61797224	1668	53,2%	31,2%	22,0%	12,2%	34,6%	4915	10649589	33,9%	11924997	45,5%
Accipiter vir	NCBI	181793	257852259	2852	81,1%	26,5%	54,6%	6,8%	12,1%	4915	9344260	29,7%	9500760	36,2%
Aegypius m	NCBI	191603	261699627	2699	81,2%	29,5%	51,7%	6,8%	12,0%	4915	9614397	30,6%	9650148	36,8%
Aerodramus	NCBI	84683	132830253	3059	75,8%	36,2%	39,6%	6,2%	18,0%	4915	6543518	20,8%	7815075	29,8%
Aerodramus	NCBI	47749	78762241	3014	71,9%	37,4%	34,5%	5,0%	23,1%	4915	5389469	17,2%	7022613	26,8%
Alectoris ru	NCBI	223464	149611487	728	39,4%	29,1%	10,3%	18,7%	41,9%	4915	3885183	12,4%	4711011	18,0%
Anas platyrh	NCBI	129176	167448620	2388	83,1%	29,7%	53,4%	4,6%	12,3%	4915	9677242	30,8%	15508761	59,1%
Anser cygno	NCBI	255244	260957235	1714	77,3%	28,1%	49,2%	6,8%	15,9%	4915	5746726	18,3%	8741580	33,3%
Apteryx ow	NCBI	128264	190040317	2833	61,2%	24,5%	36,7%	7,6%	31,2%	4915	9541050	30,4%	11577252	44,1%
Apteryx row	NCBI	127807	186665735	2813	60,5%	24,3%	36,2%	6,8%	32,7%	4915	9488807	30,2%	11689620	44,6%
Apus affinis	NCBI	84888	131039232	3036	80,3%	37,2%	43,1%	4,1%	15,6%	4915	6439324	20,5%	8205702	31,3%
Aquila chrys	NCBI	108782	155250059	2589	75,7%	39,7%	36,0%	7,1%	17,2%	4915	9074086	28,9%	8036763	30,6%
Arborophila	NCBI	99900	149798675	2853	73,3%	34,5%	38,8%	7,2%	19,5%	4915	4903765	15,6%	7878306	30,0%
Archilochus	NCBI	44953	33564103	880	31,9%	20,7%	11,2%	19,0%	49,1%	4915	1539712	4,9%	4469352	17,0%
Asio otus	NCBI	180608	255896073	2829	78,0%	27,9%	50,1%	7,1%	14,9%	4915	9332367	29,7%	9510408	36,3%
Athene noct	NCBI	174681	251476833	2899	80,2%	29,5%	50,7%	6,4%	13,4%	4915	9406284	29,9%	9541791	36,4%
Bubo bubo	NCBI	173261	240520610	2828	78,5%	30,0%	48,5%	7,6%	13,9%	4915	9251660	29,4%	9430452	36,0%
Butastur ind	NCBI	126285	117253086	1399	63,5%	30,1%	33,4%	11,9%	24,6%	4915	5738404	18,3%	7234701	27,6%
Buteo butec	NCBI	175145	266046884	3227	80,2%	28,0%	52,2%	6,8%	13,0%	4915	6707665	21,4%	6510639	24,8%
Cairina mos	NCBI	133575	205952955	3114	74,5%	32,4%	42,1%	8,9%	16,6%	4915	4773557	15,2%	5989080	22,8%
Calidris pug	NCBI	11295	7019242	681	2,5%	2,1%	0,4%	2,1%	95,4%	4915	1520842	4,8%	375030	1,4%
Calypte ann	NCBI	87292	117998546	2602	68,1%	42,0%	26,1%	11,2%	20,7%	4915	5207473	16,6%	5542398	21,1%
Catharus us	NCBI	124792	188183158	2865	80,5%	28,4%	52,1%	5,9%	13,6%	4915	17258582	54,9%	16274616	62,1%
Chrysoloph	NCBI	64147	66627913	1498	56,2%	32,9%	23,3%	14,9%	28,9%	4915	2135372	6,8%	4783842	18,2%
Circus mela	NCBI	197985	272764394	2829	80,7%	27,4%	53,3%	6,6%	12,7%	4915	6651439	21,2%	6586233	25,1%
Columba livi	NCBI	86202	77008011	1209	52,3%	28,8%	23,5%	16,4%	31,3%	4915	5004231	15,9%	12956124	49,4%
Corvus coro	NCBI	101693	163042592	3372	79,2%	40,6%	38,6%	5,0%	15,8%	4915	17289056	55,0%	15474003	59,0%
Corvus mac	NCBI	175689	119523742	706	36,8%	22,7%	14,1%	15,9%	47,3%	4915	8594649	27,4%	8608491	32,8%
Coturnix jap	NCBI	125913	193940572	3057	78,2%	29,6%	48,6%	6,6%	15,2%	4915	3822605	12,2%	5855766	22,3%
Cyanistes ca	NCBI	60739	107559950	3300	72,2%	45,1%	27,1%	7,3%	20,5%	4915	15229804	48,5%	13886433	52,9%
Cyanopica c	NCBI	170038	162706823	1437	38,4%	19,4%	19,0%	14,3%	47,3%	4915	6089055	19,4%	5091180	19,4%
Elanus caer	NCBI	204469	291795232	2937	78,8%	27,0%	51,8%	7,7%	13,5%	4915	6732317	21,4%	6558549	25,0%
Emberiza m	NCBI	23218	14839051	667	9,5%	7,5%	2,0%	10,2%	80,3%	4915	2182271	6,9%	3861792	14,7%
Eudyptes ch	NCBI	49476	69398572	2408	60,3%	34,7%	25,6%	6,9%	32,8%	4915	3504739	11,2%	4281408	16,3%
Eudyptes ch	NCBI	26176	23696890	1244	25,0%	17,7%	7,3%	11,9%	63,1%	4915	1870319	6,0%	1877940	7,2%
Eudyptes fill	NCBI	45399	61462075	2267	56,5%	32,6%	23,9%	7,2%	36,3%	4915	3319849	10,6%	3924669	15,0%
Falco subbu	NCBI	213713	308706509	2957	78,4%	25,9%	52,5%	7,1%	14,5%	4915	6600332	21,0%	6585648	25,1%
Falco tinnur	NCBI	206992	290693652	2834	78,6%	25,3%	53,3%	7,4%	14,0%	4915	16635656	53,0%	16793658	64,0%
Ficedula alb	NCBI	83457	58922936	847	47,3%	33,6%	13,7%	14,8%	37,9%	4915	7507573	23,9%	11099670	42,3%
Gallinago m	NCBI	16743	9991425	634	6,5%	3,7%	2,8%	5,2%	88,3%	4915	1284905	4,1%	1343130	5,1%
Gallus gallu	NCBI	53548	85709192	2880	70,0%	31,8%	38,2%	6,0%	24,0%	4915	7353896	23,4%	12166308	46,4%
Haemorhou	NCBI	69458	81180648	1837	68,8%	33,6%	35,2%	8,9%	22,3%	4915	10581866	33,7%	13943046	53,2%
Junco hyem	NCBI	56406	80896421	2452	59,5%	28,8%	30,7%	5,9%	34,6%	4915	10284196	32,7%	11074071	42,2%
Lamprotorn	NCBI	287444	266951847	1302	61,9%	36,0%	25,9%	14,7%	23,4%	4915	14209197	45,2%	14219628	54,2%
Lepidothrix	NCBI	97728	141804866	2721	78,0%	34,3%	43,7%	6,7%	15,3%	4915	15056885	47,9%	15011769	57,2%
Leucophaeu	NCBI	26040	38220678	2294	62,0%	48,1%	13,9%	5,6%	32,4%	4915	7391141	23,5%	8712054	33,2%
Loxia curvir	NCBI	7982	4097787	477	0,8%	0,7%	0,1%	1,6%	97,6%	4915	313554	1,0%	576819	2,2%
Lyrurus tetr	NCBI	10301	5392831	494	4,8%	3,5%	1,3%	5,8%	89,4%	4915	593514	1,9%	1275429	4,9%
Macronecte	NCBI	334138	216837573	675	28,8%	13,7%	15,1%	13,8%	57,4%	4915	10000716	31,8%	9359016	35,7%
Malurus lan	NCBI	35239	48676225	2235	57,4%	33,8%	23,6%	6,5%	36,1%	4915	8265951	26,3%	9548100	36,4%
Meleagris g	NCBI	200574	224537850	2071	76,6%	28,1%	48,5%	7,1%	16,3%	4915	8488500	27,0%	15384327	58,7%
Melopsittac	NCBI	70036	113199976	2791	73,8%	31,1%	42,7%	5,4%	20,8%	4915	10564226	33,6%	12413091	47,3%
Melospiza m	NCBI	89458	142243916	3162	76,5%	35,7%	40,8%	4,5%	19,0%	4915	16618664	52,9%	14924091	56,9%
Nesoptilotis	NCBI	46181	76162993	2870	69,5%	33,5%	36,0%	5,0%	25,5%	4915	10759409	34,2%	12225708	46,6%
Nothoproct	NCBI	78979	56440741	839	37,7%	26,0%	11,7%	24,7%	37,6%	4915	5452268	17,4%	10677897	40,7%
Oenanthe o	NCBI	67741	66007772	1317	46,3%	23,0%	23,3%	17,9%	35,8%	4915	10607953	33,8%	9676017	36,9%
Otus bakkar	NCBI	100651	155134377	2968	82,8%	37,0%	45,8%	5,4%	11,8%	4915	14531343	46,3%	16089708	61,3%
Otus scops	NCBI	186743	256264467	2634	78,0%	23,7%	54,3%	8,5%	13,5%	4915	15931332	50,7%	16716990	63,7%
Pandion hal	NCBI	28629	37457649	1920	52,7%	41,5%	11,2%	8,7%	38,6%	4915	8046779	25,6%	6964434	26,6%
Patagioenas	NCBI	73791	75735643	1706	59,3%	46,7%	12,6%	14,9%	25,8%	4915	11000610	35,0%	14123472	53,9%
Phasianus c	NCBI	233361	258809790	2074	81,3%	28,6%	52,7%	6,3%	12,4%	4915	9353406	29,8%	15952215	60,8%
Phylloscopu	NCBI	59616	46269102	962	20,7%	13,9%	6,8%	15,4%	63,9%	4915	7497370	23,9%	4485090	17,1%
Phylloscopu	NCBI	52696	40379463	950	20,2%	13,8%	6,4%	13,4%	66,4%	4915	6995348	22,3%	3993612	15,2%
Picus canus	NCBI	176786	220899328	2427	69,4%	29,6%	39,8%	12,0%	18,6%	4915	12541253	39,9%	16214340	61,8%
Pipra filicau	NCBI	86600	132512943	2916	73,6%	37,5%	36,1%	6,0%	20,4%	4915	13960411	44,4%	14443923	55,1%
Platycercus	NCBI	55863	81676155	2604	67,8%	34,5%	33,3%	5,6%	26,6%	4915	8875858	28,3%	11779677	44,9%
Poecile atri	NCBI	50488	69954448	2352	58,0%	31,3%	26,7%	6,4%	35,6%	4915	9387514	29,9%	10681662	40,7%
Pseudopod	NCBI	43109	65910150	2784	69,8%	43,8%	26,0%	4,8%	25,4%	4915	11146762	35,5%	12554295	47,9%
Pygoscelis a	NCBI	15018	12593201	1080	15,9%	11,8%	4,1%	8,0%	76,1%	4915	2770399	8,8%	2903691	11,1%
Pygoscelis p	NCBI	19099	18412124	1401	24,6%	18,4%	6,2%	11,1%	64,3%	4915	4070001	13,0%	4441824	16,9%
Sericornis fr	NCBI	47605	73415624	2591	66,6%	26,4%	40,2%	5,6%	27,8%	4915	9777238	31,1%	11628717	44,3%
Sinosuthora	NCBI	145727	90652129	670	18,2%	9,5%	8,7%	16,1%	65,7%	4915	9971752	31,7%	9334764	35,6%
Spinus spin	NCBI	90073	81335026	1277	36,0%	20,0%	16,0%	9,5%	54,5%	4915	7356953	23,4%	6656076	25,4%
Spinus tristi	NCBI	42190	61452238	2489	55,6%	30,2%	25,4%	6,6%	37,8%	4915	9400102	29,9%	10325355	39,4%
Sturnus vulg	NCBI	49078	56891667	1838	51,3%	31,6%	19,7%	9,4%	39,3%	4915	8887440	28,3%	8834550	33,7%
Syrmaticus	NCBI	72722	114036314	2942	54,2%	22,7%	31,5%	5,7%	40,1%	4915	5665190	18,0%	9048540	34,5%
Tyto longim	NCBI	173252	230249603	2555	79,1%	29,6%	49,5%	6,8%	14,1%	4915	15366641	48,9%	16312800	62,2%
Uraeginthus	NCBI	100445	101535050	1469	29,7%	15,3%	14,4%	18,4%	51,9%	4915	12476349	39,7%	8501217	32,4%
Zonotrichia	NCBI	67742	119292827	3321	80,0%	33,1%	46,9%	4,4%	15,6%	4915	15865882	50,5%	15247719	58,1%

Tab. S2C Asssembled Genomes (MPIO)

Species name	English species name	Family-level taxon	Order-level taxon	BASIC STATISTICS Contigs	total length [bp]	Contig N50 [bp]	MULTIPLE ALIGNMENT ANALYSIS ref. 3'UTR covered	relative to best	ref. CDS covered	relative to best
Erythrocercus livingstonei	Livingstone's Flycatcher	Erythrocercidae	Passeriformes	240906	1111336811	31890	25014839	79,6%	20815581	66,3%
Eulacestoma nigropectus	Wattled Ploughbill	Eulacestomidae	Passeriformes	147201	1092700062	42436	25338629	80,7%	20873253	66,4%
Hyliota australis	Southern Hyliota	Hyliotidae	Passeriformes	468218	1231942596	10121	25093966	79,9%	20861811	66,4%
Ibidorhyncha struthersii	Ibisbill	Ibidorhynchidae	Charadriiformes	222362	1282615657	28323	26947581	85,8%	23579349	75,1%
Pluvianellus socialis	Magellanic Plover	Pluvianellidae	Charadriiformes	192308	1215332984	20255	21763954	69,3%	19943475	63,5%
Ptilorrhoa leucosticta	Spotted Jewel-Babbler	Cinclosomatidae	Passeriformes	385799	1161722018	13211	25249796	80,4%	20849379	66,4%
Pnoepyga pusilla	Pygmy Wren-Babbler	Pnoepygidae	Passeriformes	397389	1210429367	9899	29676619	94,5%	24383910	77,6%

Tab. S2D

Statistical comparison of transcriptomes of brain, liver, blood, musle and body tissues (Tab. S2A)

assembled transcripts

	Analysis of variance	Least Sq Mean	Std Error	Mean
blood	B	41858,743	1478,797	41858,7
body	C	32666,884	1458,858	32666,9
brain	A	60042,488	2411,182	60042,5
liver	BC	42572,25	3452,287	42572,3
muscle	BC	35297,182	3291,627	35297,2
skin	ABC	51297	7684,615	51297

F(5, 302)=20.2135 P<.0001

complete genes

	Analysis of variance	Least Sq Mean	Std Error	Mean
blood	C	0,457	0,015	0,457
body	C	0,463	0,015	0,463
brain	A	0,717	0,025	0,717
liver	B	0,592	0,035	0,592
muscle	BC	0,512	0,034	0,512
skin	AB	0,748	0,078	0,748

F(5, 302)=21.6775 P<.0001

total length [bp]

	Analysis of variance	Least Sq Mean	Std Error	Mean
blood	B	67998179	2747503	67998179
body	C	47831832	2710456	47831832
brain	A	109654112	4479809	1,1E+08
liver	BC	61473431	6414111	61473431
muscle	BC	50796023	6115615	50796023
skin	AB	89905660	14278960	89905660

F(5, 302)=30.1404 P<.0001

ref 3'UTR covered [bp]

	Analysis of variance	Least Sq Mean	Std Error	Mean
blood	C/D	6777523	259863,4	6777523
body	BC/CD	7435366	256359,4	7435366
brain	A	13410812	423707,7	13410812
liver	B/BC	9070775	606657,1	9070775
muscle	B/BC	8602367	578424,9	8602367
skin	AB	12552876	1350521	12552876

F(5, 302)=40.9262 P<.0001

transcript N50 [bp]

	Analysis of variance	Least Sq Mean	Std Error	Mean
blood	B	2923,6422	57,71007	2923,64
body	C/D	2062,0089	56,93192	2062,01
brain	A	3356,6098	94,09638	3356,61
liver	C/CD	2352,7	134,7255	2352,7
muscle	C/D	2113,6364	128,4557	2113,64
skin	ABC	3090	299,9422	3090

F(5, 302)=41.4062 P<.0001

ref CDS covered [bp]

	Analysis of variance	Least Sq Mean	Std Error	Mean
blood	C/D	8085368	279932,2	8085368
body	C/D	7942934	276157,6	7942934
brain	A	13527664	456430	13527664
liver	B/BC	10188997	653508,3	10188997
muscle	BC/CD	8738055	623095,7	8738055
skin	AB	13594595	1454102	13594595

F(5, 302)=27.6908 P<.0001

Tab. S2E

Gappiness dependent size of transcriptomes

			Gappiness:	10	20	40	60	80	90	100	110	120	140	160	180	200
**species**	**family**	**order**	**tissue**						**number of aligned nucleotides**							
Actophilornis africanus	Jacanidae	Charadriiformes	blood	598	31020	370260	1107294	1897107	2228685	2516565	2773032	2995042	3371974	3663388	3884702	4046592
Aegithalos caudatus	Aegithalidae	Passeriformes	blood	598	31190	369215	1092309	1831544	2124492	2368526	2580956	2764136	3052248	3257956	3417187	3529142
Agapornis personatus	Psittacidae	Psittaciformes	blood	598	31150	373814	1121352	1911412	2235005	2513536	2761350	2975567	3325253	3594528	3814770	3977499
Ailuroedus buccoides	Ptilonorhynchidae	Passeriformes	blood	598	31248	375639	1128642	1930166	2262190	2546668	2802180	3026636	3397719	3673065	3883382	4040011
Alectura lathami	Megapodidae	Galliformes	blood	598	31086	372646	1111760	1892286	2214329	2491035	2737306	2953181	3328670	3637003	3886034	4063486
Amadina erythrocephala	Estrildidae	Passeriformes	blood	598	31252	372497	1112171	1882067	2189563	2448735	2676394	2866227	3177702	3400388	3556740	3662682
Amadina fasciata	Estrildidae	Passeriformes	blood	598	31196	376213	1127610	1927984	2257272	2537889	2785024	2995775	3345524	3602983	3790796	3929831
Anseranas semipalmata	Anseranatidae	Anseriformes	blood	598	31248	376089	1133578	1954074	2301579	2606777	2884966	3129566	3543455	3877693	4143520	4347931
Bombycilla garrulus	Bombycillidae	Passeriformes	blood	598	31157	372963	1109772	1891068	2210907	2483883	2729466	2940797	3284450	3547362	3751275	3904986
Buceros bicornis	Bucerotidae	Bucerotiformes	blood	598	31139	372140	1118032	1925095	2265529	2567818	2842324	3087626	3514746	3855575	4132222	4343952
Bugeranus carunculatus	Gruidae	Gruiformes	blood	598	31263	377577	1139759	1973030	2326570	2640710	2929133	3185333	3618264	3955925	4232733	4447746
Buphagus erythrorhynchus	Buphagidae	Passeriformes	blood	598	31230	371806	1103958	1852431	2143996	2380075	2582533	2753473	3019023	3203415	3329366	3414806
Burhinus capensis	Burhinidae	Charadriiformes	blood	598	31261	378199	1147009	1993307	2356308	2681392	2981940	3251021	3722313	4108258	4425420	4672851
Butorides striata	Ardeidae	Pelecaniformes	blood	598	31097	375607	1128839	1932728	2267320	2557168	2814560	3038695	3405310	3678895	3887324	4042912
Cacatua ducorpsii	Cacatuidae	Psittaciformes	blood	598	31233	373362	1123021	1923239	2251199	2534397	2789259	3010148	3372064	3651425	3879211	4042245
Cardinalis cardinalis	Cardinaldidae	Passeriformes	blood	598	31267	378623	1147251	1986498	2343049	2657219	2945535	3200424	3629481	3973355	4250097	4470244
Carduelis cannabina	Fringillidae	Passeriformes	blood	598	31265	375703	1126582	1907700	2215904	2471919	2696560	2887260	3188302	3401032	3553414	3664349
Cariama cristata	Cariamidae	Cariamiformes	blood	598	31249	376275	1135294	1956617	2303088	2610909	2890945	3138688	3562886	3895348	4158021	4352808
Cereopsis novaehollandiae	Anatidae	Anseriformes	blood	598	31228	376540	1132195	1939918	2277980	2572342	2835210	3068101	3468811	3795047	4058662	4261653
Certhia brachydactyla	Certhiidae	Passeriformes	blood	598	31234	371026	1098557	1855955	2158760	2415373	2641919	2835810	3151913	3394161	3579892	3716121
Charadrius hiaticula	Charadriidae	Charadriiformes	blood	598	31254	377963	1142853	1974307	2328472	2638762	2921848	3168794	3594032	3918660	4178569	4379570
Chauna torquata	Anhimidae	Anseriformes	blood	598	31143	375462	1132921	1953341	2300494	2604836	2882878	3127960	3544711	3887472	4159940	4359061
Chloris chloris	Fringillidae	Passeriformes	blood	598	31226	369813	1094013	1836142	2125675	2361860	2561779	2728511	2991539	3176718	3306332	3396920
Chloropsis cochinchinensis	Chloropseidae	Passeriformes	blood	598	30962	371377	1103381	1866715	2173935	2429325	2654245	2843389	3150908	3376426	3542416	3663638
Cinclus cinclus	Cinclidae	Passeriformes	blood	598	31182	367955	1083366	1821234	2111047	2348784	2552799	2724631	2999629	3196266	3339639	3447376
Cisticola galactotes	Cisticolidae	Passeriformes	blood	597	31238	374417	1122810	1915279	2242553	2517762	2761026	2969255	3310312	3567543	3765525	3916306
Clytospiza monteiri	Estrildidae	Passeriformes	blood	598	31206	377289	1137392	1962114	2305444	2601991	2870000	3106770	3502857	3811714	4048638	4230034
Coccothraustes coccothraustes	Fringillidae	Passeriformes	blood	598	29902	354828	1040823	1721623	1980362	2184870	2356222	2502783	2726420	2879866	2992309	3072131
Colinus virginianus	Odontophoridae	Galliformes	blood	598	30864	361953	1054463	1733790	1992188	2207024	2394011	2550868	2798727	2997224	3153902	3258945
Coracias caudatus	Coraciidae	Coraciiformes	blood	598	30947	372998	1119447	1917861	2248611	2534146	2787562	3008227	3374302	3652687	3869352	4029526
Crypturellus tataupa	Tinamidae	Struthioniformes	blood	596	31099	362807	1057932	1752864	2023597	2255141	2465358	2650590	2970354	3242774	3467206	3643358
Cyanoramphus novaezelandiae	Psittacidae	Psittaciformes	blood	598	31000	373141	1118084	1899956	2218743	2488896	2729117	2933137	3270652	3531296	3746708	3905400
Dacelo novaeguineae	Alcedinidae	Coraciiformes	blood	598	31211	374389	1124601	1930623	2268675	2562783	2826370	3053638	3431810	3725738	3957593	4127159
Dicrurus adsimilis	Dicruridae	Passeriformes	blood	598	31256	376465	1137045	1960204	2308556	2610575	2885942	3124057	3523314	3830562	4065144	4237089
Emberiza citrinella	Emberizidae	Passeriformes	blood	598	31123	375598	1131766	1938396	2269333	2551730	2809243	3033095	3394985	3671103	3884194	4044389
Erythrura psittacea	Estrildidae	Passeriformes	blood	597	31211	370593	1094188	1844459	2146513	2398942	2619991	2804883	3104219	3324503	3482254	3596385
Eudromia elegans	Tinamidae	Struthioniformes	blood	598	31025	359642	1048530	1724281	1983444	2205834	2402014	2570813	2860688	3091710	3279137	3423951
Euodice cantans	Estrildidae	Passeriformes	blood	558	30973	375555	1140345	1976303	2329568	2640586	2924653	3175214	3600739	3943517	4216273	4431736
Falco sparverius	Falconidae	Falconiformes	blood	598	31238	377864	1140407	1964713	2309330	2614615	2889320	3131866	3536124	3849609	4088588	4267358
Falculea palliata	Vangidae	Passeriformes	blood	598	31267	376524	1134310	1936389	2264565	2543440	2794510	3012087	3366222	3633797	3833573	3980217
Garrulax leucolophus	Leiothrichidae	Passeriformes	blood	586	31009	376930	1138965	1958063	2299801	2594642	2863406	3102545	3509940	3834794	4091854	4283335
Geokichla dohertyi	Turdidae	Passeriformes	blood	598	31157	371004	1103680	1875879	2185740	2446488	2678737	2878682	3203276	3451725	3633195	3768154
Geronticus eremita	Threskiornithidae	Pelecaniformes	blood	598	31243	378084	1146816	1988104	2346989	2666211	2960627	3225201	3681583	4045716	4343353	4569779
Gymnoderus foetidus	Cotingidae	Passeriformes	blood	597	31231	375814	1130646	1945610	2283790	2572548	2829559	3050924	3410005	3680923	3883387	4028671
Himantopus himantopus	Recurvirostridae	Charadriiformes	blood	598	31258	375869	1132630	1940268	2278180	2575396	2842682	3075878	3456715	3746460	3976766	4148540
Hypargos niveoguttatus	Estrildidae	Passeriformes	blood	598	31184	377903	1148986	2002853	2371877	2703012	3011366	3292528	3791789	4223527	4583357	4868020
Hypocolius ampelinus	Hypocoliidae	Passeriformes	blood	598	31130	376946	1134384	1947974	2283321	2568694	2820558	3040797	3406195	3676381	3871002	4011540
Irena puella	Irenidae	Passeriformes	blood	598	31230	374185	1120037	1891075	2198676	2448370	2663181	2840468	3116656	3311017	3442945	3532482
Lagonosticta rubricata	Estrildidae	Passeriformes	blood	598	31260	378049	1142085	1965958	2312148	2612832	2883840	3118284	3511022	3818828	4062366	4241164
Lagopus lagopus	Phasianidae	Galliformes	blood	598	31196	370193	1092732	1815554	2097567	2329923	2531991	2701110	2973354	3185545	3352993	3469898
Laniarius funebris	Malaconotidae	Passeriformes	blood	598	31159	376718	1137259	1957065	2298708	2589554	2854549	3083975	3465411	3755886	3981837	4157068
Larus crassirostris	Laridae	Charadriiformes	blood	598	31203	376873	1135438	1950843	2288648	2581967	2846818	3074406	3455001	3738329	3954069	4109442
Laterallus leucopyrrhus	Rallidae	Gruiformes	blood	598	30998	371268	1118702	1929612	2269829	2570569	2844073	3086411	3497804	3825397	4086493	4286103
Leiothrix lutea	Leiothrichidae	Passeriformes	blood	598	31250	375098	1120152	1897551	2214415	2478794	2708852	2906236	3230784	3468127	3646323	3776972
Leptosomus discolor	Leptosomidae	Leptosomiformes	blood	598	30988	375262	1136817	1967333	2317153	2621786	2893351	3129970	3526339	3831478	4062905	4227448
Leucopsar rothschildi	Sturnidae	Passeriformes	blood	598	31015	376630	1140638	1978007	2336918	2653070	2943705	3206443	3658620	4037579	4347763	4589618
Lophophanes cristatus	Paridae	Passeriformes	blood	598	31274	374193	1123206	1906645	2224751	2494363	2730760	2932657	3253565	3495466	3665289	3788856
Megalaima asiatica	Megalaimidae	Piciformes	blood	598	30860	353006	1019781	1675503	1930502	2140446	2324912	2481939	2723289	2893980	3026978	3126692
Morus bassanus	Sulidae	Suliformes	blood	598	31230	377072	1142687	1980666	2340153	2660578	2955930	3218253	3676414	4047775	4350048	4580053
Motacilla flava	Motacillidae	Passeriformes	blood	598	31128	373000	1110863	1877837	2186005	2440855	2665198	2854136	3152764	3366341	3526083	3642915
Musophaga violacea	Musophagidae	Musophagiformes	blood	598	31203	376536	1140231	1981106	2344602	2671488	2973510	3243530	3720570	4108675	4432944	4683873
Nectarinia tacazze	Nectariniidae	Passeriformes	blood	598	31206	378568	1145626	1976679	2325433	2630566	2907585	3151346	3560881	3879485	4126946	4310664
Neochmia temporalis	Estrildidae	Passeriformes	blood	598	31261	376783	1129208	1921465	2241766	2510142	2748555	2952994	3278548	3513812	3683825	3806429
Nestor meridionalis	Strigopidae	Psittaciformes	blood	598	31257	375733	1128939	1937196	2273878	2566427	2829318	3057186	3438195	3745618	3996718	4181554
Oriolus oriolus	Oriolidae	Passeriformes	blood	598	31254	376150	1129745	1930156	2254338	2527290	2771255	2984520	3334723	3592360	3787152	3930400
Ortygospiza atricollis	Estrildidae	Passeriformes	blood	598	31136	376412	1137970	1961294	2307153	2603899	2870672	3104993	3488127	3776117	3996141	4160708
Otis tarda	Otididae	Otidiformes	blood	598	31236	376970	1138492	1968212	2322356	2637442	2925554	3181799	3619499	3965882	4249835	4471623
Panurus biarmicus	Panuridae	Passeriformes	blood	598	31238	367324	1074345	1784870	2061256	2287759	2484096	2646383	2907100	3087252	3216693	3316389
Parabuteo unicinctus	Accipitridae	Accipitriformes	blood	598	31277	378271	1141763	1969644	2316607	2618831	2883840	3113096	3484569	3755639	3957117	4102381
Pauxi pauxi	Cracidae	Galliformes	blood	592	31215	373230	1112458	1883491	2197734	2469796	2712428	2924050	3279232	3565025	3792868	3966373
Pelecanus onocrotalus	Pelecanidae	Pelecaniformes	blood	598	31154	376729	1138008	1966734	2318269	2624678	2904879	3154198	3583106	3923132	4204527	4412878
Periparus ater	Paridae	Passeriformes	blood	598	31263	377216	1134451	1944309	2280031	2565845	2825668	3054353	3425571	3701815	3906153	4055198
Phodilus badius	Tytonidae	Strigiformes	blood	598	31273	378143	1141916	1983476	2343591	2664732	2961100	3222750	3668071	4030258	4328089	4561207
Phoenicopterus roseus	Phoenicopteridae	Phoenicopteriformes	blood	598	31269	376542	1144546	1985872	2345278	2662959	2952217	3206411	3639193	3973809	4237499	4429882
Plectropterus gambensis	Anatidae	Anseriformes	blood	598	31212	371182	1108618	1876538	2187292	2452015	2683288	2879542	3209422	3463336	3663498	3810493
Pluvianus aegyptius	Pluvianidae	Charadriiformes	blood	598	31159	377120	1139709	1968643	2321913	2634083	2918894	3170198	3602686	3953082	4236048	4444608
Podiceps nigricollis	Podicipedidae	Podicipediformes	blood	598	30876	376678	1138285	1967281	2317888	2627904	2908523	3156620	3579378	3915216	4187441	4394722
Poecile montanus	Paridae	Passeriformes	blood	598	30971	367710	1085081	1815110	2103520	2338031	2542389	2715947	2983754	3177868	3316028	3416223
Poephila acuticauda	Estrildidae	Passeriformes	blood	598	31220	375501	1130819	1931320	2257729	2532991	2776486	2986175	3328660	3576831	3755580	3889407
Poephila personata	Estrildidae	Passeriformes	blood	598	31244	378070	1146566	1984451	2341134	2653372	2939913	3190928	3622170	3973846	4245351	4450530
Pogoniulus atroflavus	Lybiidae	Piciformes	blood	598	30481	333712	947955	1535706	1758873	1941406	2096473	2222975	2410186	2546265	2651485	2730090
Promerops cafer	Promeropidae	Passeriformes	blood	598	31238	370845	1105683	1864651	2166015	2416653	2638537	2825080	3130379	3351118	3510774	3626999
Prunella modularis	Prunellidae	Passeriformes	blood	598	31067	369020	1096455	1855803	2154511	2401765	2621254	2807979	3112755	3333756	3495866	3617897
Psilopogon pyrolophus	Megalaimidae	Piciformes	blood	598	30716	353628	1035023	1715454	1983627	2211313	2411771	2581707	2852844	3053302	3216790	3340167
Psittacus erithacus	Psittacidae	Psittaciformes	blood	598	31209	374539	1123794	1928512	2263285	2552185	2812109	3038331	3410089	3704473	3943482	4121805
Psophia crepitans	Psophiidae	Gruiformes	blood	598	31080	374063	1130388	1953333	2297207	2600069	2874743	3116646	3516723	3821303	4066013	4256422
Pterocles orientalis	Pteroclididae	Pterocliformes	blood	598	31167	376204	1128703	1938871	2277323	2572661	2841056	3076476	3477000	3784691	4030690	4213073
Pycnonotus xanthopygos	Pycnonotidae	Passeriformes	blood	597	31255	370052	1097134	1846799	2146222	2397835	2621323	2815649	3131914	3365522	3543005	3680093
Pytilia phoenicoptera	Estrildidae	Passeriformes	blood	598	31255	377085	1139823	1961813	2301375	2592933	2853801	3078829	3449069	3728158	3939940	4092404
Ramphastos toco	Ramphastidae	Piciformes	blood	598	31180	365104	1072870	1795965	2086701	2332368	2551674	2737090	3027720	3247217	3421040	3550429
Regulus regulus	Regulidae	Passeriformes	blood	598	31235	377510	1143921	1992978	2363801	2699628	3012906	3301610	3842453	4367226	4887183	5358760
Remiz pendulinus	Remizidae	Passeriformes	blood	598	31162	377105	1139305	1969447	2320601	2631247	2914786	3165505	3588429	3922610	4190111	4397203
Rhinoptilus africanus	Glareolidae	Charadriiformes	blood	587	31103	375794	1133475	1948234	2289154	2584882	2846365	3072289	3441180	3715706	3924208	4075107
Sagittarius serpentarius	Sagittariidae	Accipitriformes	blood	598	31242	377463	1141023	1978619	2335168	2653168	2945228	3206617	3654022	4012686	4299925	4523162
Scopus umbretta	Scopidae	Pelecaniformes	blood	598	30980	374469	1134117	1964408	2313893	2622453	2902832	3150305	3575105	3920299	4205651	4418264
Serinus serinus	Fringillidae	Passeriformes	blood	598	31271	378373	1140465	1954157	2286875	2572017	2825097	3043236	3402521	3672631	3877340	4029457
Stagonopleura guttata	Estrildidae	Passeriformes	blood	598	31208	370033	1091513	1827837	2113243	2347953	2548976	2716408	2977632	3167971	3302797	3396275
Strix nebulosa	Strigidae	Strigiformes	blood	598	31267	376507	1141369	1979188	2338171	2655274	2944833	3198330	3630709	3984729	4272678	4487740
Tachybaptus ruficollis	Podicipedidae	Podicipediformes	blood	598	31232	372879	1111679	1901958	2227030	2507799	2752473	2961204	3302756	3553787	3753374	3899855
Thalassornis leuconotus	Anatidae	Anseriformes	blood	598	31243	374121	1121911	1919594	2250336	2534632	2787213	3007411	3387371	3691066	3935802	4107191
Tichodroma muraria	Tichodromidae	Passeriformes	blood	488	14715	115447	252328	348688	374736	391495	404064	413334	425844	431863	434867	437573
Tringa totanus	Scolopacidae	Charadriiformes	blood	598	31202	371790	1109063	1879847	2193300	2462560	2695541	2891899	3213523	3453286	3632080	3760788
Upupa epops	Upupidae	Bucerotiformes	blood	582	30130	347551	1014424	1667362	1918251	2128157	2310417	2463610	2714188	2902983	3043301	3153106
Urocolius macrourus	Coliidae	Coliiformes	blood	598	30979	365772	1087371	1843219	2155853	2426615	2668790	2879827	3236735	3518631	3740755	3907325

Vanellus coronatus	Charadriidae	Charadriiformes	blood	598	31173	377351	1145362	1992554	2359057	2685793	2986052	3257609	3732494	4116578	4440430	4688133
Vidua interjecta	Viduidae	Passeriformes	blood	598	31090	374232	1122860	1922269	2249154	2525444	2770468	2982363	3329601	3585970	3784476	3937408
Zosterops senegalensis	Zosteropidae	Passeriformes	blood	598	31258	366556	1080015	1805186	2087320	2319128	2524481	2695959	2971399	3168848	3309996	3413124
			**mean**	**596**	**30985**	**370514**	**1108925**	**1892313**	**2214980**	**2492548**	**2741187**	**2957292**	**3317115**	**3595449**	**3813604**	**3977239**
			**SD**	**11**	**1601**	**25807**	**89717**	**171327**	**211074**	**249401**	**286955**	**322618**	**389880**	**450838**	**506350**	**552917**
Abroscopus superciliaris	Scotocercidae	Passeriformes	body	598	31272	377696	1143159	1976231	2332555	2650074	2936836	3195055	3657544	4084742	4477206	4796576
Acanthiza pusilla	Acanthizidae	Passeriformes	body	598	31264	377023	1141346	1983355	2348225	2676465	2979171	3257912	3776228	4273264	4755233	5181921
Acrocephalus palustris	Acrocephalidae	Passeriformes	body	598	31246	378533	1149743	2005874	2378830	2715148	3026663	3314516	3844029	4348983	4834666	5263376
Aegithina tiphia	Aegithinidae	Passeriformes	body	598	31257	377631	1144323	1988315	2350329	2670867	2967006	3236651	3715648	4161329	4565489	4900481
Alcippe morrisonia	Pellorneidae	Passeriformes	body	598	31260	378346	1149362	2004654	2378830	2717339	3033891	3327957	3880105	4414373	4930168	5390133
Alle alle	Alcidae	Charadriiformes	body	598	31192	373864	1096893	1816772	2102378	2351271	2570224	2770874	3132830	3468925	3764392	4004840
Anastomus lamelligerus	Ciconiidae	Ciconiiformes	body	598	31263	378074	1144186	1985690	2345628	2668555	2966457	3240330	3743296	4215610	4652101	5024580
Anhinga anhinga	Anhingidae	Suliformes	body	598	25556	201712	459151	639364	704461	753582	793963	828919	891671	949103	994505	1030151
Aramus guarauna	Aramidae	Gruiformes	body	598	31243	377239	1135310	1945298	2283879	2579349	2846504	3087489	3522301	3925639	4307921	4630275
Ardenna tenuirostris	Procellariidae	Procellariiformes	body	598	31264	378484	1149398	2000685	2370631	2700608	3003146	3280640	3793475	4281310	4742498	5141898
Attagis gayi	Thinocoridae	Charadriiformes	body	598	31245	373499	1107028	1856277	2156203	2409920	2626449	2814754	3144505	3441489	3696763	3891872
Balaeniceps rex	Balaenicipitidae	Pelecaniformes	body	598	22394	164841	370195	516370	570522	614649	650195	681363	734449	786793	822924	852256
Bucco tamatia	Bucconidae	Piciformes	body	598	30795	369860	1105620	1892556	2224092	2518117	2787406	3031846	3479397	3894198	4273549	4586790
Calcarius lapponicus	Calcariidae	Passeriformes	body	598	31267	378792	1151186	2005739	2377309	2712207	3022065	3307602	3829395	4323199	4801259	5213775
Calliphlox amethystina	Trochilidae	Caprimulgiformes	body	598	31267	377036	1136520	1958318	2304767	2612968	2896815	3153626	3629219	4088468	4530958	4915835
Caprimulgus inornatus	Caprimulgidae	Caprimulgiformes	body	598	31211	376124	1128211	1937403	2281615	2587443	2868056	3124068	3589197	4018366	4411395	4742148
Casuarius casuarius	Casuariidae	Struthioniformes	body	550	22184	148530	316039	431574	470272	498508	523094	545735	585693	621850	653907	673874
Centropus superciliosus	Cuculidae	Cuculiformes	body	598	30919	367937	1099812	1890665	2227218	2528347	2806362	3063658	3545763	4021419	4492491	4918756
Chionis albus	Chionidae	Charadriiformes	body	598	31268	378311	1146310	1985001	2345085	2664601	2958035	3225963	3714926	4172708	4601142	4972295
Cinclosoma clarum	Cinclosomatidae	Passeriformes	body	598	31278	377740	1130437	1929578	2261961	2551531	2809361	3042616	3458108	3843718	4186869	4449480
Climacteris picumnus	Climacteridae	Passeriformes	body	598	31228	376657	1136070	1957283	2304095	2611769	2889203	3138493	3590127	4008397	4386783	4688416
Cnemophilus loriae	Cnemophilidae	Passeriformes	body	598	31271	378452	1149505	1999812	2367611	2696798	3000832	3276994	3782343	4261267	4715053	5100618
Coereba flaveola	Coerebidae	Passeriformes	body	598	31266	377786	1129807	1927257	2254871	2543979	2801694	3033708	3454078	3841407	4198494	4488023
Conopophaga lineata	Conopophagidae	Passeriformes	body	558	31010	376783	1140897	1982553	2346370	2673735	2978934	3262249	3790688	4300104	4787490	5222272
Coracina novaehollandiae	Campephagidae	Passeriformes	body	598	31271	360854	985169	1551383	1762210	1941903	2096048	2238726	2495893	2745436	2970530	3147834
Dendrocopos major	Picidae	Piciformes	body	598	30556	360798	1072252	1820778	2132289	2409240	2664360	2899501	3334500	3756100	4168712	4532794
Dicrurus paradiseus	Dicruridae	Passeriformes	body	598	31259	378487	1150382	2006935	2380979	2719912	3036934	3329790	3879105	4408052	4909465	5350411
Donacobius atricapillus	Donacobiidae	Passeriformes	body	598	31250	377859	1141804	1973122	2326789	2643275	2929708	3189959	3662287	4108814	4530928	4895350
Dromaius novaehollandiae	Casuariidae	Struthioniformes	body	598	31186	373539	1113986	1895383	2216325	2495190	2745947	2973286	3389974	3778686	4134240	4435619
Dromas ardeola	Dromadidae	Charadriiformes	body	598	31265	377619	1135844	1955922	2305815	2611714	2892950	3147103	3610149	4042024	4443527	4780651
Elminia albonotata	Stenostiridae	Passeriformes	body	598	31238	376998	1142921	1985506	2349681	2674869	2976577	3254296	3764639	4243619	4688904	5057607
Eubucco versicolor	Capitonidae	Piciformes	body	598	30757	312788	799495	1175478	1305959	1413468	1502135	1580454	1709077	1819706	1913945	1987579
Eurylaimus ochromalus	Eurylaimidae	Passeriformes	body	598	31176	376726	1138240	1971910	2330875	2654325	2954778	3228162	3729038	4196728	4631322	4997548
Eurypyga helias	Eurypygidae	Eurypygiformes	body	598	30899	368183	1103955	1891576	2220985	2510820	2777673	3023361	3473310	3900255	4299082	4644879
Eurystomus glaucurus	Coraciidae	Coraciiformes	body	598	31151	376186	1134924	1949502	2290437	2592284	2865353	3111632	3556469	3967903	4355765	4685159
Euschistospiza dybowskii	Estrildidae	Passeriformes	body	598	31260	364667	1004049	1572772	1778244	1949418	2093953	2225919	2460112	2672041	2852895	2981374
Fregata magnificens	Fregatidae	Suliformes	body	597	31244	375734	1112985	1870793	2181894	2451975	2691212	2907460	3298640	3667887	4008127	4279938
Galbula ruficauda	Galbulidae	Piciformes	body	598	31137	372494	1112218	1897150	2223998	2509080	2765161	2996454	3413385	3795116	4138278	4423109
Gavia stellata	Gaviidae	Gaviiformes	body	598	31211	378263	1148783	2000644	2370729	2703349	3014280	3303621	3843038	4358813	4858103	5295974
Grallina cyanoleuca	Monarchidae	Passeriformes	body	598	31263	378332	1132843	1943177	2276740	2565484	2821721	3050870	3464322	3851098	4214096	4513486
Heliornis fulica	Heliornithidae	Gruiformes	body	594	25449	220668	507798	699893	767412	820998	862695	899015	964786	1035425	1092672	1135285
Hemiprocne mystacea	Hemiprocnidae	Caprimulgiformes	body	598	30903	350655	960011	1515779	1711654	1865408	1986472	2082509	2225097	2316736	2383419	2426006
Ifrita kowaldi	Ifritidae	Passeriformes	body	598	31272	378865	1149055	1998569	2366475	2694422	2997114	3272588	3769102	4229253	4656157	5011549
Indicator indicator	Indicatoridae	Piciformes	body	598	31070	362753	1051569	1718941	1975438	2190580	2375503	2531137	2791999	3024246	3228696	3382824
Ispidina picta	Alcedinidae	Coraciiformes	body	598	30585	360306	1050250	1728924	1993353	2217354	2409046	2581659	2894028	3190049	3459552	3662506
Lamprospiza melanoleuca	Mitrospingidae	Passeriformes	body	598	31276	378301	1147489	1990178	2352819	2676053	2971603	3242974	3731663	4190205	4622361	4994539
Lipaugus vociferans	Cotingidae	Passeriformes	body	598	31239	377138	1141784	1991146	2362101	2697730	3013249	3306260	3858486	4397299	4932733	5425877
Locustella naevia	Locustellidae	Passeriformes	body	598	30485	285117	691786	1021582	1143243	1244162	1328509	1407514	1552006	1686882	1801477	1888257
Lophorina superba	Paradisaeidae	Passeriformes	body	598	31272	378878	1146219	1981602	2337600	2649616	2930001	3180147	3616162	4027750	4402170	4707716
Malacoptila panamensis	Bucconidae	Piciformes	body	598	30667	368779	1096094	1858893	2178172	2458816	2709330	2933769	3338722	3718684	4064435	4356195
Mandingoa nitidula	Estrildidae	Passeriformes	body	597	31245	377817	1143090	1978313	2337571	2656650	2948601	3214848	3703132	4162770	4601947	4979438
Melanocharis versteri	Melanocharitidae	Passeriformes	body	598	31250	378085	1145346	1973904	2326204	2635264	2914190	3167547	3621262	4035914	4403263	4691871
Melanodryas cucullata	Petroicidae	Passeriformes	body	441	11095	70867	138591	175284	186161	193821	199924	206168	215981	223205	226990	229492
Menura novaehollandiae	Menuridae	Passeriformes	body	598	31242	377912	1139959	1965396	2314028	2622346	2900668	3155707	3619265	4055218	4448263	4764909
Micrastur ruficollis	Herpetotheriane	Falconiformes	body	598	31237	376608	1132609	1942566	2284062	2580866	2844472	3081960	3506034	3891587	4232578	4499489
Molothrus ater	Icteridae	Passeriformes	body	598	31251	379073	1151013	2006059	2378365	2711217	3018979	3302049	3816497	4303581	4764260	5152704
Momotus momota	Momotidae	Coraciiformes	body	598	31127	372774	1109040	1862184	2166536	2426418	2649280	2845670	3202921	3534677	3837235	4074893
Nicator chloris	Nicatoridae	Passeriformes	body	598	31219	377706	1141178	1970135	2324384	2638901	2922879	3182523	3647573	4078665	4481645	4831506
Numida meleagris	Numididae	Galliformes	body	595	31190	374864	1123301	1910586	2234345	2520166	2781953	3021360	3466248	3907379	4340803	4731253
Nyctibius grandis	Nyctibiidae	Caprimulgiformes	body	598	30957	339108	919912	1430655	1623775	1787771	1925006	2052116	2279352	2489773	2683015	2836186
Oceanodroma leucorhoa	Hydrobatidae	Procellariiformes	body	597	13589	90708	183518	252019	275920	296649	314320	330935	360372	385564	411300	428981
Pachycephala rufiventris	Pachycephalidae	Passeriformes	body	598	30751	304466	743877	1098546	1228352	1334245	1423806	1506071	1660713	1806051	1925114	2014040
Parmoptila jamesoni	Estrildidae	Passeriformes	body	598	31261	378359	1144160	1981782	2340548	2662262	2955412	3224596	3710579	4171816	4605003	4974580
Passer domesticus	Passeridae	Passeriformes	body	598	31247	378534	1149932	2007139	2381879	2721531	3040798	3336803	3893209	4435617	4970119	5459803
Pellorneum capistratum	Pellorneidae	Passeriformes	body	598	31250	377394	1137831	1963392	2314898	2625750	2907386	3164122	3631557	4070515	4489582	4849768
Peucedramus taeniatus	Peucedramidae	Passeriformes	body	589	13451	81738	152936	193893	207826	219064	227512	236562	253096	269205	281748	288988
Phaethon lepturus	Phaethontidae	Phaethontiformes	body	376	15598	97143	187611	241636	258584	271736	279993	286423	297371	303701	307595	310689
Phainopepla nitens	Ptilogonatidae	Passeriformes	body	598	25101	175647	367045	495662	542316	579546	610931	639779	690738	731497	763659	785521
Phoebastria immutabilis	Diomedeidae	Procellariiformes	body	598	31266	377472	1133848	1935396	2266883	2554013	2807326	3036869	3439795	3813326	4150763	4418037
Phylidonyris novaehollandiae	Meliphagidae	Passeriformes	body	598	31115	371726	1114187	1918313	2262944	2569224	2851605	3109328	3581392	4026106	4451762	4821794
Phylloscopus umbrovirens	Phylloscopidae	Passeriformes	body	598	31240	371530	1107768	1881600	2201941	2479783	2725665	2949588	3344333	3702593	4029943	4296287
Picathartes gymnocephalus	Picathartidae	Passeriformes	body	598	28778	219301	475185	637201	691949	735819	775719	811680	870579	920593	959426	987835
Pitta versicolor	Pittidae	Passeriformes	body	598	31138	374135	1123597	1930049	2274710	2581833	2857967	3110003	3567713	3997207	4392555	4728881
Platylophus galericulatus	Platylophidae	Passeriformes	body	598	31261	378583	1148386	1996113	2360936	2688421	2989509	3263108	3757396	4217513	4653774	5018124
Podargus strigoides	Podargidae	Caprimulgiformes	body	598	31092	374836	1134043	1966255	2323377	2640156	2932893	3193852	3653295	4029271	4338313	4585183
Pogoniulus bilineatus	Lybiidae	Piciformes	body	598	30553	287704	693068	985963	1085520	1164267	1227766	1284929	1381197	1469105	1541182	1595345
Polioptila albiloris	Polioptilidae	Passeriformes	body	598	31250	378365	1145237	1981457	2336331	2648821	2928297	3178054	3619640	4032316	4413224	4726080
Pomatostomus superciliosus	Pomatostomidae	Passeriformes	body	598	30996	375501	1127335	1927197	2260345	2548249	2801084	3030086	3434785	3809436	4141056	4401538
Prionops plumatus	Prionopidae	Passeriformes	body	598	31246	377684	1141151	1978960	2337964	2657523	2953965	3225096	3716929	4181653	4620495	4981976
Pterodroma baraui	Procellariidae	Procellariiformes	body	598	31267	373099	1077608	1780311	2053657	2286747	2483700	2651106	2920337	3118271	3264859	3363751
Rhagologus leucostigma	Rhagologidae	Passeriformes	body	598	31272	379265	1153066	2012501	2387016	2725098	3040809	3332834	3872068	4386729	4884480	5324503
Rhea americana	Rheidae	Struthioniformes	body	598	31136	369432	1095599	1860942	2178750	2458718	2715206	2946911	3370624	3766475	4136152	4453453
Rhinopomastus cyanomelas	Phoeniculidae	Bucerotiformes	body	596	29934	283771	670839	932874	1023842	1097664	1155176	1210452	1307307	1394638	1473640	1533717
Rhipidura leucophrys	Rhipiduridae	Passeriformes	body	598	27911	242545	579143	846925	941400	1021674	1091193	1154821	1266737	1369811	1450054	1506732
Rhynochetos jubatus	Rhynochetidae	Eurypygiformes	body	598	31032	371608	1116002	1917468	2257307	2560097	2836328	3088574	3550510	3990174	4415476	4775387
Rostratula benghalensis	Rostratulidae	Charadriiformes	body	598	30957	371049	1092854	1847232	2154985	2417498	2648229	2855267	3217265	3552275	3865122	4112139
Sakesphorus canadensis	Thamnophilidae	Passeriformes	body	598	31223	377346	1141463	1983458	2349434	2680372	2989083	3272989	3802263	4313700	4795135	5209142
Sapayoa aenigma	Sapayoidae	Passeriformes	body	598	30581	300614	731089	1055321	1173532	1272076	1352020	1424820	1548791	1664529	1766814	1843514
Schiffornis veraepacis	Tityridae	Passeriformes	body	598	30945	370574	1071991	1765846	2029428	2249601	2436582	2598322	2867997	3111996	3317042	3457411
Scytalopus chocoensis	Rhinocryptidae	Passeriformes	body	598	31165	377217	1142154	1983352	2345587	2669446	2969764	3247125	3750901	4228367	4684259	5073476
Seiurus aurocapillus	Parulidae	Passeriformes	body	598	31233	379061	1151492	2005609	2376916	2711396	3021263	3307057	3829670	4327768	4801480	5206800
Semnornis frantzii	Semnornithidae	Piciformes	body	598	30989	366837	1084772	1827332	2133249	2398472	2639092	2855166	3239903	3596650	3925218	4198194
Spermophaga haematina	Estrildidae	Passeriformes	body	598	31268	379141	1149361	1991768	2354276	2678735	2977027	3250653	3747082	4218834	4667337	5057708
Steatornis caripensis	Steatornithidae	Caprimulgiformes	body	593	31173	377449	1143907	1989368	2356320	2687587	2995619	3278609	3805008	4300742	4769053	5163644
Stercorarius skua	Stercorariidae	Charadriiformes	body	598	25801	187784	394080	525559	573427	609117	637735	662754	706247	746614	772428	789972
Struthidea cinerea	Struthideidae	Passeriformes	body	598	31269	377816	1138517	1954219	2297005	2596793	2863508	3104792	3534999	3933159	4297691	4592391
Sylvietta leucophrys	Macrosphenidae	Passeriformes	body	598	31251	378727	1150877	2009507	2385613	2726732	3046630	3342842	3899491	4436808	4962763	5439699
Terpsiphone rufiventer	Monarchidae	Passeriformes	body	598	31258	374360	1092561	1815624	2104326	2352852	2564912	2754705	3091635	3413415	3704739	3930562
Thalassoica antarctica	Procellariidae	Procellariiformes	body	536	7136	40243	76123	95878	101610	106129	110413	114153	119753	126030	130204	133435
Todus mexicanus	Todidae	Coraciiformes	body	598	27097	223146	502995	687106	750078	799937	838757	874444	934014	986618	1030013	1056705
Toxorhamphus poliopterus	Melanocharitidae	Passeriformes	body	598	31222	370954	1108539	1905727	2249909	2555025	2832170	3086647	3548460	3981031	4389174	4735240
Trichoglossus moluccanus	Psittacidae	Psittaciformes	body	598	31232	377352	1138941	1967860	2321594	2634238	2922957	3187049	3674855	4135364	4579770	4956353
Turnix suscitator	Turnicidae	Charadriiformes	body	597	30348	354327	1039175	1750332	2047855	2313328	2554749	2773522	3187302	3592902	3998333	4359217

Vireo gilvus	Vireonidae	Passeriformes	body	598	31234	378525	1146872	1985477	2342681	2657256	2938714	3191770	3637134	4046002	4419748	4724366
Zeledonia coronata	Zeledoniidae	Passeriformes	body	598	31274	377562	1136239	1958629	2308738	2619440	2902173	3159212	3617355	4049951	4440189	4757430
			**Mean**	**593**	**29743**	**340451**	**992509**	**1672648**	**1956735**	**2206739**	**2432728**	**2638244**	**3010492**	**3359240**	**3683358**	**3955246**
			**SD**	**28**	**4340**	**79337**	**283335**	**535700**	**648507**	**751077**	**847070**	**935232**	**1098298**	**1254304**	**1405428**	**1538521**
Accipiter nisus	Accipitridae	Accipitriformes	brain	598	31264	378567	1149289	2004454	2376728	2713853	3031488	3325743	3877438	4410220	4925153	5380044
Alauda arvensis	Alaudidae	Passeriformes	brain	598	31246	376178	1141723	1990815	2361873	2697588	3012267	3303057	3848621	4380275	4907617	5388695
Aptenodytes patagonicus	Spheniscidae	Sphenisciformes	brain	598	31267	378539	1147485	1997474	2368771	2705100	3019412	3310456	3855088	4382801	4899628	5362010
Apus apus	Apodidae	Caprimulgiformes	brain	595	30933	372259	1128568	1962303	2324632	2650784	2954242	3233854	3760646	4273600	4786678	5257470
Chroicocephalus ridibundus	Laridae	Charadriiformes	brain	598	31266	378651	1149232	2000698	2371849	2706470	3019480	3311254	3857711	4387881	4911688	5389506
Columba palumbus	Columbidae	Columbiformes	brain	598	31257	375669	1136866	1972362	2331595	2656607	2960183	3239479	3763175	4267975	4771974	5229895
Crotophaga ani	Cuculidae	Cuculiformes	brain	598	31128	375307	1134601	1970352	2333460	2661348	2966581	3249237	3778531	4291389	4798793	5265193
Emblema pictum	Estrildidae	Passeriformes	brain	598	31269	378568	1148778	1999951	2368491	2699782	3008180	3291483	3813195	4305776	4763960	5160719
Estrilda melpoda	Estrildidae	Passeriformes	brain	597	31250	378014	1145626	1991885	2359362	2688269	2993714	3275291	3789942	4273293	4721155	5101038
Fringilla coelebs	Fringillidae	Passeriformes	brain	598	31252	378612	1150600	2008299	2382967	2721333	3038345	3333117	3885327	4420735	4949428	5426900
Hirundo rustica	Hirundinidae	Passeriformes	brain	598	31228	373999	1118521	1915244	2251405	2542379	2807776	3050225	3488236	3890776	4268974	4568924
Lanius collurio	Laniidae	Passeriformes	brain	598	31269	377679	1145956	1999580	2371899	2709762	3026255	3319259	3869922	4407011	4938366	5419436
Lonchura striata	Estrildidae	Passeriformes	brain	598	31261	378536	1146537	2001911	2375002	2712479	3029315	3321940	3870261	4407339	4936952	5419474
Lophornis magnificus	Trochilidae	Caprimulgiformes	brain	598	31255	376477	1131112	1943938	2287349	2593490	2876714	3134373	3613066	4075070	4524813	4922612
Malurus melanocephalus	Maluridae	Passeriformes	brain	598	31045	374064	1134899	1980091	2349785	2684609	2998155	3287172	3828435	4349453	4863368	5329805
Manacus vitellinus	Pipridae	Passeriformes	brain	598	31256	377478	1142834	1987739	2354454	2685820	2995660	3280566	3814274	4331076	4839847	5292376
Melanerpes cruentatus	Picidae	Piciformes	brain	598	30655	359354	1071566	1820670	2134976	2415820	2675967	2914373	3359299	3791384	4223829	4612961
Myiozetetes cayanensis	Tyrannidae	Passeriformes	brain	462	19074	252560	787454	1409875	1685014	1938132	2181136	2406731	2840819	3274405	3719022	4126182
Nyctidromus albicollis	Caprimulgidae	Caprimulgiformes	brain	598	31210	377674	1143389	1988399	2355792	2685907	2994487	3279894	3811589	4327226	4833160	5288264
Nymphicus hollandicus	Cacatuidae	Psittaciformes	brain	598	31179	376337	1136990	1974519	2336690	2662082	2965917	3245913	3772528	4282451	4789606	5252914
Padda oryzivora	Estrildidae	Passeriformes	brain	598	31259	377887	1143148	1981619	2340703	2660694	2955951	3225406	3713624	4161410	4571258	4911202
Parus major	Paridae	Passeriformes	brain	598	31267	379013	1150880	2008075	2382727	2721053	3038069	3332174	3887049	4426721	4960484	5447218
Phalacrocorax carbo	Phalacrocoracidae	Suliformes	brain	598	31227	377552	1141270	1987265	2355323	2687161	2998709	3288235	3830801	4355185	4873623	5341471
Pheugopedius genibarbis	Troglodytidae	Passeriformes	brain	598	31245	378681	1148515	2004065	2378011	2716864	3034791	3329241	3883824	4423849	4958148	5445066
Phoenicurus ochruros	Muscicapidae	Passeriformes	brain	598	31253	378284	1148020	2001764	2374643	2712521	3029944	3324206	3878052	4417940	4955545	5446657
Picus viridis	Picidae	Piciformes	brain	598	30675	362374	1078036	1831907	2146118	2424111	2681706	2916870	3350238	3757446	4152252	4498358
Ploceus bicolor	Ploceidae	Passeriformes	brain	598	31254	378941	1150263	2007785	2382980	2723033	3041719	3337073	3889308	4420757	4944969	5419037
Pyrrhula pyrrhula	Fringillidae	Passeriformes	brain	598	31252	377531	1144915	1997303	2369150	2705754	3020424	3312652	3861609	4394703	4921283	5401686
Ramphocelus carbo	Thraupidae	Passeriformes	brain	598	31272	378791	1149124	2007417	2383022	2723448	3043034	3339450	3895118	4436206	4970992	5455318
Serinus canaria	Fringillidae	Passeriformes	brain	598	31275	379351	1153633	2016253	2394715	2737830	3060031	3359796	3923277	4473954	5021454	5522401
Sitta europaea	Sittidae	Passeriformes	brain	598	31235	377943	1144840	1993410	2362533	2695576	3007957	3295822	3830818	4347602	4853616	5310755
Streptopelia risoria	Columbidae	Columbiformes	brain	598	31250	374018	1131233	1962867	2319633	2642742	2944318	3222873	3744929	4249245	4754243	5215090
Struthio camelus	Struthionidae	Struthioniformes	brain	598	31279	379661	1155639	2021704	2402025	2747698	3073075	3376313	3949802	4511696	5070977	5589050
Thamnophilus palliatus	Thamnophilidae	Passeriformes	brain	598	31203	375839	1135113	1976219	2343820	2676227	2987706	3275129	3815497	4343344	4863037	5334045
Troglodytes troglodytes	Troglodytidae	Passeriformes	brain	598	31219	378535	1147817	2001302	2373930	2711307	3027733	3321635	3873681	4410502	4939114	5420824
Uraeginthus bengalus	Estrildidae	Passeriformes	brain	598	31269	377878	1146007	2001360	2375872	2714886	3033118	3328476	3882610	4424437	4962787	5454041
Uraeginthus cyanocephalus	Estrildidae	Passeriformes	brain	598	31258	378748	1150627	2009095	2384149	2723725	3042848	3339664	3898126	4445158	4988094	5486267
			**mean**	**594**	**30872**	**373123**	**1130030**	**1965675**	**2328418**	**2655574**	**2961525**	**3244282**	**3773148**	**4284602**	**4787456**	**5240349**
			**SD**	**22**	**1999**	**20793**	**60478**	**103220**	**123104**	**141583**	**159053**	**176109**	**209035**	**243364**	**279257**	**318808**
Anthracothorax nigricollis	Trochilidae	Caprimulgiformes	liver	598	31205	376449	1135177	1956198	2303771	2612911	2896699	3154006	3628163	4074575	4489292	4834301
Artamus cyanopterus	Artamidae	Passeriformes	liver	598	31253	378877	1148218	1996808	2363626	2691704	2995426	3271439	3777747	4245793	4674147	5034473
Atelornis pittoides	Brachypteraciidae	Coraciiformes	liver	598	30617	370748	1119316	1938425	2290521	2606963	2900268	3168303	3671207	4155371	4628524	5046585
Campylopterus largipennis	Trochilidae	Caprimulgiformes	liver	598	31231	374982	1121433	1909344	2233415	2517701	2774143	2999810	3405762	3783141	4129287	4402187
Corcorax melanorhamphos	Struthideidae	Passeriformes	liver	598	31264	370711	1071521	1758400	2025209	2256817	2463025	2649820	2993630	3324381	3626057	3868655
Larosterna inca	Sternidae	Charadriiformes	liver	598	31244	376483	1130076	1938907	2275603	2572022	2841918	3085460	3522783	3924653	4292805	4592636
Microeca papuana	Petroicidae	Passeriformes	liver	598	31199	378385	1150698	2008769	2383801	2723449	3042327	3337065	3890049	4424460	4941183	5404205
Phaethornis ruber	Trochilidae	Caprimulgiformes	liver	598	31221	377577	1138204	1963466	2313100	2623914	2910030	3171592	3651243	4112892	4552945	4935429
Philomachus pugnax	Scolopacidae	Charadriiformes	liver	598	31256	375796	1123527	1918559	2251867	2546148	2809496	3047321	3477212	3876297	4242444	4540308
Plocepasser mahali	Ploceidae	Passeriformes	liver	597	31222	377740	1141953	1976492	2333869	2651858	2941013	3206713	3688488	4151011	4581991	4944397
Poecile palustris	Paridae	Passeriformes	liver	598	31249	375023	1113699	1896901	2223730	2506686	2761878	2989576	3393627	3762133	4095991	4353928
Pteroptochos megapodius	Rhinocryptidae	Passeriformes	liver	598	31238	377333	1141715	1980247	2341537	2662957	2961427	3233450	3727051	4184862	4610594	4962985
Pteruthius flaviscapis	Vireonidae	Passeriformes	liver	598	31129	377600	1149571	2008656	2384389	2725455	3046196	3344283	3902851	4447258	4984427	5469264
Rhipidura atra	Rhipiduridae	Passeriformes	liver	598	31227	378193	1148187	1994941	2363970	2697582	3007560	3292372	3819617	4326508	4807701	5227524
Sylvia atricapilla	Sylviidae	Passeriformes	liver	598	31241	378376	1148008	1998245	2365392	2695064	2999393	3279550	3793337	4280064	4741519	5131180
Timalia pileata	Timaliidae	Passeriformes	liver	598	31267	378407	1150060	2006329	2380727	2719016	3035319	3328904	3880553	4413992	4934678	5401291
			**mean**	**598**	**31191**	**376418**	**1133210**	**1953168**	**2302158**	**2613140**	**2899132**	**3159979**	**3638958**	**4092962**	**4520849**	**4884334**
			**SD**	**0**	**157**	**2521**	**20535**	**63619**	**91223**	**119367**	**149104**	**178724**	**237905**	**298804**	**365762**	**437491**
Aegotheles cristatus	Aegothelidae	Caprimulgiformes	muscle	598	18917	118241	241029	311672	337291	356465	371851	385593	409228	431325	451880	462889
Amandava amandava	Estrildidae	Passeriformes	muscle	598	31239	378679	1148971	2001283	2372369	2707721	3021248	3311113	3851185	4375573	4897057	5368120
Cathartes melambrotus	Cathartidae	Cathartiformes	muscle	598	31259	378560	1147425	1991666	2356135	2681762	2983202	3259871	3768125	4248460	4704866	5097632
Cettia diphone	Cettidae	Passeriformes	muscle	598	31262	376934	1134171	1955448	2308132	2623471	2908811	3169196	3640762	4081231	4494632	4850347
Chaetops aurantius	Chaetopidae	Passeriformes	muscle	598	31185	374980	1128454	1946481	2293078	2603070	2886395	3142423	3602506	4032388	4433215	4778410
Chloebia gouldiae	Estrildidae	Passeriformes	muscle	598	31062	378163	1149711	2004545	2377294	2714283	3030088	3323100	3870332	4398834	4914233	5377522
Cracticus tibicen	Artamidae	Passeriformes	muscle	598	31261	379070	1151621	2007835	2381780	2717517	3028005	3314065	3845433	4353622	4840058	5257090
Daphoenositta chrysoptera	Neosittidae	Passeriformes	muscle	598	31259	373973	1114782	1896306	2221500	2507894	2759141	2987225	3381581	3748093	4081495	4356740
Dicaeum geelvinkianum	Dicaeidae	Passeriformes	muscle	598	29050	244339	568815	788024	869083	935843	987614	1036695	1118747	1190414	1243309	1282077
Hylia prasina	Hyliidae	Passeriformes	muscle	598	31268	378311	1148624	1998876	2369302	2702515	3013584	3300276	3831229	4336320	4814922	5225055
Milvago chimachima	Polyborini	Falconiformes	muscle	598	31171	377048	1133363	1937942	2276405	2574225	2842820	3084741	3520124	3928383	4301129	4605599
Oceanites oceanicus	Oceanitidae	Procellariiformes	muscle	598	31243	378282	1148022	1998474	2369883	2705774	3018941	3309280	3851875	4374707	4881535	5333912
Opisthocomus hoazin	Opisthocomidae	Opisthocomiformes	muscle	598	31224	376791	1138178	1969800	2323648	2636917	2926935	3192013	3674936	4124575	4545511	4894948
Pardalotus punctatus	Pardalotidae	Passeriformes	muscle	598	31260	378695	1149918	2005563	2378019	2712384	3021783	3305376	3830321	4329836	4806652	5219289
Pedionomus torquatus	Pedionomidae	Charadriiformes	muscle	584	19513	118750	227229	295481	320536	341394	356659	371603	401719	431853	452383	466581
Phonygammus keraudrenii	Paradisaeidae	Passeriformes	muscle	598	31253	378776	1145183	1976343	2329764	2636557	2912549	3159039	3594288	3997590	4373339	4678818
Platysteira castanea	Platysteiridae	Passeriformes	muscle	598	31256	379053	1152354	2012445	2388199	2729245	3049012	3345962	3901377	4437497	4960499	5427336
Psophodes olivaceus	Psophodidae	Passeriformes	muscle	598	31258	378426	1135959	1928192	2250919	2527018	2769848	2986431	3364929	3719640	4033222	4284652
Ptiloris magnificus	Paradisaeidae	Passeriformes	muscle	598	31268	379315	1152481	2006180	2377368	2708559	3014163	3292718	3806400	4292662	4750493	5144789
Spizella passerina	Passerellidae	Passeriformes	muscle	598	31261	378016	1142698	1980985	2342920	2666836	2963527	3237100	3733774	4207814	4663409	5058464
Stenostira scita	Stenostiridae	Passeriformes	muscle	598	31238	378431	1145434	1985293	2348335	2671907	2965997	3230492	3706135	4155620	4581025	4942535
Urosphena squameiceps	Scotocercidae	Passeriformes	muscle	598	31264	377743	1125334	1917200	2244701	2530005	2783609	3015015	3427312	3815291	4183573	4491909
			**mean**	**597**	**30044**	**348208**	**1033171**	**1768911**	**2083485**	**2363244**	**2618899**	**2852697**	**3278742**	**3682351**	**4064020**	**4391123**
			**SD**	**3**	**3537**	**79600**	**286068**	**538241**	**649366**	**749960**	**844425**	**930969**	**1091085**	**1244784**	**1395295**	**1529134**

Tab. S3

Tab. S3A. TFBS - Family-level comparisons

Higher Taxon	Species and family-level taxon
Estrildidae	
	Amandava amandava Estrildidae
	Clytospiza monteiri Estrildidae
	Emblema pictum Estrildidae
	Erythrura psittacea Estrildidae
	Euodice cantans Estrildidae
	Heteromunia pectoralis Estrildidae
	Hypargos niveoguttatus Estrildidae
	Mandingoa nitidula Estrildidae
	Ortygospiza atricollis Estrildidae
	Spremophaga haematina Estrildidae
Fringillidae	
	Acanthis flamea Fringillidae
	Carduelis cannabina Fringillidae
	Chloris chloris Fringillidae
	Coccothraustes coccothraustes Fringillidae
	fringillla coelebs Fringillidae
	Haemorhous mexicanus Fringillidae
	Phyrrula phyrrula Fringillidae
	Serinus canaria Fringillidae
	Serinus serinus Fringillidae
	Spinus tristis Fringillidae
Basal Oscines	
	Ailuroedus buccoides Ptilonorhynchidae
	Cinclosoma clarum Cinclosomatidae
	Climacteris picumnus Climacteridae
	Coracina novaehollandiae Campephagidae
	Gerygone flavolateralis Acanthizidae
	Malurus melanocephalus Maluridae
	Menura novaehollandiae Menuridae
	Pardalotus punctatus Pardalotidae
	Phylidonyris novaehollandiae Meliphagidae
	Pomatostomus superciliosus Pomatostomidae

Higher Taxon	Species and family-level taxon
Charadriiformes	
	Actophilornis africanus Jacanidae
	Alle alle Alcidae
	Burhinus capensis Burhinidae
	Charadrius hiaticula Charadiidae
	Chroicocephalus ridibundus Laridae
	Dromas ardeola Dromadidae
	Himatopus himatopus Recurvirostridae
	Philomachus pugnax Scolopacidae
	Pluvianus aegyptus Pluvianidae
	Vanellus coronatus Charadiidae
Caprimulgiformes	
	Antrostomus carolinensis Caprimulgidae
	Calypte anna Trochilidae
	Caprimulgus inornatus Caprimulgidae
	Chaetura pelagica Apodidae
	Florisuga fusca Trochilidae
	Nyctibius grandis Nyctibiidae
	Phaethornis ruber Trochilidae
	Podargus strigoides Podargidae
	Steatornis caripensis Steatornithidae
	Threnetes leucurus Trochilidae

Tab. S3B. List of genes of which 3'UTRs were used in TFBS Z-Score analysis (Fig. 1D).

Gene Symbol	Sequence_SCA_ID		Gene Symbol	Sequence_SCA_ID	
FAM83H	SCA	g 1033	PMPCA	SCA	g 7965
MAPK15	SCA	g 1034	FAM78A	SCA	g 8023
THUMPD2	SCA	g 1035	AIF1L	SCA	g 8025
ARHGAP39	SCA	g 1036	GPR107	SCA	g 8036
108962334	SCA	g 1062	YARS2	SCA	g 8884
EEF1D	SCA	g 1067	SRP68	SCA	g 9220
CYLD	SCA	g 1069	SPHK1	SCA	g 9227
ZC3H3	SCA	g 1072	SSH2	SCA	g 9528
CAP2	SCA	g 1094	GRPEL1	SCA	g 9790
FAM8A1	SCA	g 1095	MAN2B2	SCA	g 9795
TMEM14	SCA	g 1113	RBPJ	SCA	g 9863
EEF1E1	SCA	g 1122	LNX1	SCA	g 9964
RIOK1	SCA	g 1129	FRG1	SCA	g 10003
RPP40	SCA	g 1140	SPOCK3	SCA	g 10070
RIPK1	SCA	g 1150	INTS10	SCA	g 10160
LOC108964730 (LOC108964730), ncRNA	SCA	g 1157	GRID2	SCA	g 10244
UPF0711 protein C18orf21	SCA	g 1268	LSM6	SCA	g 10287
FAM206A	SCA	g 1307	MSRB3	SCA	g 10391
USP14	SCA	g 1443	GOLT1B	SCA	g 10529
CHD7	SCA	g 1552	SLC22A2	SCA	g 10604
RRM2B	SCA	g 1736	RIT2	SCA	g 10753
S1PR1	SCA	g 2001	PIK3C3	SCA	g 10758
RPAP2	SCA	g 2052	CASZ1	SCA	g 10911
CASK	SCA	g 3305	PPCS	SCA	g 11088
NDUFA1	SCA	g 4163	C1orf95	SCA	g 11588
SEPT6	SCA	g 4165	CNRIP1	SCA	g 11663
SLC25A5	SCA	g 4170	TRMT5	SCA	g 11793
IL1RAPL2	SCA	g 4179	ADCK1	SCA	g 11981
ZC4H2	SCA	g 4199	DDB2	SCA	g 12273
FAM155B	SCA	g 4218	MRPL21	SCA	g 12437
IGBP1	SCA	g 4223	SH3GL1	SCA	g 13193
PDZD11	SCA	g 4229	GAB1	SCA	g 13726
CYSLTR1	SCA	g 4233	TULP3	SCA	g 13948
P2RY10	SCA	g 4237	IRS2	SCA	g 14585
RBM15B	SCA	g 4906	GALNTL2	SCA	g 15820
MUSTN1	SCA	g 4927	MAN2C1	SCA	g 15990
ABHD6	SCA	g 4976	COL4A3BP	SCA	g 16058
FNTM2	SCA	g 5420	PURG	SCA	g 16263
FAM20C	SCA	g 5797	ZBTB8A	SCA	g 16345
AACS	SCA	g 6438	JAM3	SCA	g 16521
RPS6KA2	SCA	g 6569	PDZD8	SCA	g 17533
CAPN11	SCA	g 6647	DDX18	SCA	g 18454
SLC35B2	SCA	g 6651	TTLL4	SCA	g 18550
RAB23	SCA	g 6950	SLC40A1	SCA	g 18817
BEND6	SCA	g 6953	ERBB4	SCA	g 18850
TINAG	SCA	g 6963	RHOG	SCA	g 18982
RAB41	SCA	g 7288	SEMA3G	SCA	g 19028
DEPTOR	SCA	g 7732	EMC1	SCA	g 19137

Tab. S4

Comparison between molecular and fossil based age determination of avian higher clades

order-level taxa	family-lavel taxa	stem/ crown	molecular estimated timing (Mya)	dated fossil (Mya)	references
Accipitriformes	Accipitridae		26	37	Mlíkovský (2002); Mayr (2017)
Accipitriformes	Pandionidae	stem	32	36	Mlíkovský (2002); Mayr (2017)
Accipitriformes	Sagittariidae	stem	43	30	Mlíkovský (2002); Mayr (2017)
Anseriformes	Anseranatidae	stem	47	25	Worthy and Scanlon (2009)
Anseriformes	Anatidae		32	34	Mayr (2017)
Anseriformes	Anhimidae	stem	54	20-28	Mayr (2017)
Bucerotiformes	stem-Upupidae	stem	52	47	Ksepka and Clarke (2015)
Caprimulgiformes	Apodidae	stem	47	51	Ksepka and Clarke (2015)
Caprimulgiformes	Trochilidae	stem	26	32	Mayr (2004)
Caprimulgiformes	Nyctibiidae	stem	56	47	Mayr (2017)
Caprimulgiformes	Podargidae	stem	58	47	Mayr (2017)
Caprimulgiformes	Steatornithidae	stem	56	51	Mayr (2017)
Cariamiformes	Cariamidae	stem	65	47	Mayr (2017)
Charadriiformes	Alcidae	stem	19	34	Smith (2015)
Charadriiformes	Burhinidae		37	23	Smith (2015)
Charadriiformes	Jacanidae	stem	30	30	Smith (2015)
Charadriiformes	Laridae +Sternidae	stem	22	24	Mlíkovský (2002); Mayr (2017)
Charadriiformes	Pedionomidae	stem	23	24-26	De Pietri et al. (2015)
Charadriiformes	Turnicidae	stem	48	32-34	Mayr (2017)
Ciconiiformes	Ciconiidae		61	35	Miller et al. (1997)
Coliiformes	Coliidae	stem	63	56	Ksepka and Clarke (2015)
Coraciiformes	Coraciidae + Brachypteraciidae	stem	46	54	Bourdon et al. (2016)
Coraciiformes	Todidae	stem	45	35	Mlíkovský (2002); Mayr (2017)
Cuculiformes	Cuculidae	stem	55	46-48	Mourer-Chauviré (2013)
Falconiformes	Polyborini		28	5-11	Mayr (2017)
Falconiformes	Falconidae		9	11-15	Mayr (2017)
Falconiformes	Herpetotheriane	stem	40	16-18	Mayr (2017)
Galliformes	unknown	stem	63	67	Field et al., 2020
Galliformes	Phasianidae	stem	37	32-34	Mayr (2017)
Galliformes	Megapodiidae	stem	55	23-28	Mayr (2017)
Gaviiformes	Gaviidae	stem	65	41-44	Mayr and Zvonok (2011)
Gruiformes	Gruidae	stem	25	16	Göhlich (2003); Mayr (2017)
Gruiformes	Rallidae	stem	33	32-34	Mayr (2017)
Gruiformes	Heliornithidae		33	11-15	Mayr (2017)
Leptosomiformes	Leptosomidae	stem	63	51	Mayr (2017)
Musophagiformes	Musophagidae		13	32-34	Mayr (2017)
Opisthocomiformes	Opisthocomidae	stem	64	34	Mayr and De Pietri (2014)
Otidiformes	Otididae		12	5	Mayr (2017)
Passeriformes	unknown	stem	49	51	Mayr (2017)
Passeriformes	Acanthisittidae	stem	49	16-19	Worthy et al. (2010)
Passeriformes	sub-oscine families	stem	37	32-34	Mayr (2017)
Passeriformes	oscine-Families		40	32-34	Mayr (2017)
Pelecaniformes	Pelecanidae	stem	33	28	Smith and Ksepka (2015)
Pelecaniformes	Threskiornithidae	stem	56	54	Smith and Ksepka (2015)
Phaethontiformes	Phaethontidae	stem	62	56	Smith and Ksepka (2015)
Piciformes	Picidae		19	23	Mayr (2017)
Podicipediformes	Podicipedidae		14	19	Mlíkovský (2002); Mayr (2017)
Procellariiformes	unknown	stem	62	65	Mayr (2017)
Procellariiformes	Diomedeidae	stem	35	32-34	Mayr and Smith (2012)
Procellariiformes	Hydrobatidae		24	5-11	Mayr (2017)
Procellariiformes	Procellariidae		30	41-33	Mayr (2017)
Psittaciformes	unknown	stem	62	54	Ksepka and Clarke (2015)
Psittaciformes	Cacatuidae		17	11-16	Boles (1993)
Psittaciformes	Psittacidae		29	20-23	Mayr (2017)
Psittaciformes	Strigopidae	stem	44	16-19	Worthy et al. (2011)
Pterocliformes	Pteroclidae	stem	19	25	Mourer-Chauviré (1993)
Sphenisciformes	Spheniscidae	stem	62	61	Slack et al. (2006)
Strigiformes	unknown	stem	64	56-59	Mayr (2017)
Strigiformes	Strigidae		24	28-33	Mayr (2017)
Strigiformes	Tytonidae		20	15	Mayr (2017)
Struthioniformes	Apterygidae		12	16-19	Worthy et al. (2013)
Struthioniformes	Casuariidae + Dromaiinae	stem	25	24	Mayr (2017)
Struthioniformes	Struthionidae	stem	58	20	Mourer-Chauviré et al. (1996)
Struthioniformes	Tinamidae		27	16	Bertelli et al. (2014)
Suliformes	Phalacrocoracidae		24	25	Smith and Ksepka (2015)
Suliformes	Anhingidae	stem	24	24-26	Worthy (2012)
Suliformes	Fregatidae	stem	49	52	Smith and Ksepka (2015)
Trogoniformes	Trogonidae	stem	61	54	Kristoffersen (2002)

References

Bertelli S, Chiappe LM, Mayr G. 2014. Phylogenetic interrelationships of living and extinct Tinamidae, volant palaeognathous birds from the New World. Zoological Journal of the Linnean Society, 172, 145-184.

Boles WE. 1993. A new cockatoo (Psittaciformes: Cacatuidae) from the Tertiary of Riversleigh, northwestern Queensland, and an evaluation of rostral characters in the systematics of parrots. Ibis, 135(1), 8-18.

Kristoffersen AV. 2002. An early Paleogene trogon (Aves: Trogoniformes) from the Fur Formation, Denmark. Journal of vertebrate Paleontology, 22(3), 661-666.

Bourdon E, Kristoffersen AV, Bonde N. 2016. A roller-like bird (Coracii) from the Early Eocene of Denmark. Scientific reports, 6, 34050.

De Pietri VL, Mayr G. 2014. Reappraisal of early Miocene rails (Aves, Rallidae) from central France: diversity and character evolution. Journal of Zoological Systematics and Evolutionary Research, 52, 312-322.

De Pietri VL, Camens AB, Worthy TH. 2015. A Plains-wanderer (Pedionomidae) that did not wander plains: a new species from the Oligocene of South Australia. Ibis 157: 68-74.

Field DJ, Benito J, Chen A, Jagt JW, Ksepka DT. 2020. Late Cretaceous neornithine from Europe illuminates the origins of crown birds. Nature 579: 397-401.

Göhlich U B. 2003. A new crane (Aves: Gruidae) from the Miocene of Germany. Journal of Vertebrate Paleontology, 23(2), 387-393.

Ksepka DT, Clarke J. 2015. Phylogenetically vetted and stratigraphically constrained fossil calibrations within Aves. Palaeontol Electronica 18(3FC): 1-25.

Mayr G. 2004. Old World fossil record of modern-type hummingbirds. Science, 304(5672), 861-864.

Mayr G. 2017. Avian evolution: the fossil record of birds and its paleobiological significance. John Wiley & Sons, Chichester.

Mayr G, Smith T. 2012. A fossil albatross from the early Oligocene of the North Sea Basin. Auk 129: 87-95.

Mayr G, Zvonok E. 2011. Middle Eocene Pelagornithidae and Gaviiformes (Aves) from the Ukrainian Paratethys. Palaeontology, 54(6), 1347-1359.

Miller ER, Rasmussen DT, Simons EL. 1997. Fossil storks (Ciconiidae) from the late Eocene and early Miocene of Egypt. Ostrich, 68(1), 23-26.

Mlíkovsky J. 2002. Cenozoic birds of the world. 1. Europe. Ninox Press, Praha.

Mourer-Chauviré C. 1993. Les gangas (Aves, Columbiformes, Pteroclidae) du Paléogène et du Miocène inférieur de France. Palaeovertebrata, 22, 73-98.

Mourer-Chauviré C , Tabuce R, Essid EM, Marivaux L, Khayati H, Vianey-Liaud M, Ali MBH. 2013. A new taxon of stem group Galliformes and the earliest record for stem group Cuculidae from the Eocene of Djebel Chambi, Tunisia. In Proceedings of the 8th international meeting of the Society of Avian Paleontology and Evolution Proc. R. Soc. B (Vol. 281, No. 20140765, p. 9).

Mourer-Chauviré C, Senut B, Pickford M, Mein P. 1996. Le plus ancien représentant du genre Struthio (Aves, Struthionidae), Struthio coppensi n. sp., du Miocène inférieur de Namibie. Comptes rendus de l'Académie des sciences. Série 2. Sciences de la terre et des planètes, 322(4), 325-332.

Slack KE, Jones CM, Ando T, Harrison GL, Fordyce RE, Arnason U, Penny D. 2006. Early penguin fossils, plus mitochondrial genomes, calibrate avian evolution. Molecul Biol Evol 23: 1144-1155.

Smith ND, Ksepka DT. 2015. Five well-supported fossil calibrations within the ‘Waterbird’assemblage (Tetrapoda, Aves). Palaeontol Electronica 18: 1-21.

Smith NA. 2015. Sixteen vetted fossil calibrations for divergence dating of Charadriiformes (Aves, Neognathae). Palaeontol Electronica 18: 1-18.

Worthy TH. 2012. A new species of Oligo-Miocene darter (Aves: Anhingidae) from Australia. The Auk, 129(1), 96-104.

Worthy TH, Scanlon JD. 2009. An Oligo-Miocene magpie goose (Aves: Anseranatidae) from Riversleigh, Northwestern Queensland, Australia. Journal of Vertebrate Paleontology, 29, 205-211.

Worthy TH, Hand SJ, Nguyen JMT, Tennyson AJD, Worthy JP, Scofield RP, Boles WE, Archer M. 2010. Biogeographical and phylogenetic implications of an early Miocene wren (Aves: Passeriformes: Acanthisittidae) from New Zealand. Journal of Vertebrate Paleontology, 30, 479–498.

Worthy TH, Tennyson AJ, Scofield RP. 2011. An early Miocene diversity of parrots (Aves, Strigopidae, Nestorinae) from New Zealand. Journal of Vertebrate Paleontology, 31, 1102-1116.

Worthy T, Worthy JP, Tennyson A J, Salisbury SW, Hand S J, Scofield RP. 2013. Miocene fossils show that kiwi (Apteryx, Apterygidae) are probably not phyletic dwarves. In: U.B. Göhlich & A. Kroh (eds.), Paleornithological Research 2013 - Proceedings of the 8th International Meeting of the Society of Avian Paleontology and Evolution. Vienna: Natural History Museum Vienna, pp. 63–80.

Tab. S5

Family-level taxon	Order-level taxon	References
Acanthizidae	Passeriformes	Gill F, Donsker D. 2017. IOC World Bird List (v 7.3). doi: 10.14344/IOC. ML72.
Acrocephalidae	Passeriformes	Atkin K, Townsend AD. 1965. On the problems of separating Reed and Marsh Warblers: mimicry in song of Reed Warbler. Brit Birds 58:181-182.
Aegithinidae	Passeriformes	Bharos AMK. 1998. Mimicry by common iora Aegithina tiphia. J Bombay Nat Hist Soc 95(1):116.
Alaudidae	Passeriformes	Dowsett-Lemaire F, Dowsett, RJ. 1978. Vocal mimicry in the lark Mirafra hypermetra as a possible species-isolating mechanism. Bull Brit Ornithol Club 98:140-144.
Artamidae	Passeriformes	Kaplan G. 1999. Song Structure and Function of Mimicry in the Australian Magpie (Gymnorhina tibicen): Compared to Lyrebird (Menura ssp.). Int J Comp Psychol 12(4):
Bombycillidae	Passeriformes	Chu M. 2001. Heterospecific responses to scream calls and vocal mimicry by phainopeplas (Phainopepla nitens) in distress. Behaviour 138(6):775-787.
Campephagidae	Passeriformes	Diamond JM. 2002. Dispersal, mimicry, and geographic variations in northern Melanesian birds. Pac Sci 56(1):1-22.
Cardinaldidae	Passeriformes	Yamaguchi A. 2001. Sex differences in vocal learning in birds. Nature 411:257.
Certhiidae	Passeriformes	Thielcke G. 1972. Waldbaumläufer (Certhia familiaris) ahmen artfremdes Signal nach und reagieren darauf. J Ornithol 113(3):287-296.
Cettidae	Passeriformes	Cramp SP, Perrins CM, Brooks, DJ (Eds) 1994. Handbook of the Birds of Europe the Middle East and North Africa: The Birds of the Western Paleartic. Oxford University Press
Chloropseidae	Passeriformes	Aitken EH. 1887. On the mimicry shown by Phyllornis jerdoni. J Bombay Nat Hist Soc 1(1):25.
Cisticolidae	Passeriformes	Nuttall RJ. 2000. Vocal mimicry in individual Thick-billed Lark Galerida magnirostris and Yellow-bellied Eremomela Eremomela icteropygialis. Mirafra 17:56-57.
Coerebidae	Passeriformes	Gill F, Donsker D. 2017. IOC World Bird List (v 7.3). doi: 10.14344/IOC. ML72.
Corvidae	Passeriformes	Lorenz K. 1931. Beiträge zur Ethologie sozialer Corviden. J Ornithol 79:67-127.
Dicaeidae	Passeriformes	Close DH. 1991. Mimicry by Mistletoebird. S Aust Ornithol 31(3):74.
Dicruridae	Passeriformes	Jamie GA, de Silva Wijeyratne G. 2016. Mimicry of Sri Lanka Crested Drongo Dicrurus lophorinus by Fork-tailed Drongo Cuckoo Surniculus dicruroides. Bird Asia 25:82-83.
Emberizidae	Passeriformes	Wonke G, Wallschläger D. 2009. Song dialects in the yellowhammer Emberiza citrinella: bioacoustic variation between and within dialects. J Ornithol 150:117-126.
Estrildidae	Passeriformes	Slater PJ, Eales LA, Clayton N. 1988. Song learning in zebra finches (Taeniopygia guttata): progress and prospects.In: Advances in the Study of Behavior: Elsevier. p. 1-34.
Fringillidae	Passeriformes	Lachlan RF, Slater P. 2003. Song learning by chaffinches: how accurate, and from where? Anim Behav 65(5):957-969.
Icteridae	Passeriformes	Goller M, Shizuka D. 2018. Evolutionary origins of vocal mimicry in songbirds. Evol Lett 2(4):417-426.
Irenidae	Passeriformes	Hartshorne C. 1973. Born to sing: an interpretation and world survey of bird song. A Midland Book. Indiana Unversity Press.
Laniidae	Passeriformes	Blasé B. 1960. Die Lautäußerungen des Neuntöters (Lanius c. collurio L.), Freilandbeobachtungen und Kaspar-Hauser-Versuche. Z Tierpsychol 17:293-344.
Leiothrichidae	Passeriformes	Goller M, Shizuka D. 2018. Evolutionary origins of vocal mimicry in songbirds. Evol Lett 2(4):417-426.
Locustellidae	Passeriformes	Cramp SP, Perrins CM, Brooks, DJ (Eds) 1994. Handbook of the Birds of Europe the Middle East and North Africa: The Birds of the Western Paleartic. Oxford University Press
Macrosphenidae	Passeriformes	Goller M, Shizuka D. 2018. Evolutionary origins of vocal mimicry in songbirds. Evol Lett 2(4):417-426.
Malaconotidae	Passeriformes	Wickler W. 1972. Aufbau and Paarspezifitat des Gesangsduettes von Laniarius funebris (Aves, Passeriformes, Laniidae). Z Tierpsychol 30:464-476.
Maluridae	Passeriformes	Higgins PJ, Peter JM, Cowling SJ (Eds.). 2006. Handbook of Australian, New Zealand and Antarctic Birds. Melbourne: Oxford University Press.
Meliphagidae	Passeriformes	Veerman PA. 1994. Batesian acoustic mimicry by the Regent Honeyeater Xanthomyza phrygia. Aust Bird Watcher 15(6):250-259.
Menuridae	Passeriformes	Dalziell AH, Magrath RD. 2012. Fooling the experts: accurate vocal mimicry in the song of the superb lyrebird, Menura novaehollandiae. Anim Behav 83(6):1401-1410.
Mimidae	Passeriformes	O'Loghlen AL, Rothstein SI. 2002. Ecological effects on song learning: delayed development is widespread in wild populations of brown-headed cowbirds. Anim Behav 63:475-486.
Monarchidae	Passeriformes	Gill F, Donsker D. 2017. IOC World Bird List (v 7.3). doi: 10.14344/IOC. ML72.
Motacilllidae	Passeriformes	Wallschläger D. 1984. A bioacoustical contribution to the systematics of the Palearctic Motacillidae. II. Songs and call-notes of the genus Anthus. Mitt Zool Mus Berlin 60:37-56.
Muscicapidae	Passeriformes	Hughes M, Hultsch H, Todt D. 2002. Imitation and invention in song learning in nightingales (Luscinia megarhynchos B., Turdidae). Ethology 108:97-113.
Nectariniidae	Passeriformes	Grimes L. 1974. Dialects and geographical variation in the song of the splendid sunbird Nectarinia coccinigaster. Ibis 116(3):314-329.
Nicatoridae	Passeriformes	Gill F, Donsker D. 2017. IOC World Bird List (v 7.3). doi: 10.14344/IOC. ML72.
Oriolidae	Passeriformes	Béland P. 1977. Mimicry in orioles of south-eastern Queensland. Emu 77(4):215-218.
Orthonychidae	Passeriformes	McGuire M. 1996. Dialects of the chowchilla Orthonyx spaldingii in upland rainforest of north-eastern Australia. Emu 96(3):174-180.
Pachycephalidae	Passeriformes	Armstrong EA. 1963. A study of bird song. London: Oxford University Press.
Paridae	Passeriformes	McGregor PK, Krebs JR. 1989. Song learning in adult great tits (Parus major): effects of neighbours. Behaviour 108(1-2):139-159.
Parulidae	Passeriformes	Kroodsma DE, Meservey WR, Pickert R. 1983. Vocal learning in the Parulinae. Wilson Bull 138-140.
Passerellidae	Passeriformes	Konishi M. 1965. The role of auditory feedback in the control of vocalization in the white-crowned sparrow. Z Tierpsychol 22(7):770-783.
Passeridae	Passeriformes	Wickler W. 1982. Immanuel Kant and the Song of the House Sparrow. Auk 99(3):590-591.
Phylloscopidae	Passeriformes	Thielcke G, Linsenmair KE. 1963. Zur geographischen Variation des Gesanges des Zilpzalps, Phylloscopus collybita, in Mittel-und Südwesteuropa mit einem Vergleich des Gesanges des Fitis, Phylloscopus trochilus. J Ornithol 104:372-402.
Platysteiridae	Passeriformes	Goller M, Shizuka D. 2018. Evolutionary origins of vocal mimicry in songbirds. Evol Lett 2(4):417-426.
Ploceidae	Passeriformes	Wickler W, Kleindienst HU, Sonnenschein E, Seibt U. 2002. Structure, geography and origin of dialects in the traditive song of the forest weaver Ploceus bicolor sclateri in Natal, S. Africa. Behaviour 139:1237-1265.
Polioptilidae	Passeriformes	Gill F, Donsker D. 2017. IOC World Bird List (v 7.3). doi: 10.14344/IOC. ML72.
Prunellidae	Passeriformes	Cramp SP, Perrins CM, Brooks, DJ (Eds) 1994. Handbook of the Birds of Europe the Middle East and North Africa: The Birds of the Western Paleartic. Oxford University Press
Psophodidae	Passeriformes	Diamond JM. 2002. Dispersal, mimicry, and geographic variations in northern Melanesian birds. Pac Sci 56(1):1-22.
Ptilogonatidae	Passeriformes	Chu M. 2001. Heterospecific responses to scream calls and vocal mimicry by phainopeplas (Phainopepla nitens) in distress. Behaviour 138(6):775-787.
Ptilonorhynchidae	Passeriformes	Frith CB, McGuire M. 1996. Visual evidence of vocal avian mimicry by male tooth-billed bowerbirds Scenopoeetes dentirostris (Ptilonorhynchidae). Emu 96:12-16.
Pycnonotidae	Passeriformes	Reichenow A. 1905. Die Vögel Afrikas: J. Neumann.
Regulidae	Passeriformes	Päckert M, Martens J. 2004. Song dialects on the Atlantic islands: goldcrests of the Azores (Regulus regulus azoricus, R. r. sanctae-mariae, R. r. inermis). J Ornithol 145:23-30
Stenostiridae	Passeriformes	Wood AJ. 1993. Bushveld Pipits duetting. Honeyguide 39(4):198.
Sturnidae	Passeriformes	Eens M, Pinxten R, Verheyen RF. 1992. Song learning in captive European starlings, Sturnus vulgaris. Anim Behav 44(6):1131-1143.
Sylviidae	Passeriformes	Bergmann H-H. 1973. Die Imitationsleistung einer Mischsänger-Dorngrasmücke (Sylvia communis). J Ornithol 114(3):317-338.
Thraupidae	Passeriformes	Willis EO. 1976. Similarity of a tanager (Orchesticus abeillei) and an Ovenbird (Philydor rufus): A possible case of mimicry. Ciência e Cultura 28:1492-1493.
Timaliidae	Passeriformes	Gill F, Donsker D. 2017. IOC World Bird List (v 7.3). doi: 10.14344/IOC. ML72.
Troglodytidae	Passeriformes	Kroodsma DE. 1974. Song learning, dialects, and dispersal in the Bewick's wren. Z Tierpsychol 35(4):352-380.
Turdidae	Passeriformes	Tretzel E. 1967. Imitation und Transposition menschlicher Pfiffe durch Amseln (Turdus m. meruhi L.) 1: Ein weiterer Nachweis relativen Lernens und akustischer Abstraktion bei Vögeln. Z Tierpsychol 24(2):137-161.
Vangidae	Passeriformes	Gill F, Donsker D. 2017. IOC World Bird List (v 7.3). doi: 10.14344/IOC. ML72.
Viduidae	Passeriformes	Payne RB. 1973. Vocal mimicry of the paraside whydahs (Vidua) and response of female whydahs to the songs of their hosts (Pytilia) and their mimics. Anim Behav 21:762-771.
Vireonidae	Passeriformes	Morton ES, Howlett J, Kopysh NC, Chiver I. 2006. Song ranging by incubating male Blue-headed Vireos: the importance of song representation in repertoires and implications for song delivery patterns and local/foreign dialect discrimination. J Field Ornithol 77(3):291-301.
Zosteropidae	Passeriformes	Guest SJ. 1973b. White-eye vocal mimicry. Elepaio 34:3-4.
Trochilidae	Apodiformes	Baptista LF, Schuchmann, K-L. 1990. Song Learning in the Anna Hummingbird (Calypte anna). Ethology 84 (1):15-26.
Psittacidae	Psittaciformes	Cruickshank AJ, Gautier JP, Chappuis, C. 1993. Vocal mimicry in wild African Grey Parrots Psittacus erithacus. Ibis 135(3):293-299.
Cacatuidae	Psittaciformes	Walløe S, Thomsen H, Balsby TJ, Dabelsteen T. 2015. Differences in short-term vocal learning in parrots, a comparative study. Behaviour 152(11):1433-1461.
Strigopidae	Psittaciformes	Wein A, Schwing R, Hausberger M, Rodriguez R, Huber L. 2018. Vocal conditioning in kea parrots (Nestor notabilis). J Comp Psychol 132(1):97.

Tab. S6

family-level taxon	references
Acanthizidae	Gahr, unpublished data
Acrocephalidae	Gahr, unpublished data
Aegithalidae	Moore JM, Székely T, Büki J, DeVoogd TJ. 2011. Motor pathway convergence predicts syllable repertoire size in oscine birds. Proc Natl Acad Sci 108:16440-16445.
Alaudidae	Gahr, unpublished data
Artamidae	Deng C, Kaplan G, Rogers LJ. 2001. Similarity of the song nuclei of male and female Australian magpies (Gymnorhina tibicen). Behav Brain Res. 123:89-102
Cardinaldidae	Jawor JM, Macdougall-Shackleton SA. 2008. Seasonal and sex-related variation in song control nuclei in a species with near-monomorphic song, the northern cardinal. Neurosci Lett 443:169-173.
Certhiidae	Devoogd TJ, Krebs JR, Healy SD, Purvis A. 1993. Relations between song repertoire size and the volume of brain nuclei related to song: comparative evolutionary analyses amongst oscine birds. Proc Royal Soc London B Biol Sci 254:75-82.
Cinclidae	Gahr, unpublished data
Cisticolidae	Gahr, unpublished data
Corvidae	Wang R, Sun Y, Zhang X, Zeng S, Xie W, Yu Y, Zhang X, Zuo M. 2009. Song control nuclei in male and female large-billed crows (Corvus macrorhynchos). Zoolog Sci 26:771-
Dicruridae	Gahr, unpublished data
Emberizidae	Zhao J, Jiang J, Li D. 2003. Control pattern of vocal center for vocalization in ruddy bunting (Emberiza rutila). Sci China C Life Sci 46:615-622.
Estrildidae	Gahr M, Guettinger HR, Kroodsma DE. 1993. Estrogen receptors in the avian brain: survey reveals general distribution and forebrain areas unique to songbirds. J Comp Neurol 327:112-122.
Fringillidae	Lucke J, Haase E. 1980. Autoradiography of the brain of bramblings (Fringilla montifringilla L.) following the injection of 3H-testosterone. J Hirnforsch 21:369-380.
Hirundinidae	Moore JM, Székely T, Büki J, DeVoogd TJ. 2011. Motor pathway convergence predicts syllable repertoire size in oscine birds. Proc Natl Acad Sci 108:16440-16445.
Icteridae	Hauber ME, Clayton NS, Kacelnik A, Reboreda JC, DeVoogd TJ. 1999. Sexual dimorphism and species differences in HVC volumes of cowbirds. Behav Neurosci 113:1095-
Laniidae	Moore JM, Székely T, Büki J, DeVoogd TJ. 2011. Motor pathway convergence predicts syllable repertoire size in oscine birds. Proc Natl Acad Sci 108:16440-16445.
Leiothrichidae	Gahr, unpublished data
Locustellidae	Moore JM, Székely T, Büki J, DeVoogd TJ. 2011. Motor pathway convergence predicts syllable repertoire size in oscine birds. Proc Natl Acad Sci 108:16440-16445.
Malaconotidae	Gahr M, Sonnenschein E, Wickler W. 1998. Sex difference in the size of the neural song control regions in a dueting songbird with similar song repertoire size of males and females. J Neurosci 18:1124-1131.
Maluridae	Schwabl H, Dowling J, Baldassarre DT, Gahr M, Lindsay WR, Webster MS. 2015. Variation in song system anatomy and androgen levels does not correspond to song characteristics in a tropical songbird. Animal Behav 104: 39-50.
Mimidae	McCasland JS. 1987. Neuronal control of bird song production. J Neurosci 7:23-39.
Motacilllidae	Moore JM, Székely T, Büki J, DeVoogd TJ. 2011. Motor pathway convergence predicts syllable repertoire size in oscine birds. Proc Natl Acad Sci 108:16440-16445.
Muscicapidae	Dittrich F, Ramenda C, Grillitsch D, Frankl-Vilches C, Ko MC, Hertel M, Goymann W, ter Maat A, Gahr M. 2014. Regulatory mechanisms of testosterone-stimulated song in the sensorimotor nucleus HVC of female songbirds. BMC Neurosci 15:128.
Panuridae	Moore JM, Székely T, Büki J, DeVoogd TJ. 2011. Motor pathway convergence predicts syllable repertoire size in oscine birds. Proc Natl Acad Sci 108:16440-16445.
Paridae	Gahr M, Guettinger HR, Kroodsma DE. 1993. Estrogen receptors in the avian brain: survey reveals general distribution and forebrain areas unique to songbirds. J Comp Neurol 327:112-122.
Parulidae	Moore JM, Székely T, Büki J, DeVoogd TJ. 2011. Motor pathway convergence predicts syllable repertoire size in oscine birds. Proc Natl Acad Sci 108:16440-16445.
Passerellidae	Baker MC, Bottjer SW, Arnold AP. 1984. Sexual dimorphism and lack of seasonal changes in vocal control regions of the white-crowned sparrow brain. Brain Res
Passeridae	Gahr M, Guettinger HR, Kroodsma DE. 1993. Estrogen receptors in the avian brain: survey reveals general distribution and forebrain areas unique to songbirds. J Comp Neurol 327:112-122.
Phylloscopidae	Gahr, unpublished data
Ploceidae	Gahr M, Metzdorf R, Schmidl D, Wickler W. 2008. Bi-directional sexual dimorphisms of the song control nucleus HVC in a songbird with unison song. PLoS One
Prunellidae	Devoogd TJ, Krebs JR, Healy SD, Purvis A. 1993. Relations between song repertoire size and the volume of brain nuclei related to song: comparative evolutionary analyses amongst oscine birds. Proc Royal Soc London B Biol Sci 254(1340):75-82.
Pycnonotidae	Gahr, unpublished data
Regulidae	Gahr, unpublished data
Remizidae	Gahr, unpublished data
Sittidae	Gahr, unpublished data
Sturnidae	Gahr M, Guettinger HR, Kroodsma DE. 1993. Estrogen receptors in the avian brain: survey reveals general distribution and forebrain areas unique to songbirds. J Comp Neurol 327:112-122.
Sylviidae	Moore JM, Székely T, Büki J, DeVoogd TJ. 2011. Motor pathway convergence predicts syllable repertoire size in oscine birds. Proc Natl Acad Sci 108:16440-16445.
Thraupidae	Quispe R, Sebe F, da Silva ML, Gahr M. 2016. Dawn-song onset coincides with increased HVC androgen receptor expression but is decoupled from high circulating testosterone in an equatorial songbird. Physiol Behav 156:1-7.
Timaliidae	Gahr, unpublished data
Troglodytidae	Brenowitz EA, Arnold AP. 1986. Interspecific comparisons of the size of neural song control regions and song complexity in duetting birds: evolutionary implications. J Neurosci 6:2875-2879.
Turdidae	Lorena J, Olson CR, Fontana CS, Mello CV, Schneider MPC, Schneider PN. 2019. Seasonal changes in the song control nuclei of the Rufous-bellied Thrush, Turdus rufiventris (Oscine, Passeriformes, and Turdidae). J Exp Zool B Mol Dev Evol 332(3-4):92-98.
Viduidae	Gahr, unpublished data
Zosteropidae	Gahr, unpublished data
